# Molecular Electrocatalysis Coupled With a Palladium Membrane Reactor Affords Selective Nitrogen Reduction

**DOI:** 10.1002/advs.78155

**Published:** 2026-10-06

**Authors:** Morgan McKee, Sai Phani Kumar Vangala, Farzaneh Farzinpour, Jasmin Wagner, Giacomo Pugliese, Hamed Heidarpour, Hossein Bemana, J. Felipe Estrada‐Ruiz, Jörg Daniels, Alessandro Bismuto, Ali Seifitokaldani, Nikolay Kornienko

**Affiliations:** ^1^ Institute of Inorganic Chemistry University of Bonn Bonn Germany; ^2^ Department of Chemical Engineering McGill University Montréal Québec Canada; ^3^ Department of Chemical Engineering Indian Institute of Science Education and Research (IISER) Bhopal Bhopal Madhya Pradesh India

**Keywords:** ammonia production, catalysis, electrocatalyst, electrochemistry, faraday efficiency, hydrogen, hydrogen production, membrane reactor, palladium

## Abstract

Ammonia production is a cornerstone of modern industry and society, yet the conventional Haber–Bosch process accounts for nearly 2% of the global CO_2_ emissions. A promising and potentially greener alternative is electrochemical ammonia synthesis, though effective systems in this context are largely lacking. To this end, this study develops a reactor platform that integrates a palladium membrane reactor as a hydrogen conduit coupled with a mono‐terpyridine nickel molecular catalyst. This system achieved a peak Faradaic efficiency of 36% for ammonia production at a current density of −0.85 mA cm^−2^ in a decoupled electrocatalytic manner. In situ spectroscopic analysis, electroanalytical techniques, and computational modelling have revealed a unique catalyst mechanism that entails the formation of a Ni‐H species generated from the active hydrogen from the Pd membrane as a key intermediate in the reaction route. The insights gained from this unique system stand to aid the development of practical ammonia production systems while expanding the community's knowledge base on dinitrogen activation.

## Graphical abstract

A palladium membrane reactor delivers active hydrogen to homogeneous nickel terpyridine catalysts, which then selectively hydrogenate dinitrogen to ammonia.

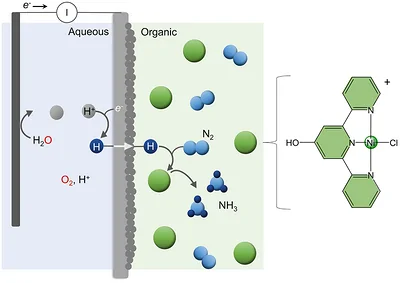

## 1 Introduction

Ammonia (NH_3_) is a vital global chemical with approximately 200 million tonnes being produced per year [1]. To date, the majority of ammonia is industrially synthesized via the Haber–Bosch (H‐B) process, which is a thermochemical route that uses hydrogen and nitrogen gas reactants and heterogeneous iron‐ or ruthenium‐based catalysts [2]. However, due to the high required temperatures (300°C–500°C) and pressures (150–300 atm), the H‐B process accounts for ∼1.5% of global energy use and correspondingly leads to ∼2% of society's greenhouse gas emissions [3]. In addition, the hydrogen gas used in the H‐B process typically comes from fossil fuel sources (e.g., steam reforming of methane).

Against this backdrop, potential greener alternatives for H‐B ammonia synthesis are being sought out. One such route is the electrochemical nitrogen reduction reaction (N_2_RR) to ammonia using electricity instead of temperature/pressure as the driving force. When the electric power is derived from renewable sources and key performance criteria are met, the carbon footprint can be substantially reduced relative to the H‐B process [4]. Correspondingly, this approach has received widespread attention in the electrochemical community, and numerous efforts have been made to understand the N_2_RR process at a mechanistic level and to subsequently develop systems with the efficiencies needed for technoeconomic viability [5, 6]. Because of the kinetically difficult reaction step of dinitrogen activation via its N≡N bond cleavage, the discovery of effective catalysts is seen as being critical [7]. A wide range of catalysts have been investigated as N_2_RR catalysts [8], ranging from heterogeneous materials [9, 10] to molecular species [7, 11, 12, 13] and to biological systems such as enzymes [14] and microbes [15].

In the context of biological systems, nitrogenase enzymes are responsible for nitrogen fixation under mild conditions [14]. They carry out this reaction through bimetallic active sites featuring Fe, Mo, or V metal species. The Mo‐dependent nitrogenase in particular has attracted significant attention. This enzyme consists of two catalytic components: the Fe protein, which facilitates electron transfer, and the MoFe protein, which binds and reduces the substrate with the aid of sulfur‐based supports [16]. The study of this enzyme has yielded insights into the activation of dinitrogen and plausible reaction pathways that can potentially be translated into practical N_2_RR systems [17]. Efforts were recently expanded toward the direct integration of a nitrogenase enzyme into electrochemical and photochemical reactors and have demonstrated the capacity for the electroreduction of azide, nitrite, and even dinitrogen reactants to ammonia [18, 19, 20].

The atomically precise active sites within enzymes naturally serve as an inspiration for molecular catalyst design, where the tunable first and second coordination spheres can be leveraged to extract key structure‐activity relationships. A recent example entails the study of a T‐shaped nickel thiolate molecular complex for the reduction of dinitrogen to hydrazine via an electrochemically generated Ni(I) active state [19]. Indirect generation of active states in molecular catalysts was also shown to be an effective strategy using a cobaltocenium redox mediator [21]. Additional studies have shown evidence for Mo‐based catalysts that operate via a bimolecular pathway [22].

Direct electrochemical N_2_ reduction remains particularly challenging because of the strong N≡N bond, low N_2_ solubility, and competition from the hydrogen evolution reaction (HER). Recent work has explored a broad range of catalyst compositions and reaction strategies to address these limitations. For example, electrodeposited Bi on Cu has been shown to exhibit enhanced N_2_RR performance following controlled surface oxidation, highlighting the importance of catalyst composition, surface structure, and dynamic restructuring in determining ammonia selectivity [23]. Compositionally complex early‐transition‐metal carbides and (carbo)nitrides have also recently been investigated as alternative N_2_RR catalysts, demonstrating the expanding range of materials platforms being explored for sustainable ammonia synthesis [24]. In parallel, metal‐mediated approaches have emerged as an alternative means of activating N_2_ by decoupling nitrogen activation from conventional proton‐coupled electrochemical reduction. Although Li‐mediated N_2_RR has achieved substantial advances in ammonia selectivity and current density, recent analyses have emphasized the importance of rigorous controls and isotope validation when evaluating Li‐N_2_ systems, as well as practical considerations associated with their operation [25]. These challenges have motivated increasing interest in alternative metal mediators and reaction concepts that can enable N_2_ activation under less demanding conditions [26].

Beyond direct N_2_RR, sustainable ammonia production is also being explored through alternative nitrogen feedstocks and reaction architectures. Electrochemical reduction of nitrate and other nitrogen‐containing species can circumvent the difficult initial N_2_ activation step and has attracted increasing attention as a route for simultaneously treating nitrogen‐containing waste streams and producing NH_3_ [27]. Recent studies have further demonstrated the potential of reaction‐environment and catalyst design to regulate proton transfer, intermediate stabilization, and competing HER, while metal‐mediated approaches using earth‐abundant elements such as Mg provide alternatives to Li‐mediated N_2_ activation [28, 29]. These developments illustrate that sustainable ammonia synthesis is increasingly moving toward strategies in which nitrogen activation, hydrogen delivery, proton transfer, and intermediate stabilization can be deliberately controlled rather than relying solely on direct N_2_ hydrogenation at a conventional electrode. In this context, molecular catalysts offer a complementary platform because their coordination environments can be systematically tuned and their reaction mechanisms investigated at the molecular level.

In contrast, complexes using terpyridine ligands have yet to be investigated for nitrogen fixation due to the lack of open coordination sites for reactants within bis‐terpyridine complexes. However, we were particularly interested in the use of a mono‐terpyridine metal complex due to its open active site with potential for binding dinitrogen and hydride reactants. Molybdenum is a common metal center for such reactions, with pincer complexes garnering attention due to having a non‐hindered and open active site [12]. Nickel metal centers, on the other hand, have not been extensively studied for the binding of dinitrogen. However, a wide range of nickel hydride complexes have been identified, with a common trend also being the use of pincer‐type ligands [30]. These two factors inspired our investigation into a mono‐terpyridine nickel complex.

We further wanted to carry out the reaction in an environment where the N_2_ solubility was higher than in water, where the competing hydrogen evolution reaction can be minimized and where we can directly deliver active hydrogen species to facilitate the reaction process. Thus, we turned to the use of a palladium membrane reactor (PMR). Such reactors feature the reduction of aqueous protons on a thin (c.a. 25 µm) metal foil, and the reduced active hydrogen (^*^H) diffuses through the foil to a non‐aqueous compartment where it can be used for hydrogenation reactions [31]. Functional examples of this system entail the hydrogenation of hydrocarbon reactants [32], nitriles [33], and even biological components such as nicotinamide adenine dinucleotide [34]. In the context of N_2_ hydrogenation, this reactor concept was shown to be viable when combined with a Li‐mediated approach [35] and direct hydrogenation when Ni foil was used in place of Pd [36].

In this study, we demonstrate the first integration of a palladium membrane reactor coupled with a homogeneous molecular catalyst (Figure 1). In this system, a mono‐hydroxy‐terpyridine nickel complex (NitpyOH) is leveraged for dinitrogen reduction with resulting Faradaic efficiencies (FEs) of 36%. A combination of in situ spectroscopy, analytical investigations, and computational modeling provides evidence that the catalyst is first activated by the Pd membrane to the Ni‐H active species. This active species is then viable as a catalyst for the reduction of dinitrogen to ammonia via a unique self‐reduction activation step that provides key mechanistic information for the scientific community to build upon.

**FIGURE 1:**
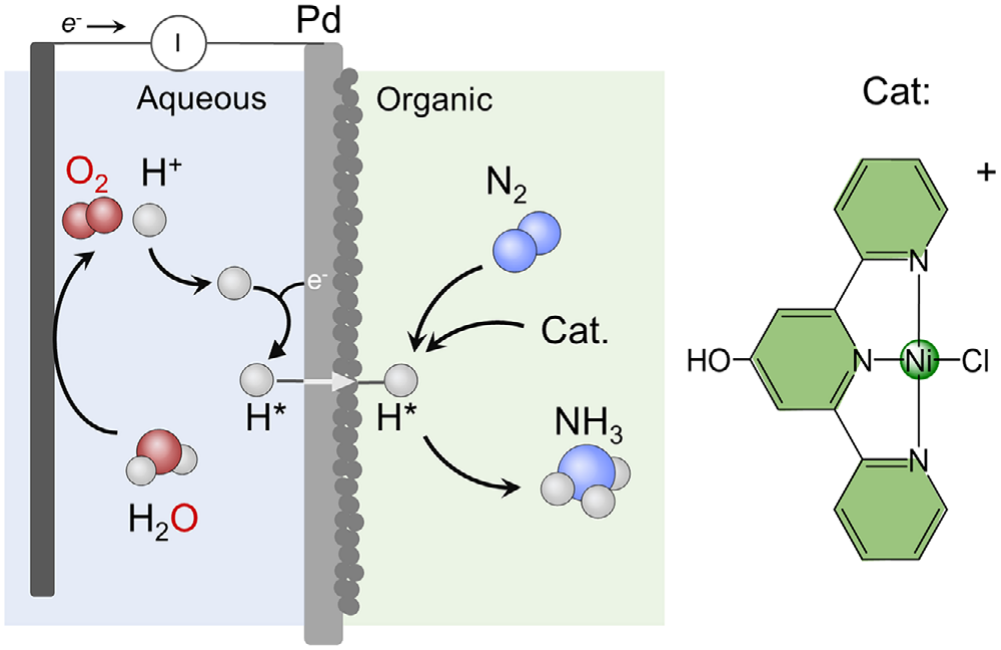
An illustration of a palladium membrane reactor (PMR) coupled with Nitpy‐based catalysts for the reduction of dinitrogen.

## 2 Results and Discussion

The mono‐terpyridine nickel (NitpyOH) was synthesized using previously established routes (Figure S1) [37, 38]. An excess of nickel salt and terpyridine ligand were refluxed at 90°C in a molar ratio of 1.5:1. Infrared spectroscopy (Figure S2a) and UV–vis spectroscopy were used to identify the successful formation of the catalyst. UV–vis spectra showed distinct absorption bands at λ = 280, 299, and 320 nm (Figure S2b). Characterization of all structural analogs of NitpyOH can be found in Figures S3–S16. Single‐crystal structural determination further showed that the mono‐terpyridine Ni catalysts were hexacoordinated with one terpyridine ligand and either three water ligands or two water ligands and one chloride ligand after recrystallization from the slow evaporation of water (Tables S2 and S3) [39].

### 2.1 Electrochemical Studies

The Pd membrane reactor in our work used a commercially purchased 25 µm Pd foil. Similar to previous works [32], the surface area of the side of the foil in contact with the organic solvent was increased through electrodeposition of Pd. This roughened side of the Pd foil was put in contact with NitpyOH in dimethylformamide (DMF) in an H‐cell set‐up (Figures S17–S19). Unless otherwise stated, electrochemical experiments were performed in triplicate using independently prepared reaction mixtures, and the results are reported as the mean ± standard deviation. Reproducibility was assessed by comparing the measured ammonia production and Faradaic efficiencies across independent experiments. DMF was selected as the solvent because NitpyOH showed complete dissolution at a concentration of 1 mM, whereas complete dissolution was not observed in ethanol, THF, DMSO, or iPrOH. In addition, DMF provides good chemical stability under the electrochemical conditions employed and a relatively wide electrochemical potential window [40, 41]. Notably, N_2_RR activity with NitpyOH was observed only when DMF was used among the five solvents investigated. Cyclic voltammetry of the Pd membrane with the roughened side exposed to air showed a pronounced dehydrogenation peak at 0.58 V vs. RHE (Figure 2b). Replacing the air interface with 1 mM NitpyOH in Ar‐saturated DMF resulted in a decrease in peak intensity and a positive shift to 0.64 V vs. RHE. A further positive shift to 0.67 V vs. RHE was observed for 1 mM NitpyOH in N_2_‐saturated DMF. The dehydrogenation feature remained positively shifted under N_2_ relative to Ar across scan rates of 5–20 mV s^−1^ (Figure S23), with the peak occurring at 0.67 V under N_2_ compared with 0.60–0.64 V under Ar. These changes are consistent with transfer of membrane‐derived hydrogen to NitpyOH and indicate that the presence of N_2_ further influences the hydrogen‐transfer equilibrium. In addition, the N_2_‐saturated solution exhibited a slightly more negative HER onset potential of −0.0308 V vs. RHE at a current density of −1 mA cm^−2^ and a lower cathodic current than the Ar‐saturated solution and the PMR exposed to air, which exhibited onset potentials of −0.0132 and +0.0127 V vs. RHE, respectively, at −1 mA cm^−2^. This suggests that a fraction of the active hydrogen is diverted away from hydrogen evolution in the presence of N_2_.

**FIGURE 2:**
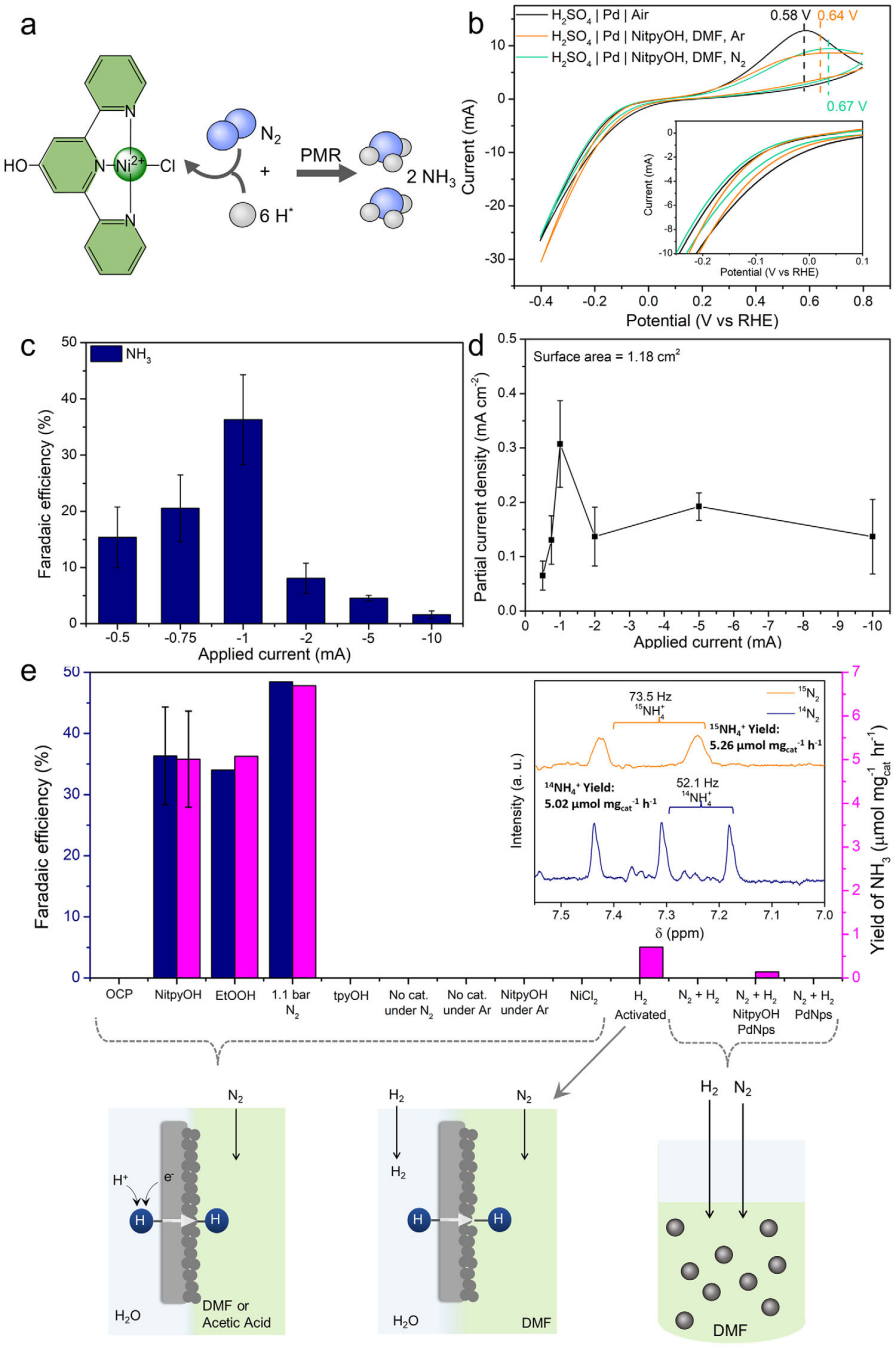
General reaction scheme for dinitrogen reduction to ammonia using NitpyOH as a catalyst (a), Cyclic voltammetry at 20 mV/s of the palladium membrane reactor exposed to air and 1 mM Ar or N_2_ sat. NitpyOH in DMF (b), FE for the production of ammonia from dinitrogen (c), partial current density for NH_3_ production from dinitrogen (d), Peak FE and NH_3_ production rate at an applied current density of −0.85 mA cm^−2^ compared to various control experiments. ^1^H NMR quantification of ^14^NH_4_^+^ and ^15^NH_4_^+^ formed via N_2_RR is shown in the inset graph. Additional conditions include PMR activation with H_2_ and reactions using Pd nanoparticles as a catalyst under N_2_/H_2_ purge (e). All measurements were performed in triplicate (or more), and error bars represent the standard deviation.

We first investigated dinitrogen reduction as a function of applied current density over a 5 h timeframe using an N_2_‐saturated DMF solvent. Ammonia was observed as the primary product with NMR spectroscopy. To further investigate the stability of ammonia production, time‐resolved electrolysis experiments were performed with ^14^N_2_ and ^15^N_2_ (Figure S24). The Faradaic efficiency remained relatively constant between 35% and 39% (Figure S24a), while the cumulative NH_3_ produced increased approximately linearly over 5 h (Figure S24b), demonstrating sustained ammonia formation and stable catalytic performance throughout electrolysis. Hydrazine, a potential intermediate and partially hydrogenated product, was not detected (Figures S25 and S26). H_2_ was produced as a secondary product, with its production rate increasing at larger applied currents (Figure S27). However, in the absence of the catalyst, an FE of 100% for H_2_ was observed (Figure S28). The FE for ammonia production reached a peak of 36% at an applied current density of −0.85 mA cm^−2^ (Figure 2c), generating an ammonia yield of 5.02 µmol mg_cat_^−1^ h^−1^. Similarly, the partial current density also followed the same trend (Figure 2d).

A strict protocol with requisite control experiments was followed to eliminate false positives in NH_3_ detection [42, 43]. As verification of N_2_ as the N‐source for ammonia, we performed electrolysis with isotopically labeled ^15^N_2_ as the reactant, which was also thoroughly cleaned of potential impurities using a dinitrogen gas purifier. Quantitative NMR analysis confirmed ^15^NH_4_^+^ production, corresponding to a Faradaic efficiency of 36% and ammonia yield of 5.26 µmol mg_cat_^−1^ h^−1^ at −0.85 mA cm^−2^, which is comparable to that obtained when using ^14^N_2_ as the N‐source. The ^14^NH_4_^+^ and ^15^NH_4_^+^ products were identified by their characteristic triplet (J = 52.1 Hz) and doublet (J = 73.5 Hz) resonances, respectively. A series of control experiments were conducted to verify that ammonia production required all key components of the system (Figure S21a). No detectable ammonia was observed in the presence of NitpyOH under Ar, in catalyst‐free experiments under either Ar or N_2_, with the free ligand (tpyOH) under N_2_, or with NiCl_2_ under N_2_. These results demonstrate that ammonia formation requires the simultaneous presence of N_2_, the Ni‐terpyridine catalyst, and membrane‐mediated hydrogen delivery. To exclude the possibility of DMF acting as a nitrogen source, acetic acid was employed as an alternative solvent. Under these conditions, ammonia production was still observed with Faradaic efficiencies comparable to those obtained in DMF, confirming that the detected ammonia does not originate from solvent decomposition.

The importance of electrochemically driven hydrogen delivery was further assessed by replacing the PMR with direct H_2_ bubbling into the aqueous compartment under open‐circuit conditions. Although ammonia was detected, the production rate was substantially lower than that observed during electrochemical operation (Figure S29), highlighting the role of the applied bias in enhancing trans‐membrane hydrogen transport. Finally, increasing the N_2_ pressure from 1.01 to 1.1 bar increased the Faradaic efficiency for NH_3_ production from 36% to 48% (Figure 3e), suggesting a positive reaction order with respect to N_2_ and indicating the potential for further improvements in performance at higher N_2_ pressures.

**FIGURE 3:**
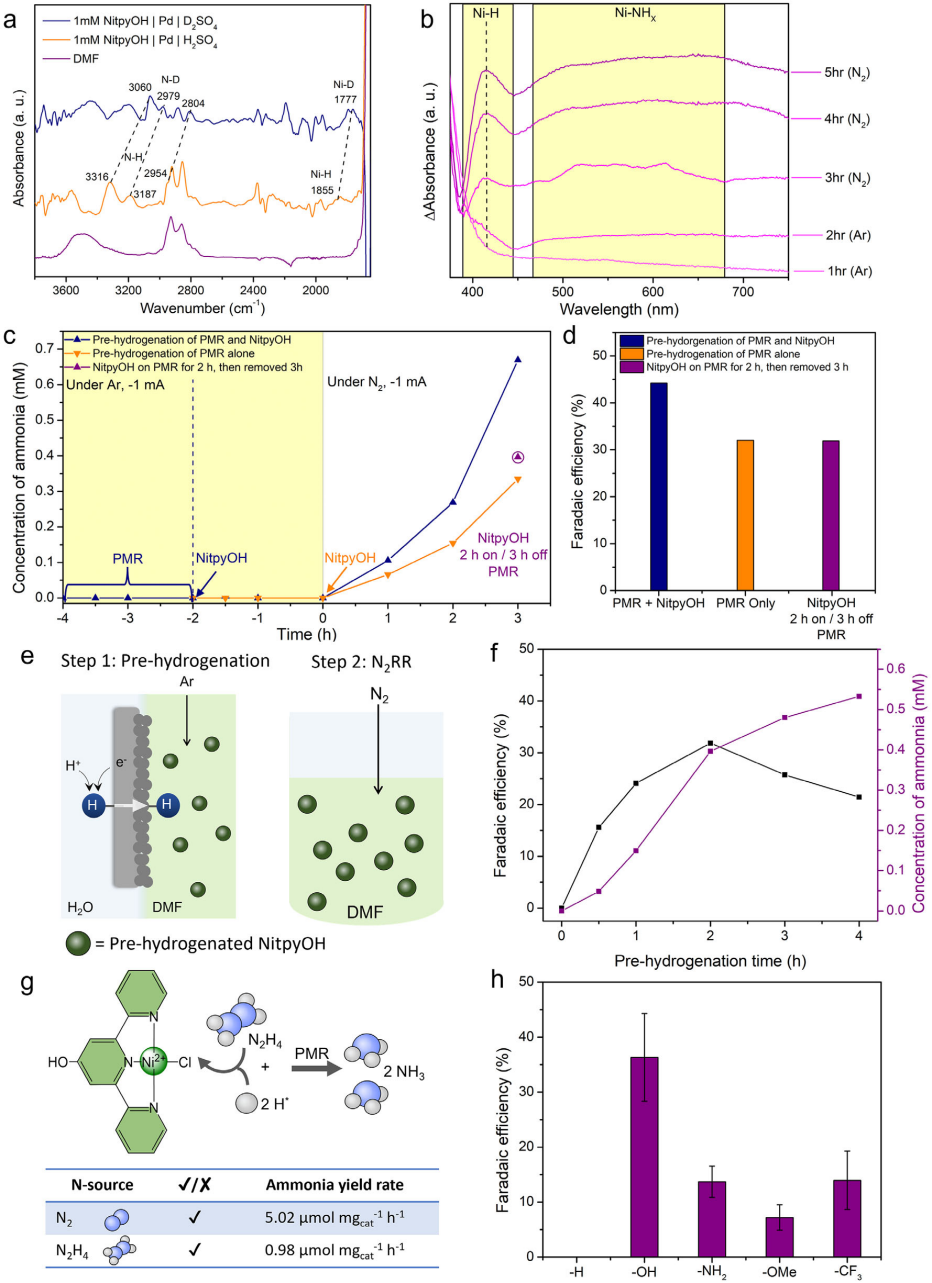
In situ FTIR spectroscopy showing the reaction intermediates detected using H_2_SO_4_ and D_2_SO_4_ as proton‐source (a), in situ UV–vis spectroscopy conducted initially under argon for 2 h, followed by exposure to N_2_ reactant for 3 h (b), Concentration of ammonia produced from various pre‐hydrogenation experiments, including: pre‐hydrogenation of both the PMR and NitpyOH (blue), pre‐hydrogenation of the PMR only (orange), and pre‐hydrogenation of NitpyOH for 2 h followed by removal from the PMR for 3 h (purple) (c), FE od ammonia at various pre‐hydrogenation conditions (d). N_2_RR performance following (e) electrochemical pre‐hydrogenation of NitpyOH under Ar for different durations, followed by removal of the catalyst solution from the palladium membrane reactor: (f) Faradaic efficiency for NH_3_ and NH_3_ concentration after 3 h of N_2_RR. Comparison of ammonia production rates using different nitrogen sources (g) and FEs of ammonia using various functionalized Nitpy catalysts (h). All measurements were performed in triplicate (or more), and error bars represent the standard deviation.

To further evaluate the intrinsic catalytic activity of the system, we conducted control experiments using Pd nanoparticles (Pd NPs), which can spontaneously split H_2_ to 2H^*^, and 1 mM NitpyOH in DMF under an N_2_/H_2_ atmosphere. This setup, conducted in the absence of electrochemical input, led to measurable ammonia production (Figure S30), albeit at significantly lower rates than the electrochemical system. These results suggest that the Pd NPs and NitpyOH are capable of chemically reducing N_2_ to NH_3_, likely via an H^*^‐driven pathway. The reduced activity relative to electrochemical conditions highlights the importance of applied current in enhancing either PCET or hydride generation at the Pd surface. In addition, without either the Pd NPs or NitpyOH, the system did not yield ammonia, confirming the need for both Pd and NitpyOH catalysts.

The stability of the standard system was evaluated through extended electrolysis for 11 days, and 12 h at −0.85 mA cm^−2^ applied current density. The reactor generated 0.49 mmol of NH_3_ with an overall FE of 28% and a catalyst turnover number (TON) of 16 (Figure S31). UV–vis and Raman spectroscopy (Figures S32 and S33) were performed after 5 h of electrolysis at −0.85 mA cm^−2^, showing no changes in the spectroscopic signature of the NitpyOH catalyst. XRD was also performed on the PMR, showing a diffraction pattern consistent with Pd nanoparticles, without any detectable deposition of Ni metal or oxide on the Pd surface (Figure S34). To further examine the possibility of Ni deposition onto the palladium membrane, post‐mortem XPS and ICP‐OES analyses were performed on a palladium membrane after 5 h of electrolysis at −0.85 mA cm^−2^ using 1 mM NitpyOH in N_2_‐saturated DMF. XPS (Figure S35a–c) showed no detectable Ni on the surface of the palladium membrane, with only Pd, C, N, and O being identified, consistent with the use of the membrane in an organic solvent such as DMF. ICP‐OES analysis (Figure S35d) detected no Ni above the method detection limit (0.1 mg L^−1^), with a measured Ni signal of 0.072 mg L^−1^ following digestion of the membrane in aqua regia. Furthermore, the membrane was reused after electrolysis with NitpyOH and subsequently tested in fresh DMF without catalyst, with no measurable ammonia production from dinitrogen (Figure S36). Together, these results provide no evidence for significant Ni deposition onto the Pd membrane and support the molecular NitpyOH complex as the primary catalytically active species.

### 2.2 Mechanistic Insights

To gain a deeper understanding of the mechanism, we utilized infrared and UV–vis spectroscopy to detect reaction intermediates en route to the production of ammonia. From in situ FTIR spectroscopy (Figure 3a), when using open‐circuit conditions as a background, several bands were identified at 1855, 2954, 3187, and 3316 cm^−1^ when a current density of −0.85 mA cm^−2^ was applied. Compared to previous literature reports, the band at 1855 cm^−1^ is tentatively assigned to an N─H vibration mode [23], with the other bands tentatively being assigned as N─H vibration modes. The identity of these bands is further supported by repeating the experiment using 0.5 M D_2_SO_4,_ in which the corresponding shift is observed for the N─H and Ni─H bands. Although these observations support the presence of hydrogen‐containing Ni and N species, they do not by themselves establish a unique molecular structure for these intermediates. Upon further examination of the lower‐wavenumber region of the FTIR spectra (Figure S37), an additional band at 1150 cm^−1^ was identified under both H‐ and D‐containing conditions. This is tentatively assigned to the N─N vibrational mode [44] and shows no shift upon H/D substitution, which is consistent with predominantly N─N character. Together with the isotope‐sensitive N─H bands, this observation provides further evidence for hydrogenated N─N species under the N_2_RR conditions.

To complement our IR experiments, we turned to in situ UV–vis spectroscopy (Figure 3b). In the UV–vis experiments, the membrane reactor containing NitpyOH was pre‐hydrogenated for 2 h under argon, followed by exposure to a dinitrogen atmosphere for 3 h. During the reaction, two key bands were identified: a distinct band at approximately 410 nm and broader bands between 475 and 650 nm. A control UV–vis experiment was conducted under argon for 5 h (Figure S38), and the spectra there showed a gradual increase in the intensity of the only 410 nm band; thus, it is associated with the formation of an electrochemically generated hydrogenated state of NitpyOH. The broader bands between 475 and 650 nm are tentatively attributed to Ni‐NH_x_‐containing species. As a reference, [Ni(NH_3_)_6_]Cl_2_ in DMF (Figure S39) exhibited a broad visible absorption between 500 and 700 nm, providing qualitative support for a contribution from Ni‐NH_x_ coordination, although this comparison does not establish the identity of a specific intermediate.

An attempt was also made to identify whether the mechanism had a bimolecular step during the reaction. A series of concentrations of the NitpyOH catalyst were integrated into the palladium membrane reactor with an applied current density of −0.85 mA cm^−2^ (Figure S40). No ammonia formation was observed at concentrations of 0.5 and 0.75 mM, with ammonia production beginning at 0.9 mM, reaching a maximum FE at 1 mM. At concentrations above 1 mM, however, the FE decreased once again. These results are consistent with the possibility of a bimolecular mechanism, potentially involving interactions between hydrogenated and nitrogen‐bound catalyst species. At minimal catalyst concentrations, no product formation may occur due to the insufficient number of active species. Conversely, at higher concentrations, dead‐end pathways, such as the formation of Ni‐N_2_‐Ni dimers, could dominate, thereby reducing the overall efficiency.

We next conducted two different pre‐hydrogenation experiments (Figure 3c,d): one in which only the membrane was pre‐hydrogenated under argon for 2 h, and another in which the membrane was pre‐hydrogenated for 2 h, followed by the addition of NitpyOH and further exposure under argon for an additional 2 h. These experiments demonstrated an increase in ammonia production when both the membrane and NitpyOH were pre‐hydrogenated. Furthermore, a corresponding increase in FE for ammonia production was observed, despite a higher overall charge passed (Figure 3d). A third experiment was conducted in which NitpyOH was pre‐hydrogenated on the Pd membrane reactor under an argon atmosphere for 2 h. Following this step, the DMF solution containing the catalyst was removed from the reactor, and the system was subsequently purged with N_2_ for 3 h prior to further analysis. Notably, ammonia was detected under these conditions at higher rates than when only the Pd membrane was pre‐hydrogenated. Additionally, the FE was comparable to that observed with pre‐hydrogenation of the PMR alone. These findings indicate that formation of a hydrogenated catalyst state prior to N_2_ exposure plays an important role in N_2_ reduction.

To further investigate the influence of the catalyst hydrogenation state, we subsequently varied the duration of electrochemical pre‐hydrogenation of NitpyOH under Ar prior to removing the catalyst solution and performing N_2_RR under otherwise identical conditions (Figure 3e,f). Increasing the pre‐hydrogenation time from 0 to 2 h resulted in a progressive increase in ammonia production and Faradaic efficiency, with the maximum FE being observed after 2 h of pre‐hydrogenation. However, further increasing the pre‐hydrogenation time to 3 and 4 h resulted in a gradual decrease in FE. Although the cumulative ammonia concentration continued to increase with longer pre‐hydrogenation times, the rate of ammonia accumulation decreased substantially beyond 2 h. This behavior indicates that increasing the extent of catalyst hydrogenation does not continuously enhance N_2_RR activity. The initial increase in activity is consistent with formation of a productive hydrogenated catalyst state, whereas prolonged pre‐hydrogenation may increasingly favor competing HER and/or the formation of hydrogenated species that enter less productive or catalytically inactive pathways. Together, these results provide functional evidence that the hydrogenation state of NitpyOH influences N_2_RR activity.

With this result, we wanted to probe the role of the hydrogenated species in the catalytic mechanism by introducing 0.1, 0.5 and 1 mM concentrations of base (K_2_CO_3_) or acid (H_2_SO_4_) to the system during electrolysis at −0.85 mA cm^−2^ (Figures S41 and S42). For H_2_SO_4_, these concentrations correspond to nominal proton equivalents of 0.2, 1.0 and 2.0 µmol, respectively, based on the 1 mL electrolyte volume. The addition of K_2_CO_3_ led to an exponential decrease in FE for ammonia production. Conversely, the addition of H_2_SO_4_ resulted in a gradual decline in FE. These effects occurred without any noticeable signs of catalyst degradation. The strong sensitivity to basic conditions and modest response to acid addition provide evidence that catalyst pre‐hydrogenation is crucial to the N_2_ reduction pathway. The observed decrease in Faradaic efficiency upon addition of base is consistent with suppression of catalyst pre‐hydrogenation, thereby inhibiting the initial hydrogenation step required for N_2_ activation. In contrast, while the addition of small amounts of acid might be expected to promote catalyst pre‐hydrogenation, the lack of a sustained increase in Faradaic efficiency and the subsequent decline at higher acid concentrations suggest that excess protons promote competing pathways, most notably hydrogen evolution, or lead to non‐productive protonation events at the catalyst. These results indicate that efficient catalysis requires a finely balanced proton environment, where sufficient proton availability enables catalyst pre‐hydrogenation without favoring competitive side reactions.

In a final attempt to further identify potential intermediates, we turned to the use of hydrazine, a potential intermediate, as a reactant instead of N_2_ (Figure 3g). Ammonia was generated with an FE of 2.4% and an ammonia yield rate of 0.98 µmol mg_cat_^−1^ h^−1^ at an applied current density of −0.85 mA cm^−2^. Even though the FE and production rate are significantly lower compared to N_2_RR, the formation of NH_3_ demonstrates that N_2_H_4_ is a chemically competent substrate under the reaction conditions and is consistent with its potential involvement as an intermediate. The lower NH_3_ FE observed when using N_2_H_4_ as the reactant may in part result from the additional water introduced with the aqueous N_2_H_4_ stock solution, which substantially alters the reaction conditions. As a control, N_2_RR was performed with the same amount of water introduced by the N_2_H_4_ stock solution (67 mM H_2_O), resulting in a decrease in ammonia Faradaic efficiency to 10%, compared with 36% under the standard N_2_RR conditions, at an applied current density of −0.85 mA cm^−2^ for 5 h.

A series of structure‐activity relationships (Figure 3h) were next investigated to probe the influence of ligand substitution on N_2_ reduction activity. The non‐functionalized terpyridine ligand showed no detectable ammonia formation under an applied current density of −0.85 mA cm^−2^ in the PMR, indicating that substitution at the 4′‐position of the central pyridine ring is required to enable catalytic activity under these conditions.

Introduction of functional groups at this position led to measurable ammonia formation, with the OH‐functionalized complex showing the highest observed performance. The NH_2_ derivative exhibited lower activity, reaching a peak Faradaic efficiency (FE) of 13.7%. These differences may reflect variations in hydrogen‐bonding and proton‐transfer capability between the OH and NH_2_ substituents, as well as their distinct acid‐base properties under the reaction conditions. The ability of 4′‐amino‐ and 4′‐hydroxy‐functionalized terpyridines to participate in hydrogen‐bonding interactions has been demonstrated experimentally [45, 46, 47]. To further probe electronic effects, electron‐donating (OMe) and electron‐withdrawing (CF_3_) substituents were introduced. The OMe‐functionalized complex gave a reduced FE of 7.2%, while the CF_3_ derivative showed improved performance (FE = 13.9%) relative to OMe. Notably, the FE values obtained for the CF_3_ and NH_2_ derivatives were 13.95% ± 5.33% and 13.70% ± 2.82%, respectively, indicating that their difference is within the experimental uncertainty.

To further investigate the electronic origin of these differences, DFT calculations were performed for the substituted complexes (Table S1 and Figures S43–S47). The calculated HOMO and LUMO energies and Hirshfeld charges at the Ni center demonstrate that the substituents systematically modify the electronic structure of the complexes. However, no simple relationship between these electronic descriptors and N_2_RR activity was observed. In particular, the CF_3_ and NH_2_ derivatives exhibit substantially different Ni‐centered electron densities, while showing essentially identical experimental FE. This indicates that the catalytic activity cannot be attributed solely to the electronic properties of the Ni center. The OH‐, NH_2_‐, and OMe‐functionalized complexes also exhibit similar HOMO‐LUMO gaps (Figure S48) and relatively similar Ni‐centered electron densities despite their different catalytic activities, suggesting that additional factors contribute to the observed performance.

The combined trends instead suggest that both electronic effects and secondary‐sphere interactions contribute to the observed differences in catalytic performance. In contrast to OMe, the OH and NH_2_ substituents provide O─H and N─H functionalities capable of participating in hydrogen‐bonding and proton‐transfer interactions. More broadly, secondary‐sphere hydrogen‐bonding interactions have been shown to influence molecular electrocatalysis, with phenolic substitution reported to enhance CO_2_ reduction through local proton‐transfer and hydrogen‐bonding interactions [48, 49]. Reported pK_a_ values for analogous hydroxyl‐ and amino‐functionalized pyridines are 11.09 [50] and 8.89 [51], respectively, providing a qualitative indication of their distinct acid‐base properties, although these values cannot be directly assigned to the corresponding terpyridine ligands. The calculated electrostatic potential (ESP) surfaces further demonstrate that ligand substitution alters not only the electronic environment at the Ni center but also the spatial distribution of electrostatic potential across the ligand framework (Figure S49). In particular, the OH‐ and NH_2_‐functionalized complexes exhibit a less positive electrostatic environment across the ligand framework compared with the H‐, OMe‐, and CF_3_‐functionalized complexes, with distinct local electrostatic features associated with the O and N atoms, respectively. These differences provide a further indication that the substituents can modulate the secondary coordination sphere in addition to their influence on the electronic structure of the Ni center. Overall, these results suggest that the substituent‐dependent N_2_RR activity arises from the combined influence of electronic effects and secondary‐sphere interactions, rather than from a single electronic descriptor.

Based on our experimental observations, we propose that ammonia formation proceeds through an alternating pathway. In this mechanism, the first key step involves initial hydrogenation of the NitpyOH catalyst to generate a Ni‐H species capable of binding N_2_. Once coordinated, subsequent hydrogen species are supplied through either additional Pd‐H transfer or from solution‐based Ni‐H species, which sequentially deliver hydrogen to the Ni‐N_2_ adduct. This hydride‐driven activation sequence is consistent with our pre‐hydrogenation studies, acid/base experiments, and in situ spectroscopy, collectively supporting a mechanism in which membrane‐derived hydride delivery enables stepwise N─H bond formation along the alternating pathway (Figure 4).

**FIGURE 4:**
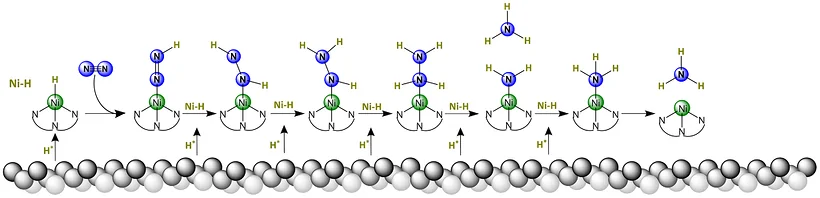
The proposed reaction mechanism for the synthesis of ammonia from dinitrogen reduction using a Pd membrane reactor.

To further elucidate the reaction mechanism, we performed density functional theory (DFT) computations. We first investigated the adsorption of H on the NitpyOH catalyst. A strong adsorption energy of −1.9 eV was obtained, with a calculated Ni─H bond distance of 1.58 Å (Figure 5a‐i). To investigate the role of the pre‐hydrogenated catalyst in N_2_ activation, N_2_ was introduced to the Ni‐H intermediate. The calculations suggest that hydrogen transfer from Ni‐H to N_2_ can facilitate N_2_ binding at the Ni center, forming the ^*^N‐NH species (Figure 5a‐iii), corresponding to the first hydrogenation step in the proposed N_2_RR pathway. This observation is consistent with the experimentally observed increase in NH_3_ formation following pre‐hydrogenation of NitpyOH in the absence of the PMR. The reaction energy associated with H transfer from Ni‐H to adsorbed ^*^N_2_ was calculated to be 1.07 eV. Notably, this hydride‐to‐N_2_ transfer parallels mechanistic motifs reported in dinickel systems, where H_2_ elimination from a Ni‐H platform enables N_2_ binding in a bent bridging radical‐anion mode [52, 53].

**FIGURE 5:**
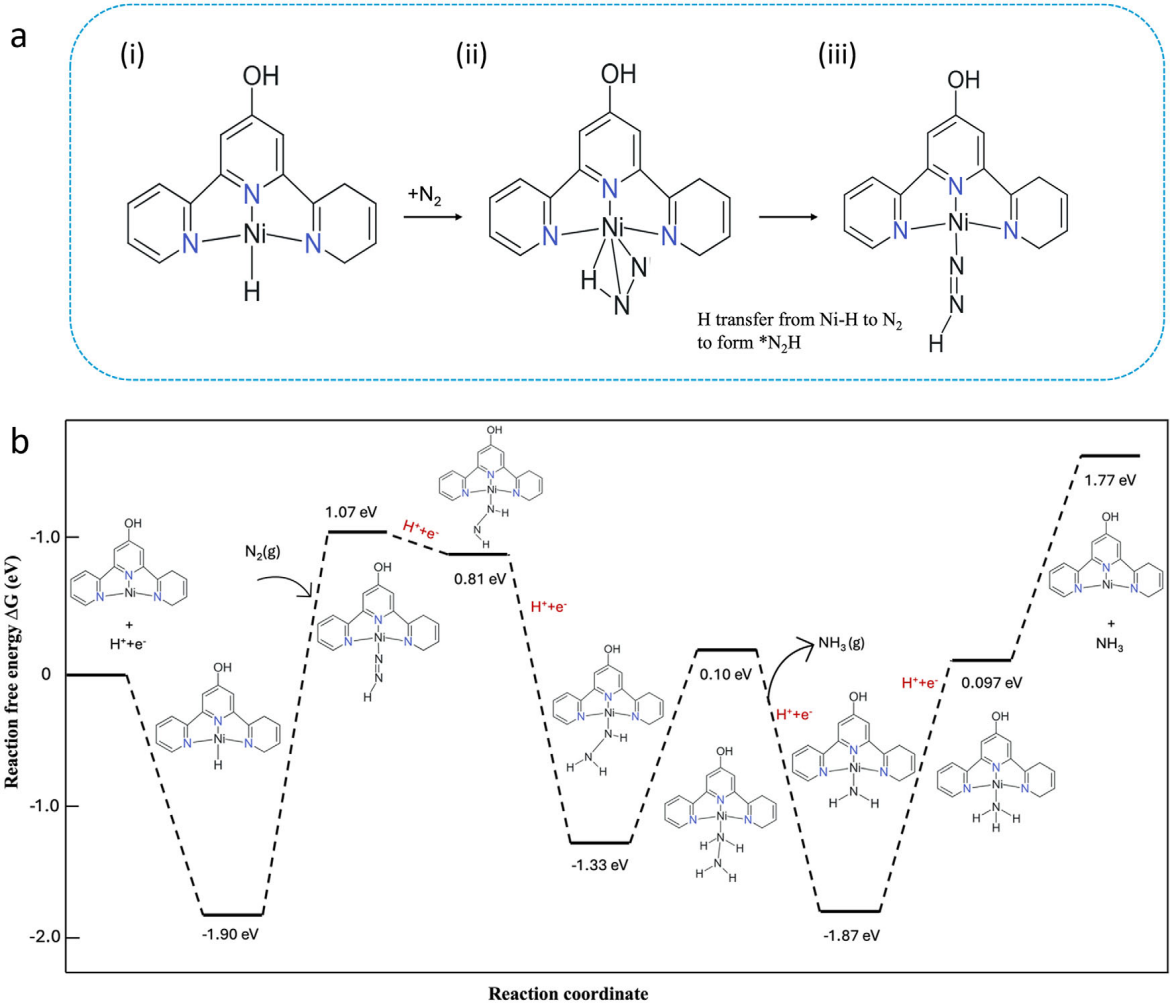
Mechanism of proton transfer from Ni‐H to N_2_ for the formation of N_2_RR intermediates (a). Reaction free energy diagram of the electrocatalytic N_2_RR over pre‐hydrogenated NitpyOH catalyst obtained from DFT calculations (b).

This step corresponds to the largest thermodynamic uphill free‐energy change along the computed pathway (∼2.9 eV), identifying it as the most energetically demanding transformation in the proposed mechanism. The successive protonation steps generate ^*^NH‐NH, ^*^NH‐NH_2_, ^*^NH_2_‐NH_2_, ^*^NH_2_, and ^*^NH_3_ intermediates. Figure 5b depicts the corresponding reaction free‐energy diagram for N_2_RR over the NitpyOH catalyst. The formation of ^*^NH‐NH_2_ was calculated to be energetically favorable (ΔG = −1.33 eV), followed by the formation of ^*^NH_2_‐NH_2_, which is slightly uphill in energy (ΔG = 0.10 eV). In the subsequent step, ^*^NH_2_ is generated through the release of one NH_3_ molecule with a ΔG of −1.87 eV. Further protonation yields ^*^NH_3_ adsorbed to the catalyst with a calculated ΔG of 0.097 eV.

In addition, redox potentials derived from the computed reaction energies for the N_2_RR intermediates are provided in Table S6. The ^*^NH_2_ intermediate exhibited the most negative calculated redox potential (−2.5 V) among the species considered. These calculated values should be interpreted cautiously, as they are derived from the computed reaction free energies and do not explicitly account for the influence of electrochemical polarization, applied current, or hydrogen transport through the Pd membrane. Furthermore, we analyzed the electronic configuration of the Ni center for each reaction intermediate. For most intermediates (^*^N_2_, ^*^N‐NH, ^*^NH‐NH, ^*^NH_2_‐NH_2_, and ^*^NH_3_), the Ni center was predicted to exhibit an electronic configuration close to 3d^8.5^, corresponding to an average oxidation state of approximately +1.5. This behavior suggests electronic charge redistribution between the Ni center and adsorbed N_2_RR intermediates during the proton‐coupled electron‐transfer process.

## 3 Concluding Remarks

In this work, we have established a molecular catalyst‐integrated palladium membrane reactor as a unique platform for N_2_ activation. Using a mono‐terpyridine nickel complex (NitpyOH), we achieve Faradaic efficiencies of up to 36%. Beyond catalytic performance, our combined in situ spectroscopy, electroanalytical studies, and complementary computational analysis reveal that hydride delivery from the Pd membrane generates a Ni‐H intermediate that is central to the activation of dinitrogen. These findings highlight a unique mode of molecular catalyst activation in which a hydrogen‐permeable membrane serves as a controllable hydride source, opening a new strategy for accessing key intermediates inaccessible through conventional electrochemical or chemical routes. The integration of molecular design with membrane‐mediated hydride delivery provides a modular platform for advancing nitrogen fixation chemistry and suggests broader applicability to molecular electrocatalysis where selective hydride transfer under mild conditions is desired.

## Author Contributions

**Morgan Mckee**: investigation, writing – original draft, writing – review and editing. **Sai Phani Kumar Vangala**: investigation. **Farzaneh Farzinpour**: investigation. **Jasmin Wagner**: investigation. **Giacomo Pugliese**: investigation. **Hamed Heidarpour**: investigation. **Hossein Bemana**: investigation. **J. Felipe Estrada‐ruiz**: investigation. **Jörg Daniels**: investigation. **Alessandro Bismuto**: investigation, supervision. **Ali Seifitokaldani**: investigation, supervision. **Nikolay Kornienko**: conceptualization, supervision.

## Conflicts of Interest

The authors declare no conflicts of interests.

## Supporting information




**Supporting File**: advs78155‐sup‐0001‐SuppMat.pdf.

## Data Availability

All data will be available upon request to the corresponding author via email for non‐commercial purposes. Data will be saved for 10 years, and requests will be responded to within 10 working days.
