# Supplementary material for: Molecular Electrocatalysis Coupled With a Palladium Membrane Reactor Affords Selective Nitrogen Reduction
Source: Adv Sci (Weinh). 2026 Oct 6:e78155. Online ahead of print. doi: 10.1002/advs.78155 (PMC13643232; doi:10.1002/advs.78155)
Supplement: Supplementary file 1 [file ADVS-9999-e78155-s001.pdf]

Molecular electrocatalyst coupled with a palladium membrane reactor affords selective nitrogen reduction

Morgan McKee[1], Sai Phani Kumar Vangala[2,3], Farzaneh Farzinpour[1], Jasmin Wagner[1], Giacomo Pugliese[1], Hamed Heidarpour[2], Hossein Bemana[1], J. Felipe Estrada-Ruiz[1], Jörg Daniels[1], Alessandro Bismuto[1], Ali Seifitokaldani[2]* and Nikolay Kornienko[1]*

[1]Institute of Inorganic Chemistry, University of Bonn, Gerhard-Domagk-Str. 1, 53121 Bonn, Germany

[2]Department of Chemical Engineering, McGill University, 3610 University Street, Montréal, H3A 0C5 Québec, Canada

[3]Department of Chemical Engineering, Indian Institute of Science Education and Research (IISER) Bhopal, Bhopal, 462066 Madhya Pradesh, India

*Correspondence to: ali.seifitokaldani@mcgill.ca, nkornien@uni-bonn.de

Experimental procedures

Synthesis of nickel complexes

The nickel-terpyridine complexes (non-functionalized, hydroxy, amino, methoxy, trifluoromethyl) were prepared following modified literature procedures.[1,2] An excess of $NiCl_2$ (193.5 mg, 1.5 mmol) was dissolved in ethanol and stirred at room temperature. While stirring, the corresponding terpyridine ligand (1 mM) was dissolved in ethanol and then added to the $NiCl_2$ solution. After the addition of the tpyR ligand, the solution was heated to 90 ˚C and refluxed for 9 hours until a blue-green precipitate formed at varying yields of around 50-55%.

[Ni(tpyOH)(H_2O)$_2$Cl]Cl·2H_2O:

^{1}H NMR (500 MHz, D_2O, 298 K) δ [ppm] = 71.07, 64.09, 39.56, 13.08, 3.62, 1.14

HRMS (ESI(+)): m/z: 341.99

Elemental analysis calculated for [Ni($C_{15}H_{11}ON_3$)(H_2O)$_3$]Cl_2 (element, %): C, 41.62; H, 3.96; N, 9.71; obtained: C, 41.47; H, 3.84; N, 9.67.

[Ni(tpy)(H_2O)$_2$Cl]Cl·H_2O:

^{1}H NMR (500 MHz, D_2O, 298 K) δ [ppm] = 73.12, 64.79, 39.60, 15.93, 13.14, 1.23

HRMS (ESI(+)): m/z: 325.998

Elemental analysis calculated for $[Ni(C_{15}H_{11}N_3)(H_2O)_2Cl]Cl.1.5H_2O$ (element, %): C, 42.3; H, 4.26; N, 9.87; obtained: C, 42.06; H, 4.00; N, 9.78.

$[Ni(tpyNH_2)(H_2O)_2Cl]Cl{\cdot}2H_2O$:

^{1}H NMR (500 MHz, D_2O, 298 K) δ [ppm] = 72.67, 63.77, 39.91, 13.06, 3.57, 1.10

HRMS (ESI(+)): m/z: 341.0095

Elemental analysis calculated for $[Ni(C_{15}H_{12}N_4)(H_2O)_2Cl]Cl.2H_2O$ (element, %): C, 40.04; H, 4.48; N, 12.45; obtained: C, 39.95; H, 4.46; N, 12.31.

$[Ni(tpyOMe)(H_2O)_2Cl]Cl{\cdot}2H_2O$:

^{1}H NMR (500 MHz, D_2O, 298 K) δ [ppm] = 70.28, 64.62, 39.44, 13.07, 4.27

HRMS (ESI(+)): 356.0087

$[Ni(tpyCF_3)(H_2O)_2Cl]Cl{\cdot}H_2O$:

^{1}H NMR (500 MHz, D_2O, 298 K) δ [ppm] = 68.63, 65.64, 39.18, 13.16

^{19}F NMR (500 MHz, D_2O, 298 K) δ [ppm] = -71.61

HRMS (ESI(+)): m/z: 393.9869

Preparation of palladium membrane

Palladium foil (Pd, 0.025 mm thick, 99.95%) was roughed on one side by an electrodeposition method. This was achieved by placing the Pd foil in a H-cell set-up as the working electrode using a 15.9 mM $PdCl_2$ electrolyte in 0.5 M HCl. Graphitic carbon was used as the counter electrode and Ag/AgCl as the reference electrode. To produce a roughened Pd surface, a -0.2 V vs Ag/AgCl potential was applied until 13 C of charge (6.5 C cm^{-2}) was passed with an estimated total of 7.17 mg of palladium being deposited on the surface with a typical time of 90 min to achieve this. Ohmic losses were corrected using electrochemical impedance measurements prior to electrochemical measurement through the ZIR function within the EC-lab software.

Characterization

Electrochemistry and product quantification

Cyclic voltammetry (CV) was accomplished using a Bio-Logic SP-200 Potentiostat (BioLogic Science Instruments, France). A H-type two-compartment system was employed using the roughened Pd foil as the working electrode, graphitic carbon as the counter electrode, and Ag/AgCl as a reference electrode. The CVs were measured in the range of 0 ~ –2.0 V (vs. Ag/AgCl) at a sweep rate of 20 mV s^{-1}. Electrochemistry experiments were carried out in an H-type two-compartment glass electrochemical

cell. A Pd foil was pressed between the electrochemical compartments of the cell. The aqueous compartment contained 50 mL of 0.5 M H_2SO_4 while the organic compartment contained 1 mL of DMF with various concentrations of catalyst. Graphitic carbon was used as counter electrode, Ag/AgCl was used as reference, and the electrodeposited Pd membrane as working electrode. Ohmic losses were corrected using electrochemical impedance measurements prior to electrochemical measurement through the ZIR function within the EC-lab software. The catalyst solution in DMF was purged with N_2 for 30 min to saturate the solution or with Ar to remove any reactant and then chronopotentiometry was conducted at various currents. For isotope experiments, $^{15}N_2$ was purified using a nitrogen gas purifier connected directly to the $^{15}N_2$ gas bottle. Before use, the purifier was purged with argon to eliminate any potential impurities or other gas reactants inside the purifier. Additionally, an attempt was made to perform the reaction with nitrate. However, due to nitrate being insoluble in DMF, the reaction could not be done eliminating nitrate as a potential contaminant. Pre-hydrogenation experiments were carried out in two ways. In the first, only the membrane was pre-hydrogenated under argon for 2 h, after which 1 mM of NitpyOH was added and the system was purged with N_2 for 3 h. In the second experiment, the membrane was also pre-hydrogenated under argon for 2 h, followed by the addition of 1 mM NitpyOH. However, in this case, the system was left under argon for an additional two hours before switching to N_2 for a three-hour purge. NMR samples were taken every 30–60 min to monitor ammonia production.

Unless otherwise stated, electrochemical experiments were performed in triplicate using independently conducted electrolysis experiments under identical experimental conditions. The reported Faradaic efficiencies, ammonia production rates and related electrochemical data represent the mean ± standard deviation of these measurements. To ensure reproducibility of the PMR between experiments, the Pd membrane was cleaned after each electrochemical experiment by rinsing three times with deionized water followed by three rinses with ethanol, and subsequently dried at 100 °C in an oven prior to reuse.

In order to quantify the products of the reaction, NMR (Bruker AVANCE II 400 se) and gas chromatography were performed to reveal the content and composition of the gas and liquid products, respectively. For NMR analysis, 300 µL liquid electrolyte after an electrolysis run were mixed with 300 µL DMSO and 100 µL 0.5M H_2SO_4 to quantify liquid products. For NMR measurements, products were quantified using maleic acid as an internal standard and calibration curves for products in the liquid phase (Fig. S20). Typically, liquid products were acquired after 3-5 h of electrolysis. Gaseous products were similarly quantified through the integration of peak area corresponding to the products, which were first measured with a series of calibration curves (H_2). The GC was calibrated using calibration gas (Air Liquide, 1% H_2) and diluted with Ar gas to different ratios using a mass flow controller. The GC measurements were also collected via injection method with N_2 as the carrier gas in the GC.

Purification and impurity control of N_2

Both $^{14}N_2$ (5.0 grade) and $^{15}N_2$ gases were purified prior to use using a commercial inline N_2 gas purifier installed directly downstream of the gas cylinder. The purifier is designed to remove trace nitrogen-containing contaminants, including NH_3 and NO_x species, thereby minimizing the contribution of adventitious nitrogen sources during electrochemical measurements.

The effectiveness of gas purification was verified through a series of analytical control experiments. Blank electrolysis experiments were conducted under identical electrochemical conditions, but in the absence of the NitpyOH catalyst, yielding no detectable NH_4^+ by ^1H NMR spectroscopy (Fig. S21). In addition, purified N_2 gas was bubbled through the electrolyte under open-circuit conditions, followed by quantitative ^1H NMR analysis, which again showed no detectable NH_4^+. These controls establish that any residual nitrogenous impurities present after purification contribute below the detection limit of the NMR method and cannot account for the ammonia produced under catalytic conditions.

Isotopic labeling experiments further confirmed the origin of ammonia. When the reaction was conducted under a $^{15}N_2$ atmosphere, only $^{15}NH_4^+$ signals were observed by ^1H NMR, with no detectable $^{14}NH_4^+$ present. In addition, the amount of $^{15}NH_4^+$ produced was comparable to amount of $^{14}NH_4^+$ produced under standard reaction conditions. This excludes contributions from residual ^{14}N-containing impurities such as NO_x species and confirms that the observed ammonia arises from the reduction of dinitrogen.

To quantify potential NO_x impurities in the feed gases, compressed laboratory air, 5.0 grade N_2, and purified 5.0 grade N_2 were passed through an alkaline trap containing 0.1 M KOH (10 mL). Gas purging was conducted at a flow rate of 10 mL min^{-1} for 3 h, corresponding to a total gas volume of 1.8 L per experiment. A sealed alkaline trap maintained under identical conditions without gas flow was analyzed in parallel as a blank control.

Following gas exposure, aliquots of the alkaline trap solutions were collected for analysis. To enable quantification of total NO_x species ($NO_2^- + NO_3^-$), nitrate present in the trap solutions was first reduced to nitrite using Zn powder under acidic conditions. Briefly, the aliquots were acidified to pH 1-2 using HCl, followed by the addition of Zn powder and were stirred at room temperature for 60 min. After removal of excess Zn, the resulting solutions were analyzed using a Griess colorimetric assay.

Nitrite concentrations were determined by UV–Vis spectroscopy after the sequential addition of Griess reagent, with absorbance measured at 529 nm (Fig. S21). Quantification was performed using an independently prepared nitrite calibration curve. All measurements were corrected using the sealed trap blank.

Nitrite quantification was performed using a colorimetric method with Griess reagent. In this method, 200 µL of the electrolyte were mixed with 1.8 mL of deionized water and 2 mL of Griess reagent. The

resulting solution was stored in the dark for 30 min before recording the UV–Vis spectrum. The nitrite concentration was determined using a calibration curve (Fig. S20c,d), based on the absorbance at 529 nm.

Faradaic efficiency

The Faradaic efficiency (FE) was calculated by using the following formula:

$$FE = \frac{nZF}{Q}$$

where n is the moles of product, z is the number of electrons exchanged, F is Faraday's constant (96485 C mol^{-1}), and Q the total charge passed during the reaction.

Detection of hydrazine

Hydrazine was detected using the calorimetric method developed by Watt and Chrisp. Firstly, a solution of *p*-dimethylaminobenzaldehyde (1.198 g) in HCl (6 mL) and ethanol (60 mL) was prepared. Then, 1 mL of electrolyte was mixed with 1 mL of chromogenic reagent and left at room temperature for 20 min before recording the UV-Vis spectrum. A calibration curve (Fig. S24) using known concentrations of hydrazine was used to determine the amount of hydrazine produced using the absorption intensity at 454 nm.

In-situ infrared (IR) spectroscopy

IR spectra were acquired on a ThermoFischer Nicolet 380 FTIR-ATR with a ZnSe ATR crystal that was coated with a diamond surface. Typically, 800 scans were acquired for each measurement. A three-electrode cell was used for the in-situ IR experiment. Cu wire was used as counter electrode, Ag/AgCl was used as reference, the electrodeposited Pd membrane as working electrode. The experiments were carried out in a custom made spectroelectrochemical cell with the Pd membrane separating the H_2SO_4 and catalyst (organic) compartments.

Raman spectroscopy

Raman spectra were collected using a Renishaw Invia system with a 514 nm laser having a 5 mW output power. The laser line focus illumination technique was used that spread the laser intensity out over a line and minimized the power concentrated at any one spot. The spectra were collected at full intensity power, and a typical collection time was 60 s. For the sample a 1 mM NitpyOH solution was prepared in DMF and drop casted on to Ti metal before or after catalysis and the solvent was then evaporated.

UV-Vis spectroscopy

UV-Vis spectra were collected on an Agilent Cary 60 UV-Vis spectrophotometer, and samples were recorded before and after 5 h of electrolysis. For operando studies, a custom-made cell was used with

spectra being taken every minute over the span of 3 h. To assess the visible region, the pre-catalysis spectra was then subtracted from each spectrum to observe the change in absorbance.

X-ray photoelectron spectroscopy (XPS)

XPS measurements were performed using a Thermo Scientific K-Alpha X-ray Photoelectron Spectrometer equipped with a monochromatic Al Kα X-ray source. A spot size of 400 μm was used, with a constant analyser energy (CAE) pass energy of 50.0 eV. The Pd membrane was analysed following electrolysis under N_2RR conditions using 1 mM NitpyOH in DMF at -1 mA for 5 h. The membrane was subsequently rinsed and dried prior to XPS analysis. Survey and high-resolution spectra were collected to evaluate the elemental composition and chemical states of the membrane surface.

Inductively coupled plasma optical emission spectroscopy (ICP-OES)

ICP-OES measurements were carried out on a PerkinElmer Optima 8300 Spectrometer containing a Meinhard atomizer and a glass cyclone chamber. The spectrometer was calibrated using a blank solution of the Merck Certipur IV standard with concentrations of 0mg/L, 1 mg/L, 5 mg/L, and 10 mg/L. The measurement method involved three repetitive measurements for Ni 221 ax with integration times of 10 seconds.

Following electrolysis, the Pd membrane was dissolved in freshly prepared aqua regia ($HCl:HNO_3$, 3:1, v/v). Complete dissolution of the Pd membrane was observed. The resulting solution was subsequently diluted with MilliQ water to an appropriate concentration for ICP-OES analysis.

DFT calculations for reaction energetics

Molecular DFT: The molecular density functional theory (DFT) calculations were employed in this work to elucidate the mechanism of dinitrogen reduction reaction (N_2RR) over Nitpy molecular catalysts. The structural relaxation of the molecular catalysts was performed under the hybrid meta GGA functional, *M062X*[3] implemented in Gaussian 16 package[4] along with general basis sets. We used the double zeta-based split-valence basis set *Def2SVP*[5] for the description of non-metal atoms, C, N, O, and H, respectively. The Ni metal atom centre was described using the Stuttgart/Dresden type effective core potentials of SDD basis set[6] and the pseudopotentials was enabled to read from the input file by the addition of *Pseudo = Read* tag in the input route section. The SMD[7] implicit solvation model was employed to account for solvent effects on the N_2RR mechanism of the Nitpy molecular catalysts by describing the interaction between the solute charge density and the surrounding continuum solvent. The implicit solvent that we chose was water for the simulations. In addition, we carried out the vibrational frequency calculations on the optimized geometries to determine the thermodynamic parameters. For the evaluation of thermodynamic parameters, we implemented the quasi-harmonic approximations for the correction of low vibrational frequency modes by utilizing the quasi-harmonic

corrections for the enthalpy using Head-Gordon method[8], while the entropy corrections were made from Grimme's method[9].

The resulting reaction free energies were determined by assuming that the proton-coupled electron transfer (PCET) of N_2RR follows the computational hydrogen electrode model proposed by Nørksov *et al.*[10], in which the chemical potential (μ) of the proton and electron pair has been considered equal to the gas phase H_2 molecule demonstrated using the following equation.

$$H^+ + e^- \leftrightarrow H_2(g)$$

$$\text{where} \quad \mu(H^+) + \mu(e^-) = \frac{1}{2}\mu(H_2) - eU.$$

The reaction energies including ΔE and ΔG obtained from the DFT calculations for the N_2RR intermediates are tabulated in Table S4. Furthermore, we analyzed the oxidation states and electronic configuration of the Ni metal center for all the reaction intermediates using Multiwfn package.[11,12]

Besides, we predicted the redox potentials for the N_2RR reaction mechanism by employing Nernst equation.[13]

$$E^0_{R/O} = -\frac{\Delta G_r}{n_e F} - E^0(REF)$$

where ΔG_r is the free energy of reaction obtained from DFT at standard conditions, n_e is the number of electrons transferred during PCET process, and F is the Faraday's constant. The second term $E^0(REF)$ provides the potential of standard hydrogen electrode at the absolute scale, which was 4.4 V, respectively.

Periodic DFT: In addition to molecular calculations, we also executed the periodic DFT calculations in this work for the elucidation of reaction mechanism of N_2RR along with the energetics. The all-atom relaxations in the periodic DFT were performed with the help of generalized gradient approximation of Perdew-Burke-Ernzerhof[14] exchange and correlation functional under the plane-augmented-wave (PAW) formalism implemented in Vienna Ab-initio Simulation Package (VASP)[15-19] version 6.1.2. A kinetic energy cutoff of 400 eV was used for the plane wave expansion of the Kohn-Sham wave function. The ionic relaxations were carried out by applying the conjugate-gradient method and the convergence criterion between the electronic self-consistent energies was set to 10^{-4} eV. To enable the spin polarized calculations and to account for the magnetization, NSPIN=2 has been set in the input file and the magnetic moments of all the atoms involved were treated appropriately. To carry out the periodic DFT on the molecular catalysts, we placed Nitpy-based molecular catalysts in the center of a large supercell to avoid the interactions between the adjacent periodic images. The dimensions of the supercell were a = 23.947 Å, b = 26.577 Å, and c = 27.54 Å and α = β = γ = 90°, respectively. The integration of Brillouin zone was sampled with a 1×1×1 K-points using Monkhorst-Pack grid points.[20] The dispersion interactions are considered employing IVDW=12 in the input file according to Grimme dispersion corrections.[21-22] In addition, the dipole corrections were considered in the total energies by

setting IDIPOL = 1 in the input file for the evaluation of dipole moment analogous to the direction of first lattice vector. For calculating the phonon frequencies, we have used the finite difference method incorporated by enabling IBRION=5 tagline in the input script from which we evaluated the free energies of the reaction. Thus, the associated reaction energies calculated from the periodic DFT were represented in the following sections.

DFT calculations of electronic structure

DFT calculations of the electronic structure of the substituted Nitpy complexes were carried out using ORCA 6.1.1.[23-29] Geometry optimizations and vibrational frequency calculations were performed with the PBE0 hybrid functional, including D4 dispersion corrections and the def2-TZVP basis set. The optimized structures were verified by frequency analysis, and subsequent single-point calculations were performed using the same level of theory. The RIJCOSX approximation was employed to accelerate the calculations, with TightSCF used for SCF convergence and the DefGrid3 integration grid. Solvent effects were not included, as the calculations were intended to evaluate the intrinsic electronic structures of the molecular Ni complexes. All complexes were calculated as cationic species with an overall charge of +1 and singlet multiplicity.

Material Characterisation

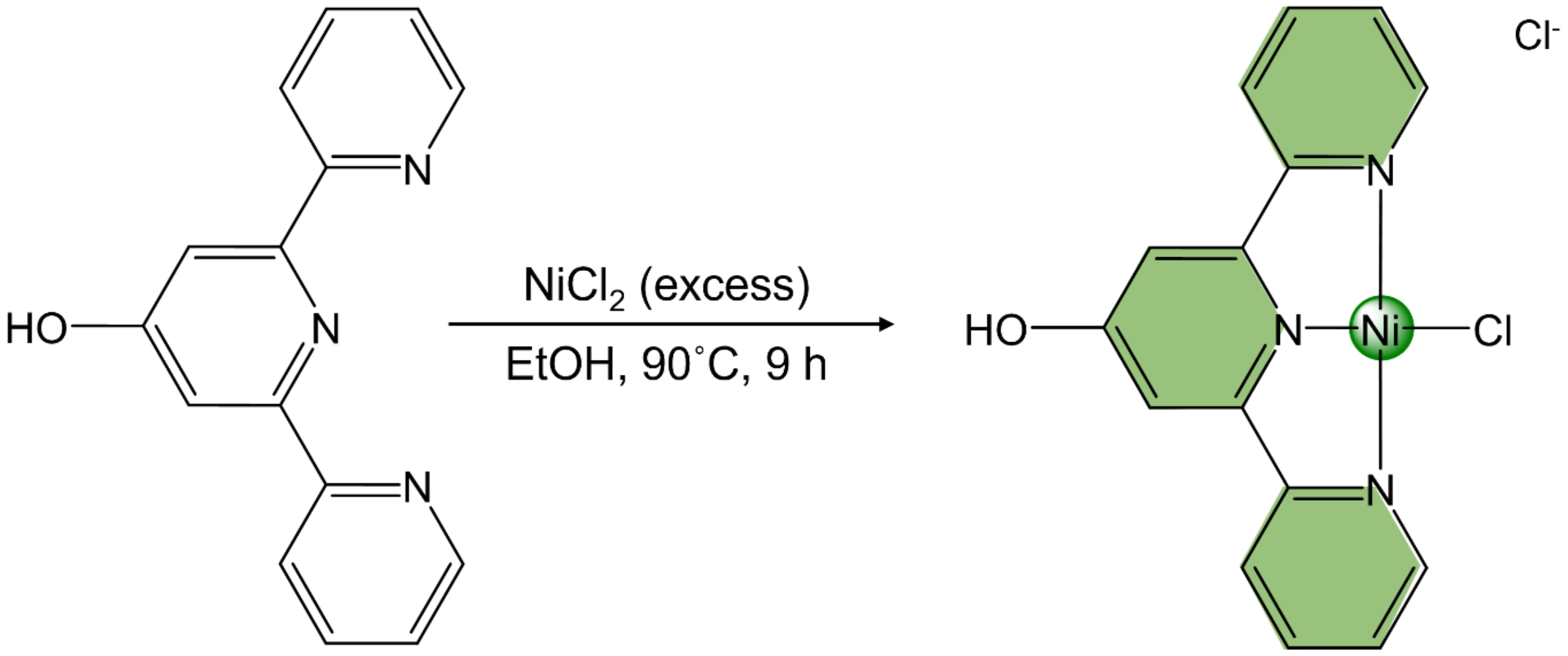

Figure S1. Reaction scheme for the synthesis of NitpyOH.

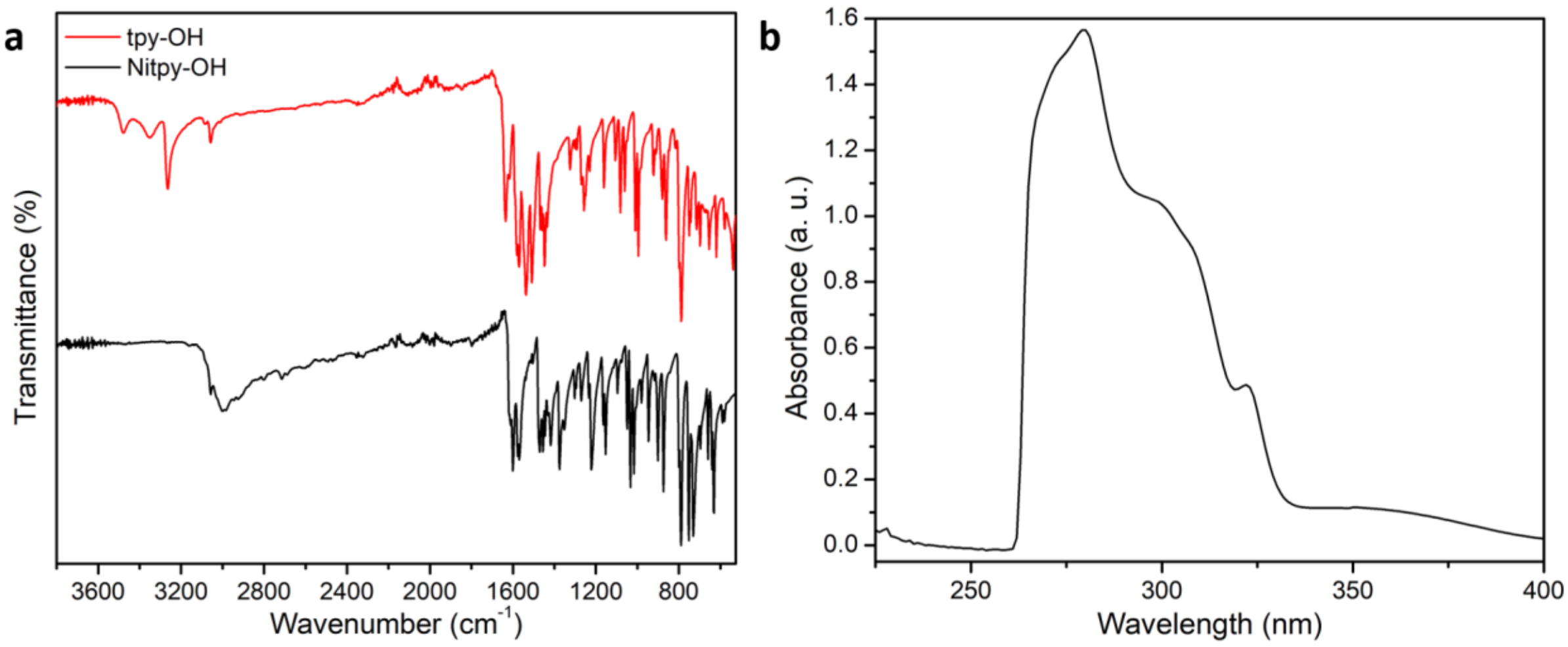

Figure S2. FTIR spectrum of tpy-OH and Nitpy-OH (a) and UV-Vis spectra of NitpyOH in DMF (b).

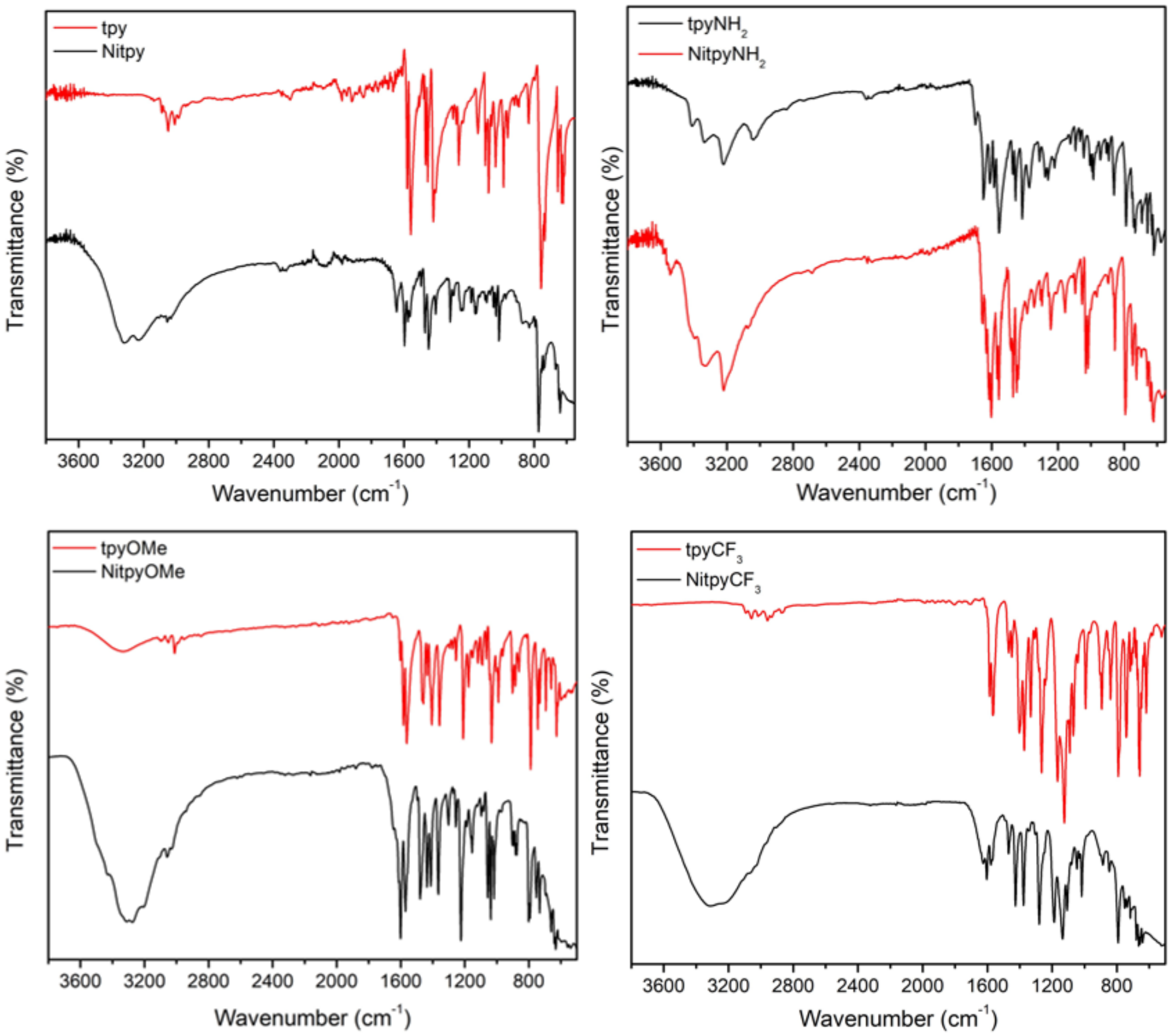

Figure S3. FTIR spectra of other Nitpy-based electrocatalysts and corresponding terpyridine starting material.

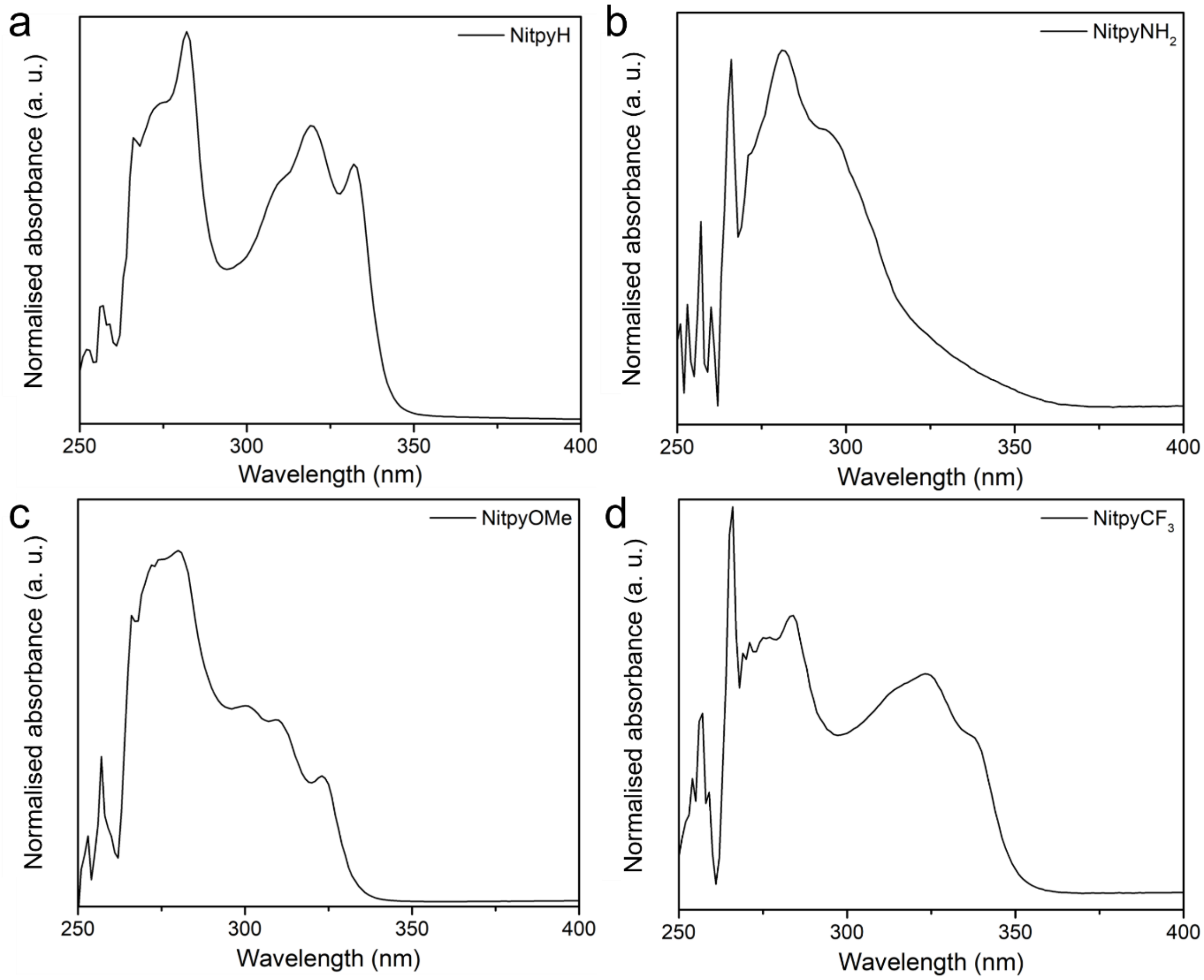

Figure S4. UV-Vis spectra of nickel terpyridine-based electrocatalysts in DMF. Nitpy (a), $NitpyNH_2$ (b), NitpyOMe (c) and $NitpyCF_3$ (d).

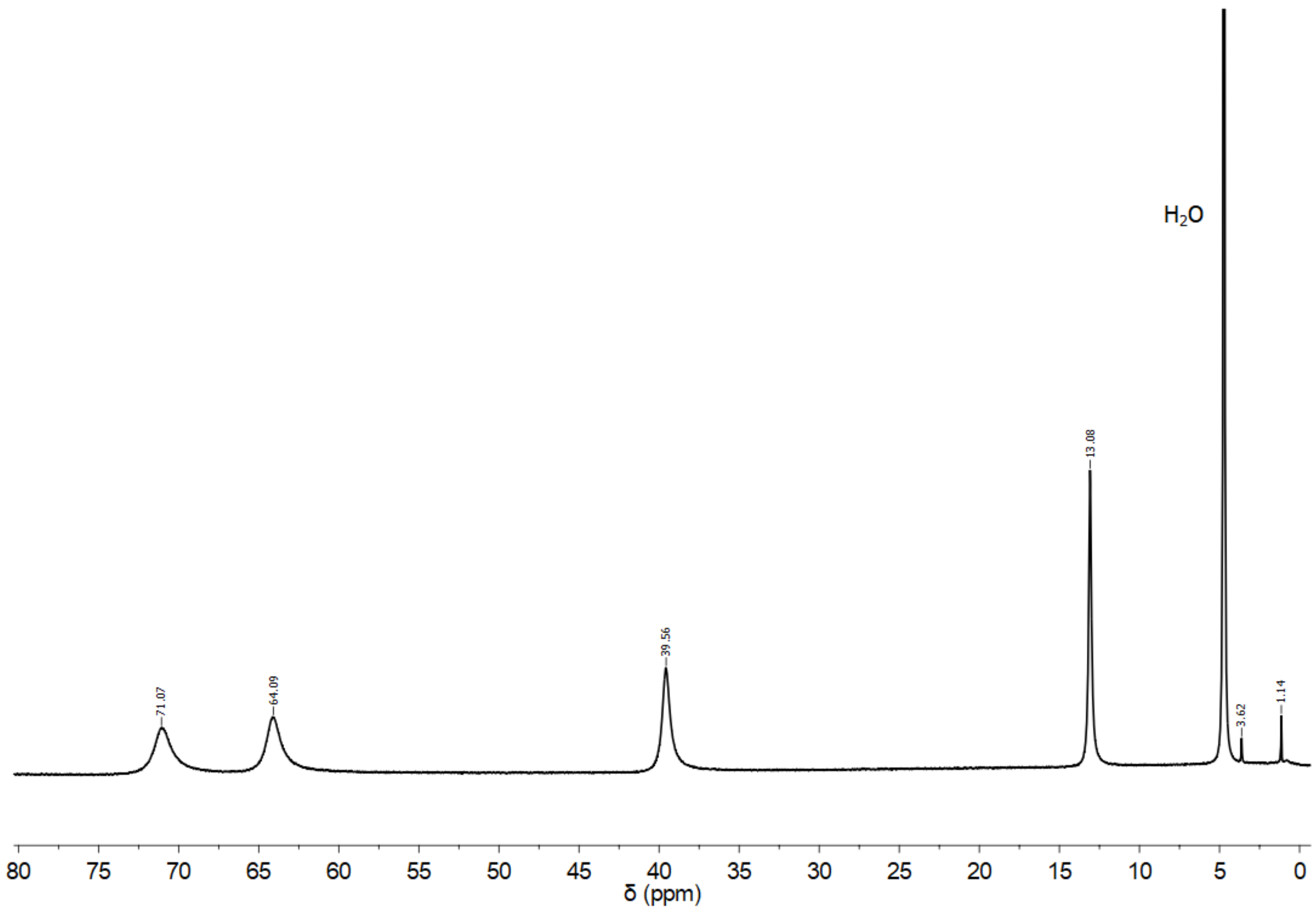

Figure S5. ^{1}H NMR spectrum of NitpyOH (500 MHz, D_2O, 298 K).

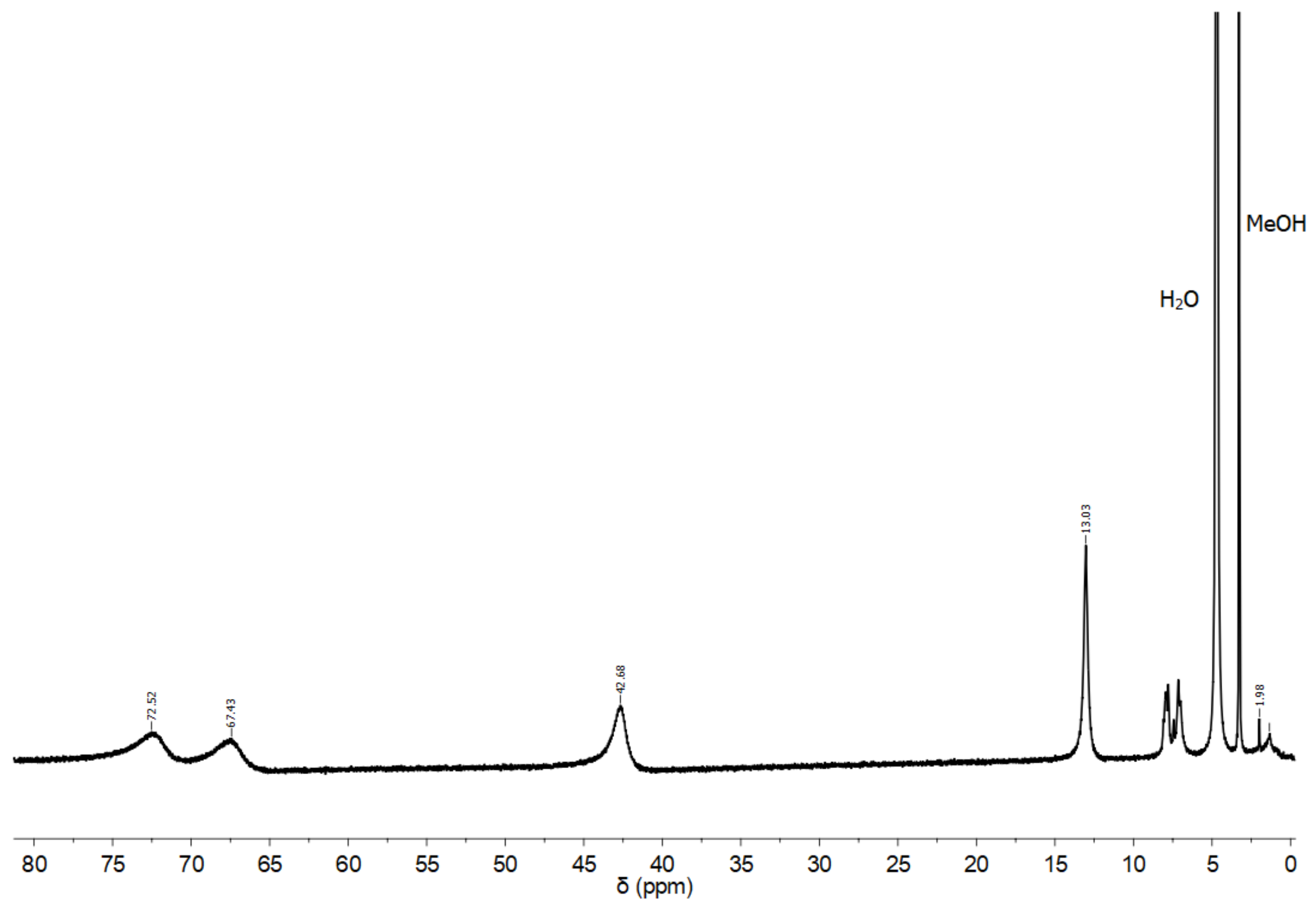

Figure S6. ^{1}H NMR spectrum of $Ni(tpyOH)_2$ (500 MHz, D_2O, 298 K).

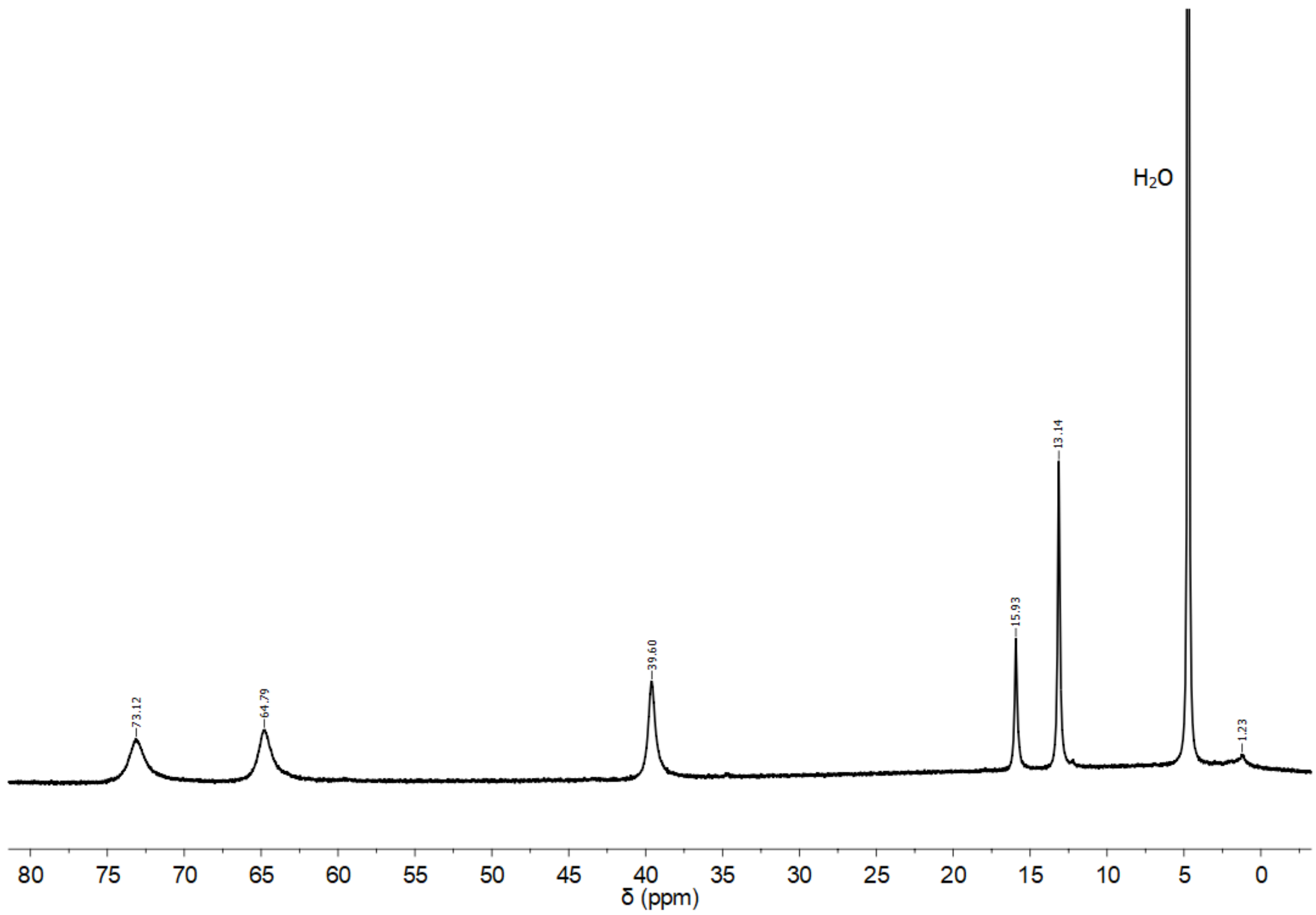

Figure S7. ^{1}H NMR spectrum of Nitpy (500 MHz, D_2O, 298 K).

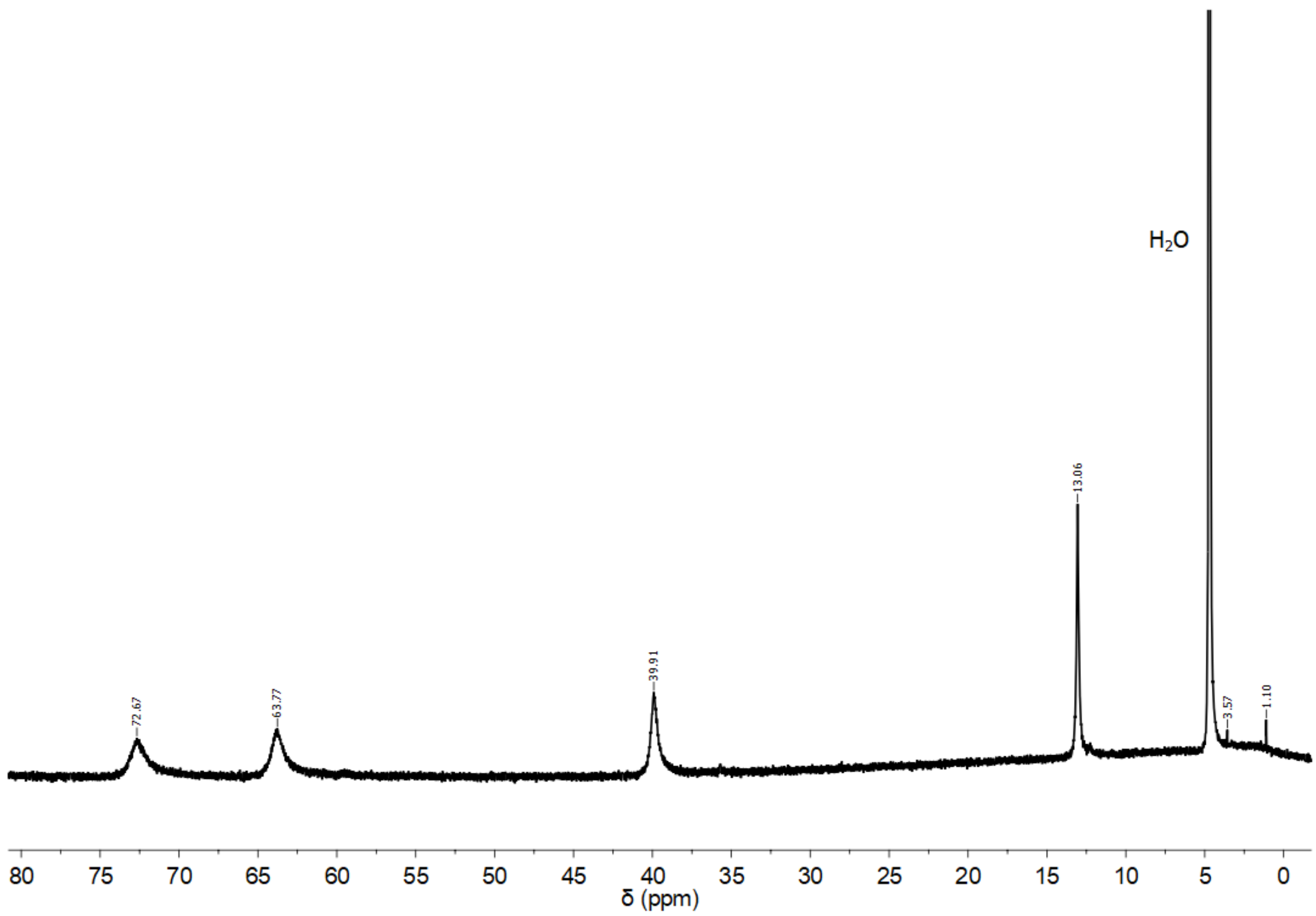

Figure S8. ^{1}H NMR spectrum of $NitpyNH_2$ (500 MHz, D_2O, 298 K).

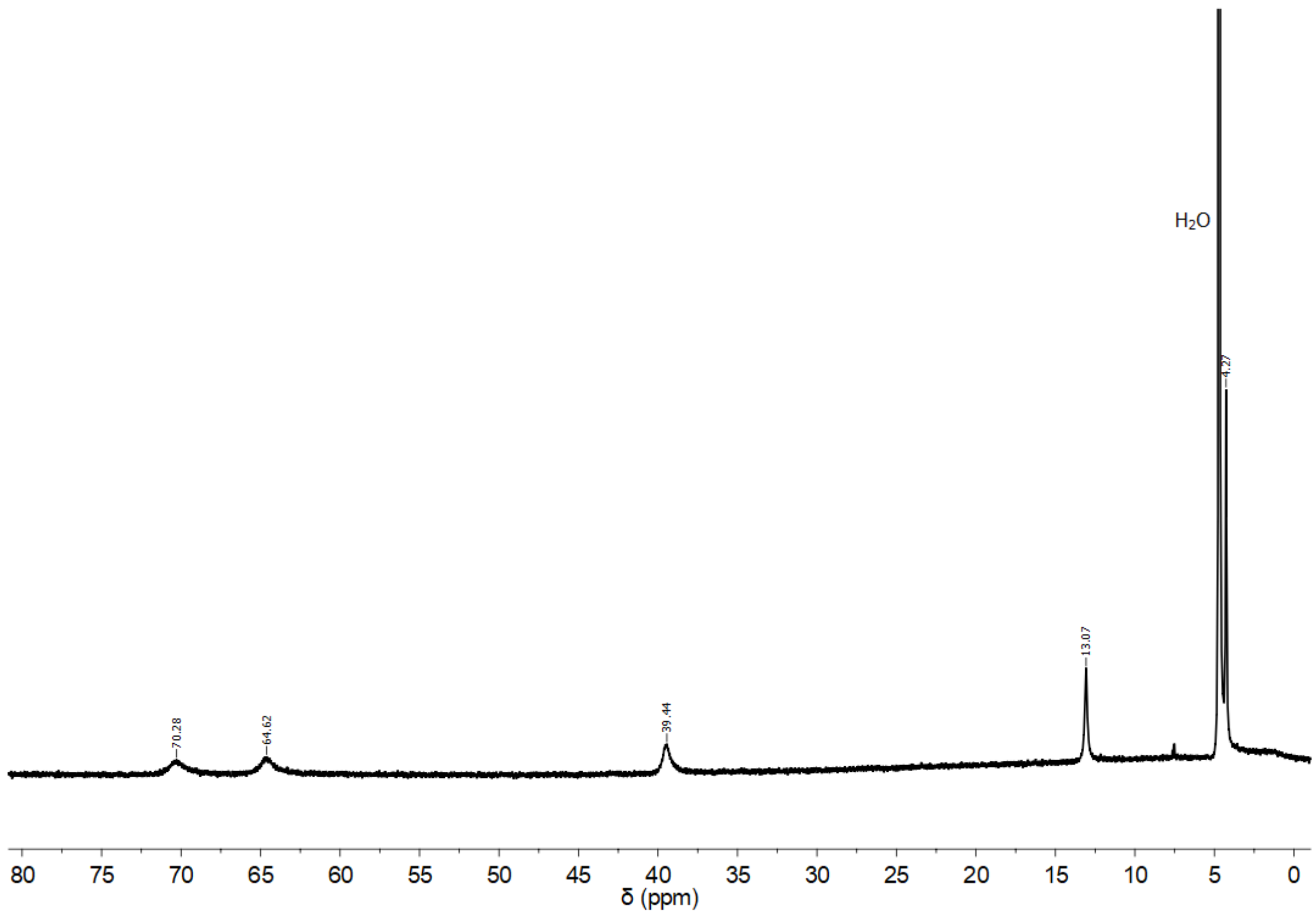

Figure S9. ^{1}H NMR spectrum of NitpyOMe (500 MHz, D_2O, 298 K).

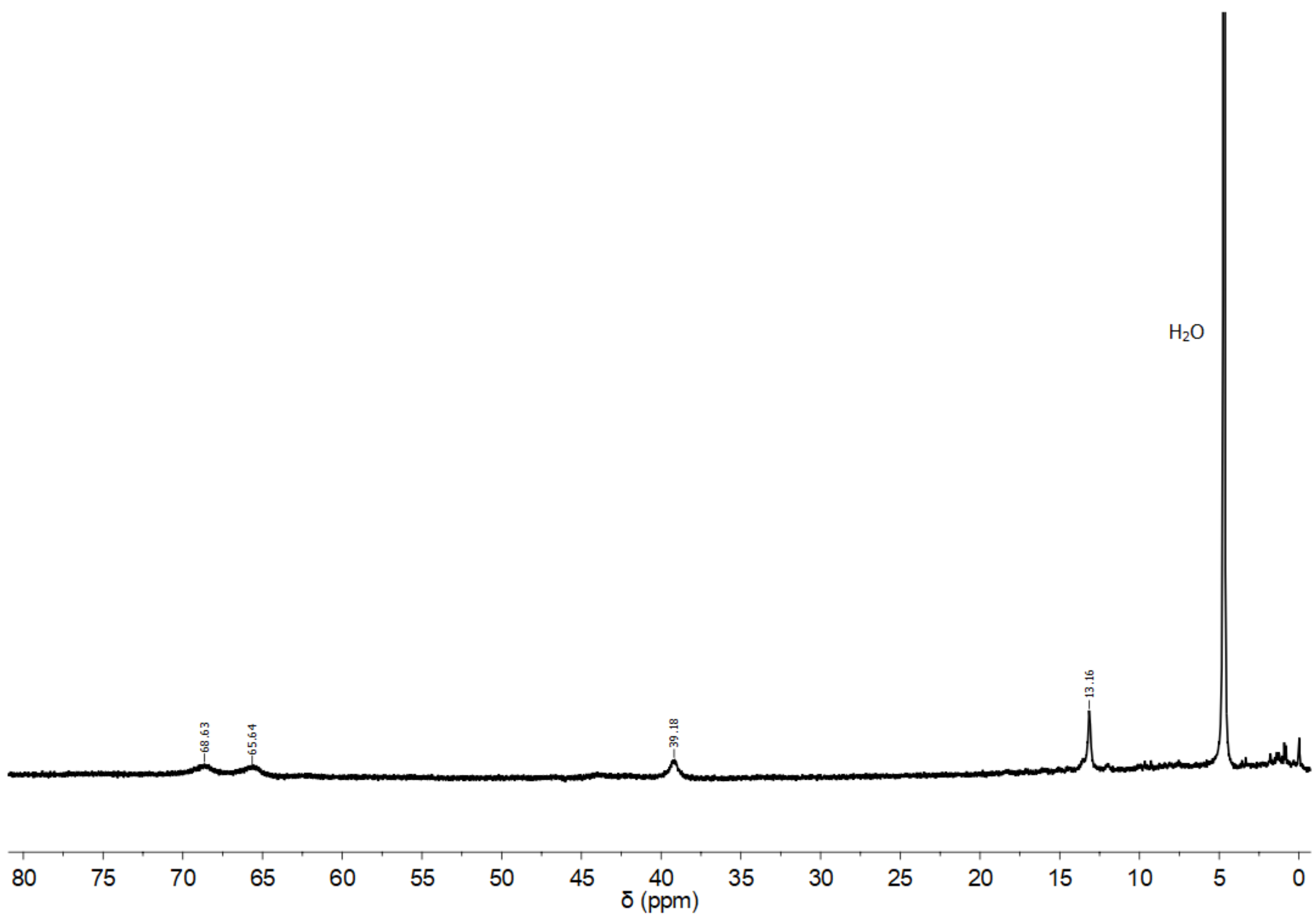

Figure S10. ^{1}H NMR spectrum of $NitpyCF_3$ (500 MHz, D_2O, 298 K).

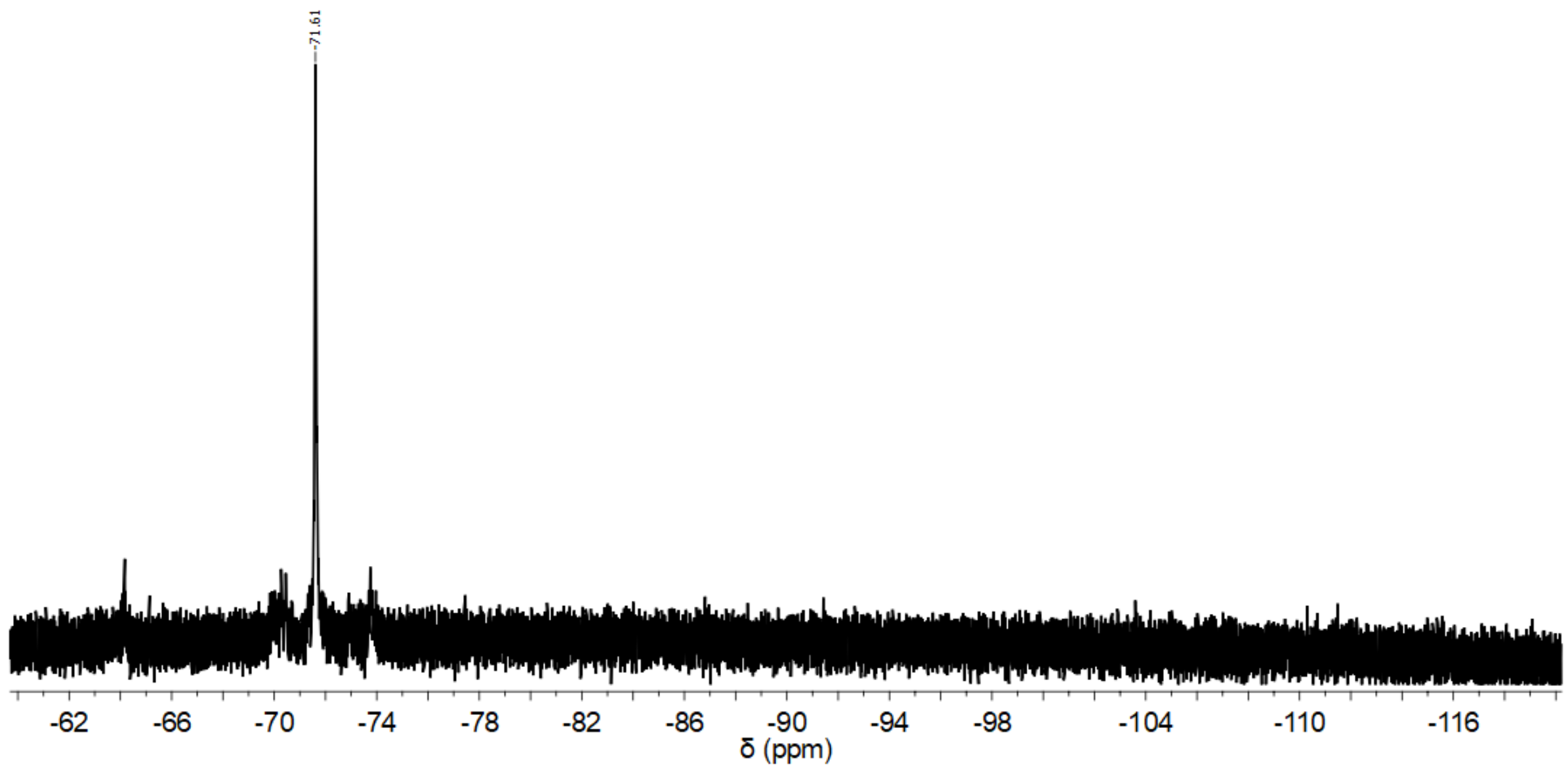

Figure S11. ^{19}F NMR spectrum of NitpyCF_3 (500 MHz, D_2O, 298 K).

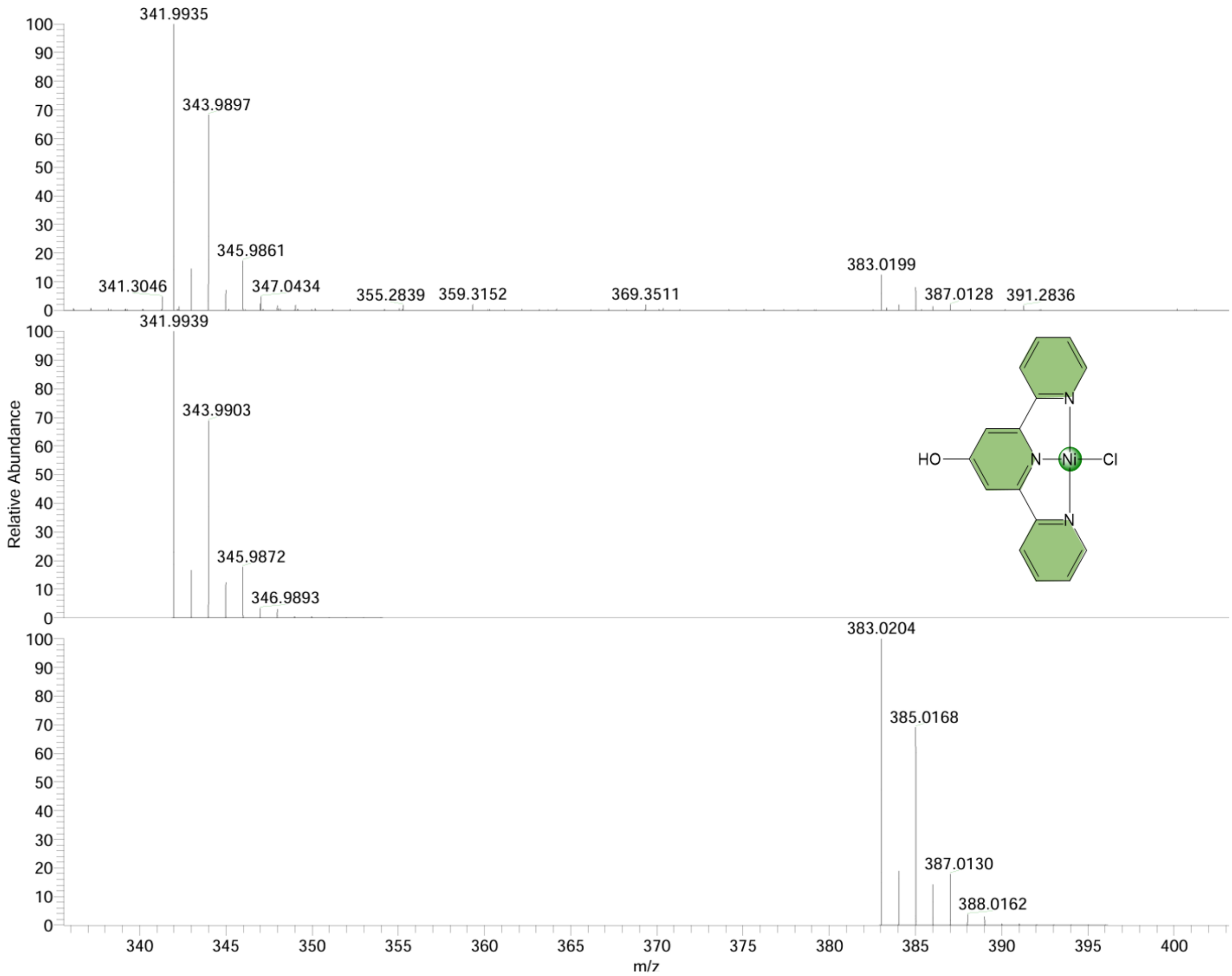

Figure S12. High-resolution mass spectrometric analysis of the pre-recrystallised NitpyOH showing the expected m/z ratio of (tpy-OH)NiCl. The bottom m/z pattern corresponds to the samples with one molecule of acetonitrile.

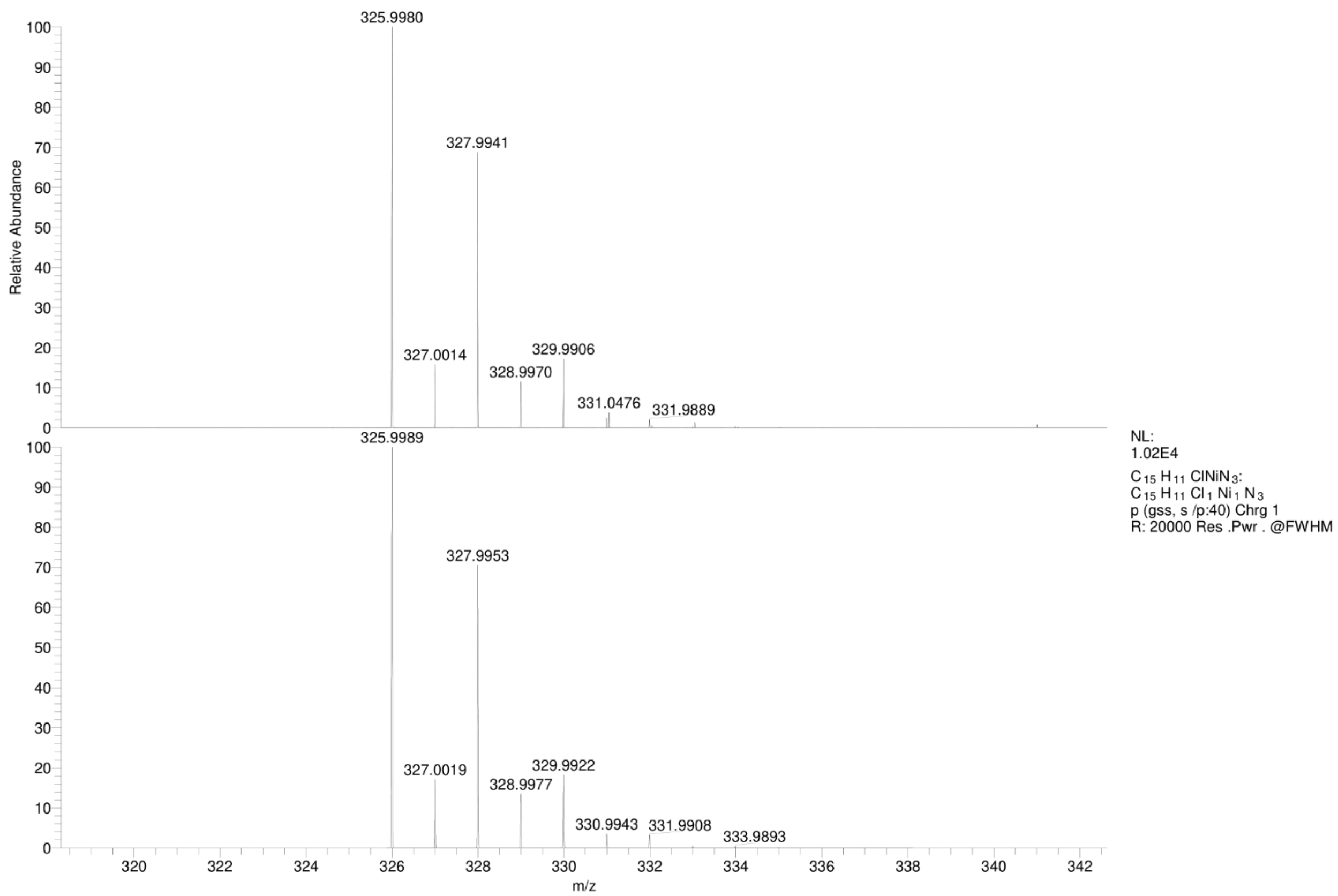

Figure S13. HR-MS ESI(+) spectrum of Nitpy.

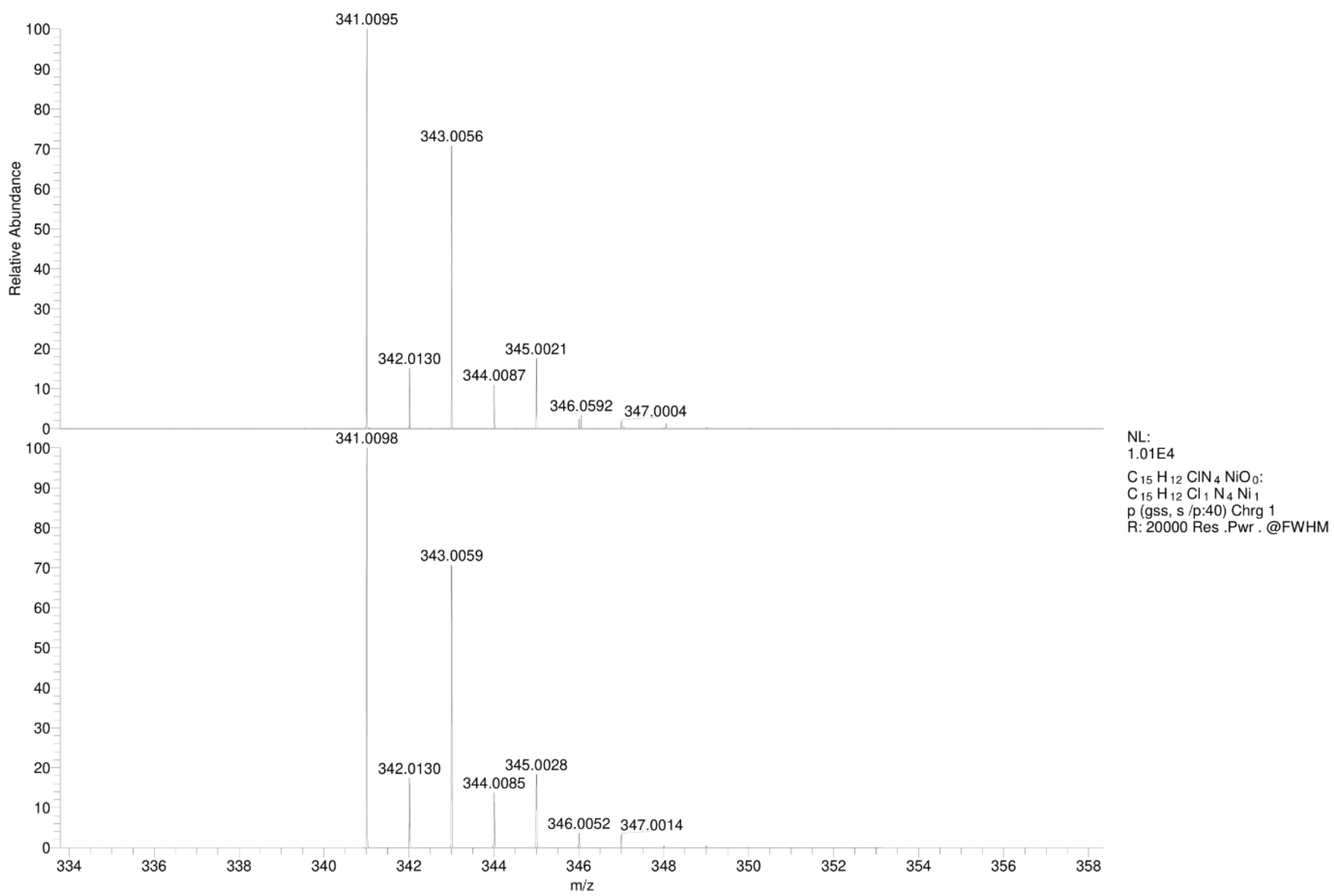

Figure S14. HR-MS ESI(+) spectrum of $NitpyNH_2$.

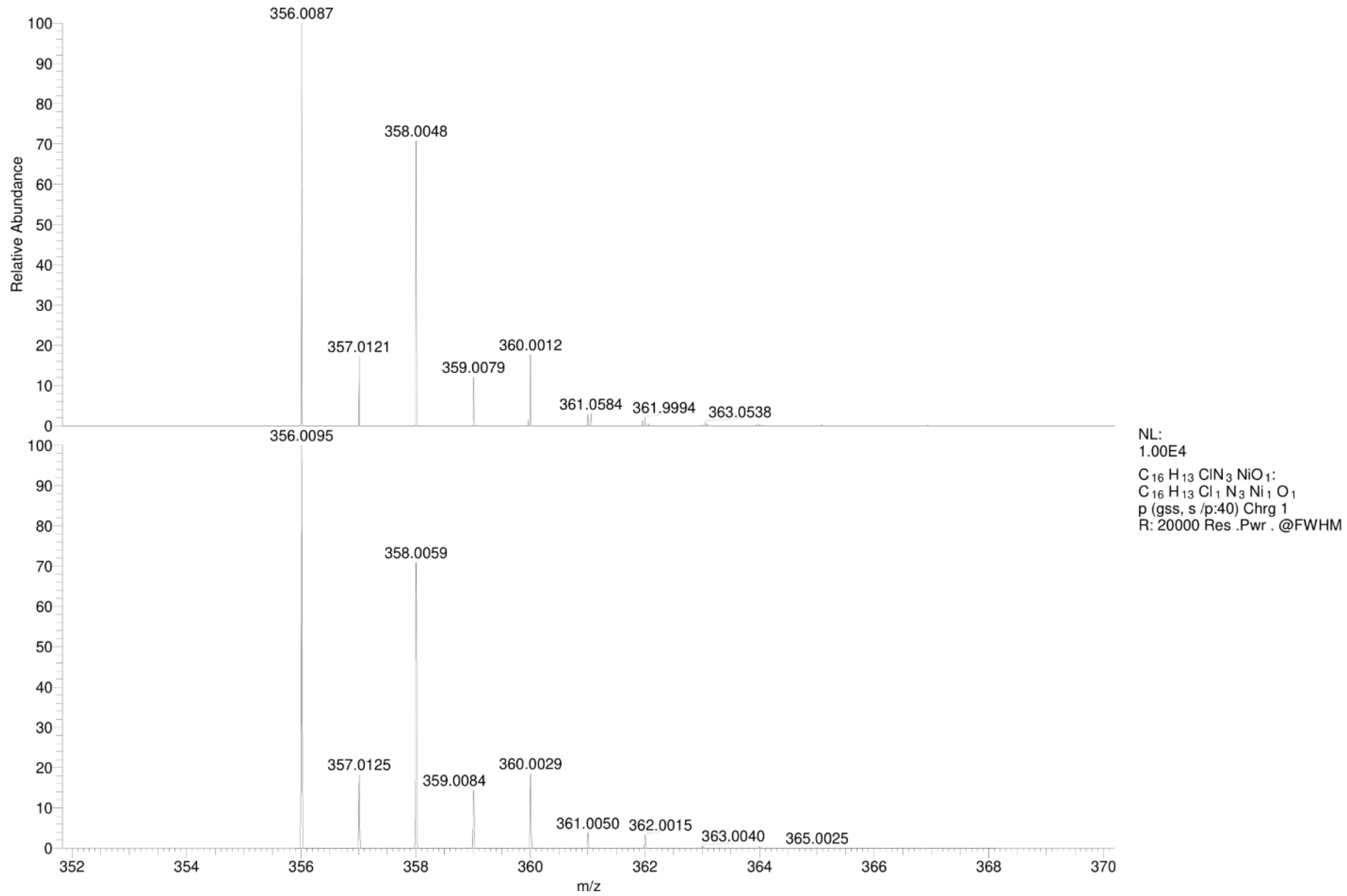

Figure S15. HR-MS ESI(+) spectrum of NitpyOMe.

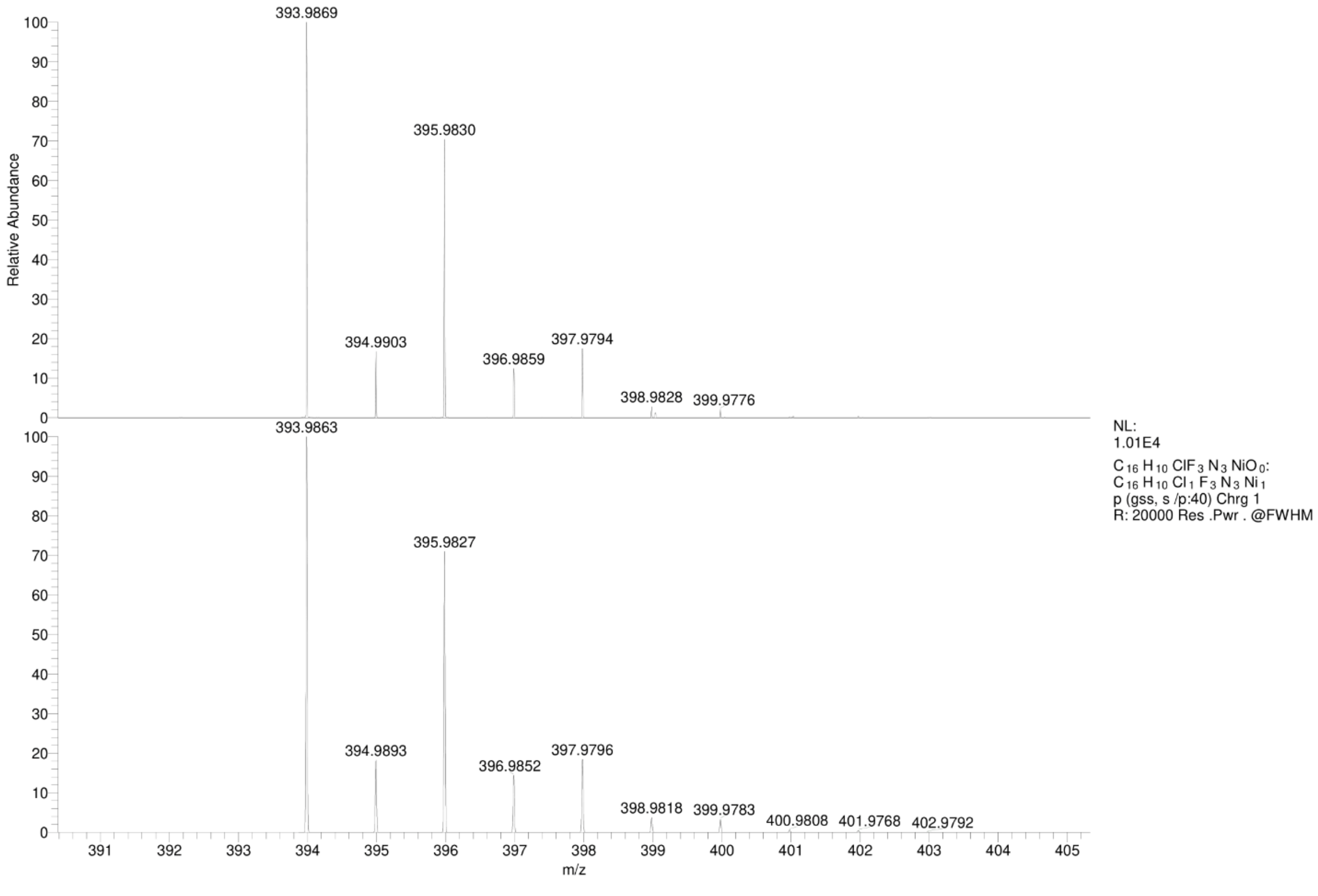

Figure S16. HR-MS ESI(+) spectrum of NitpyCF_3.

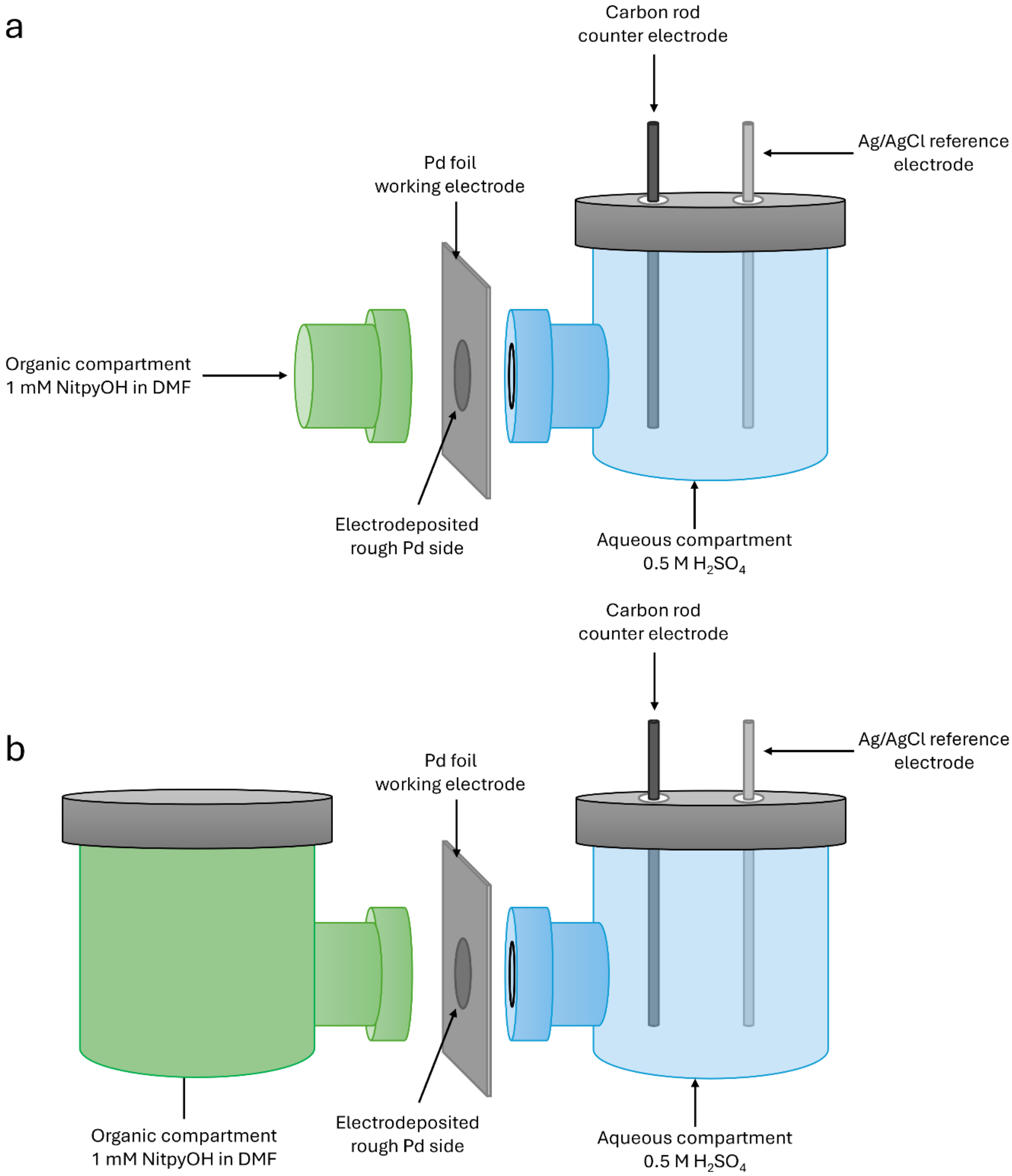

Figure S17. Simple schematic of the electrochemical Pd-membrane reactor used for the reduction of dinitrogen with NitpyOH as the catalyst for typical electrochemical reactions (a) and stability measurements (b).

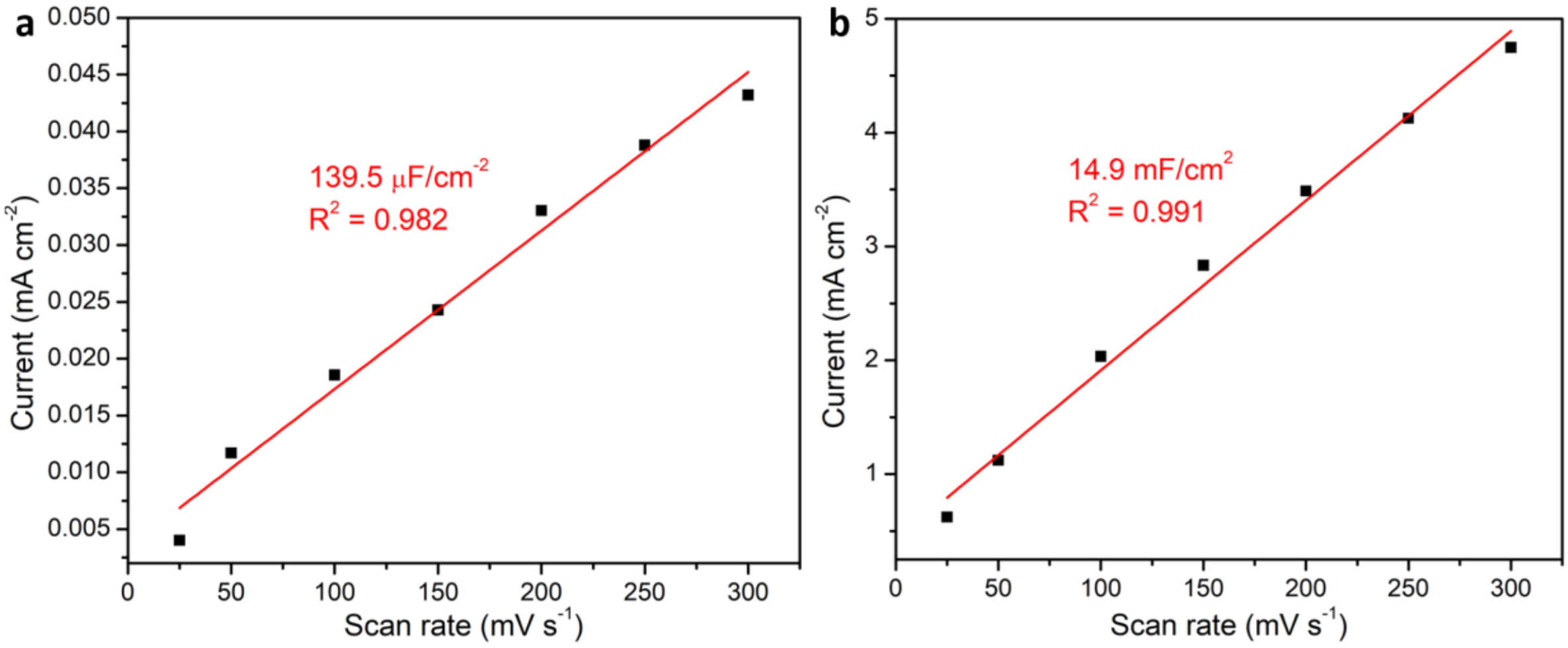

Figure S18. ECSA measurements. Double-layer capacitance measurements of the planar Pd foil electrode (a) and the electrodeposited Pd surface (b) showing a 107-fold increase in surface area.

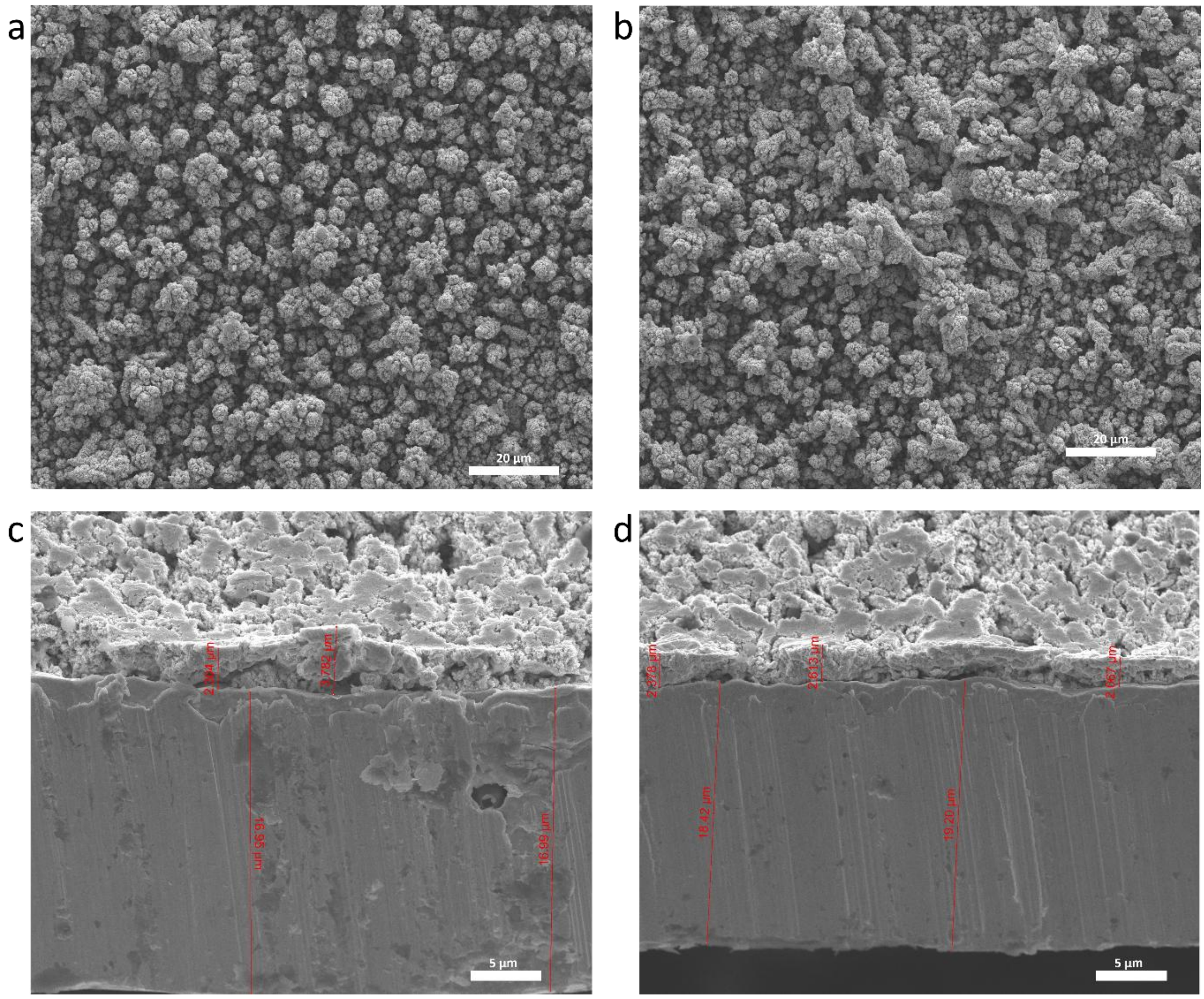

Figure S19. SEM images of the Pd membrane before (a) and after (b) catalysis and cross-section of the electrodeposited Pd foil electrode (c, d).

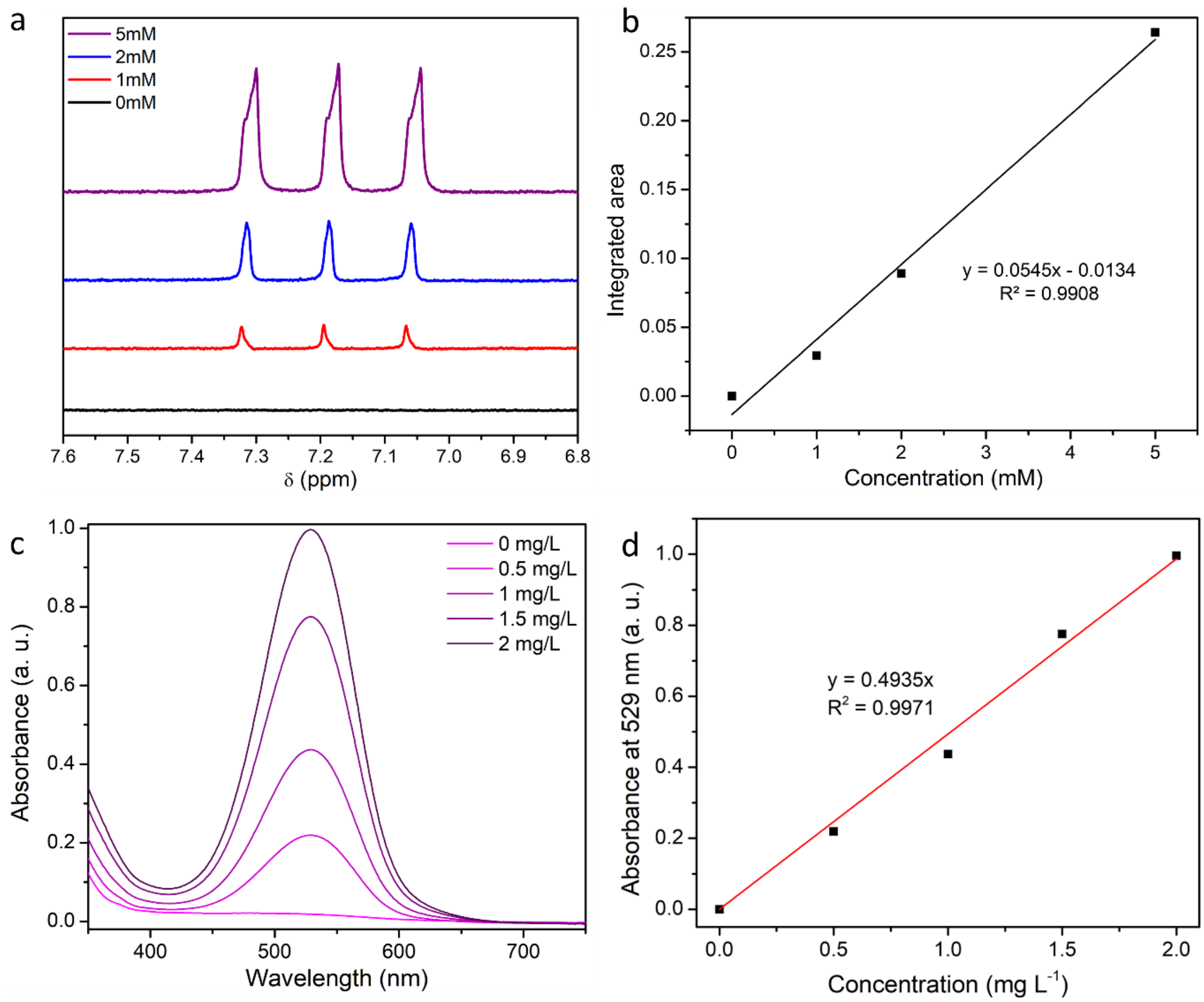

Figure S20. NMR spectra of varying concentrations of ammonia (a) and NMR calibration curve for ammonia (b). The relative peak area is plotted vs. that of the maleic acid internal standard. UV-Vis spectra of sodium nitrite at different concentrations (c) and calibration curve for nitrite determination using an absorbance of 529 nm (d).

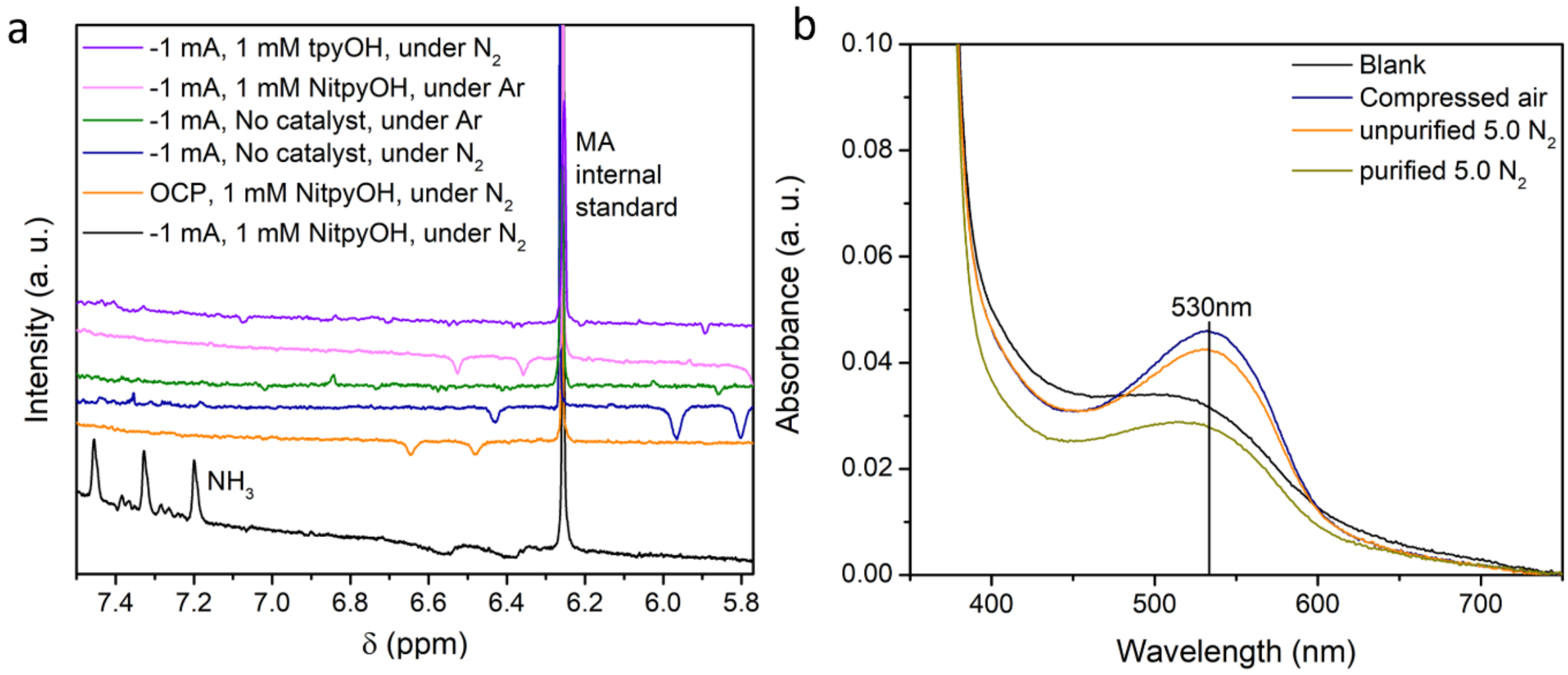

c

Gas source	NO_x concentration in trap (µM)	Total NO_x captured (nmol)
Compressed air	4.66	46.6
5.0 grade N_2 (unpurified)	4.31	43.1
5.0 grade N_2 (purified)	Below limit of detection	Below limit of detection
Blank trap (no gas)	Below limit of detection	Below limit of detection

Figure S21. NMR spectra (a) of reaction solution after dinitrogen reduction reaction with NitpyOH and Pd membrane at -1 mA for 5 h with a maleic acid (MA) internal standard. Spectra are compared with blank control experiments conducted under open-circuit potential (OCP), in the absence of catalyst under N_2 and Ar, 1 mM NitpyOH under an argon atmosphere and 1 mM tpyOH free ligand under a dinitrogen atmosphere. UV-Vis spectra (b) of alkaline trap solutions following exposure to compressed air, unpurified 5.0 grade N_2, purified 5.0 grade N_2, and a sealed blank trap, after nitrate reduction and addition of Griess reagents. Absorbance at 529 nm corresponds to total NO_x concentration. Table (c) of quantification of NO_x species in feed gases.

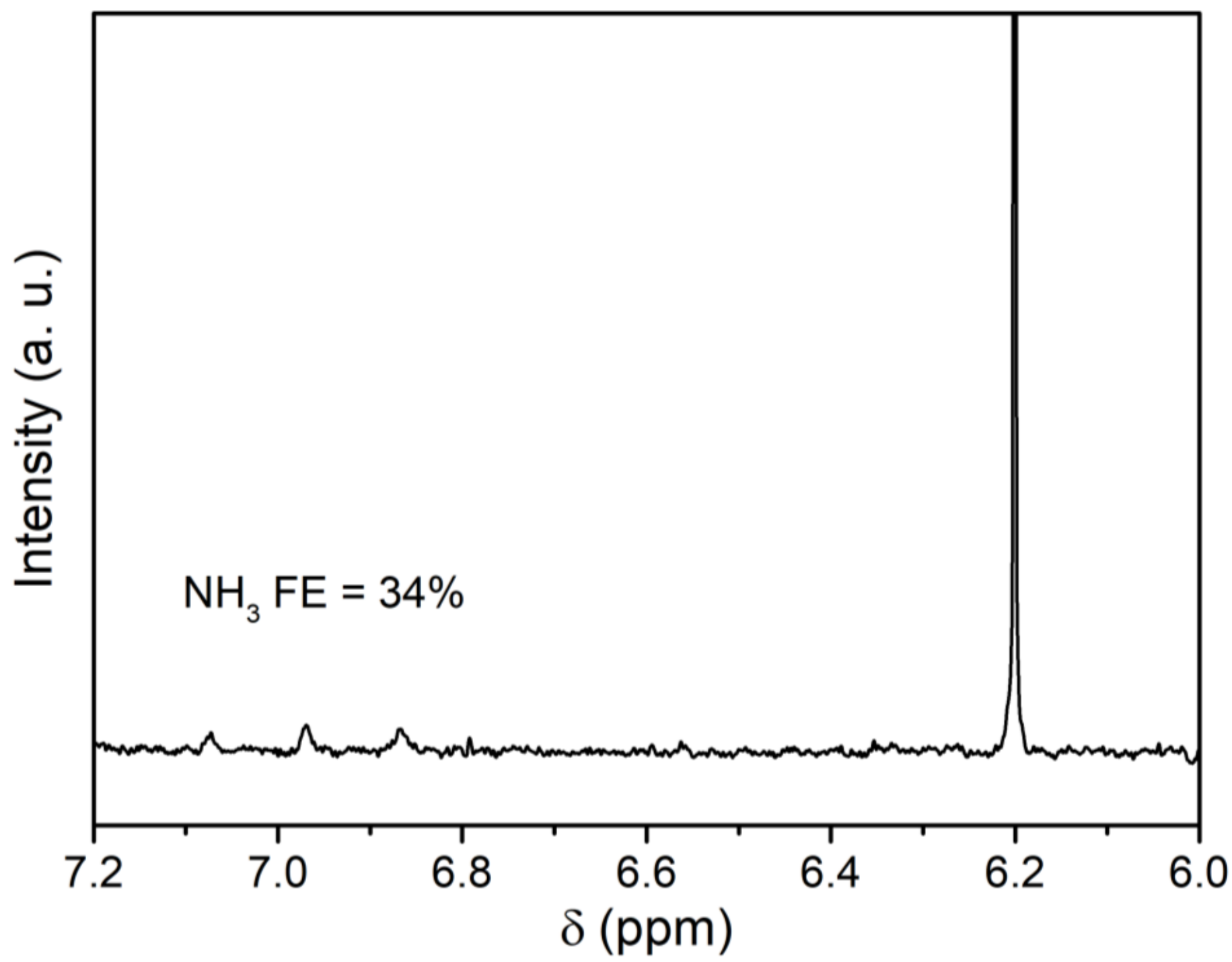

Figure S22. NMR spectra of reaction solution after nitrogen reduction reaction in acetic acid instead of DMF at -1 mA for 5 h.

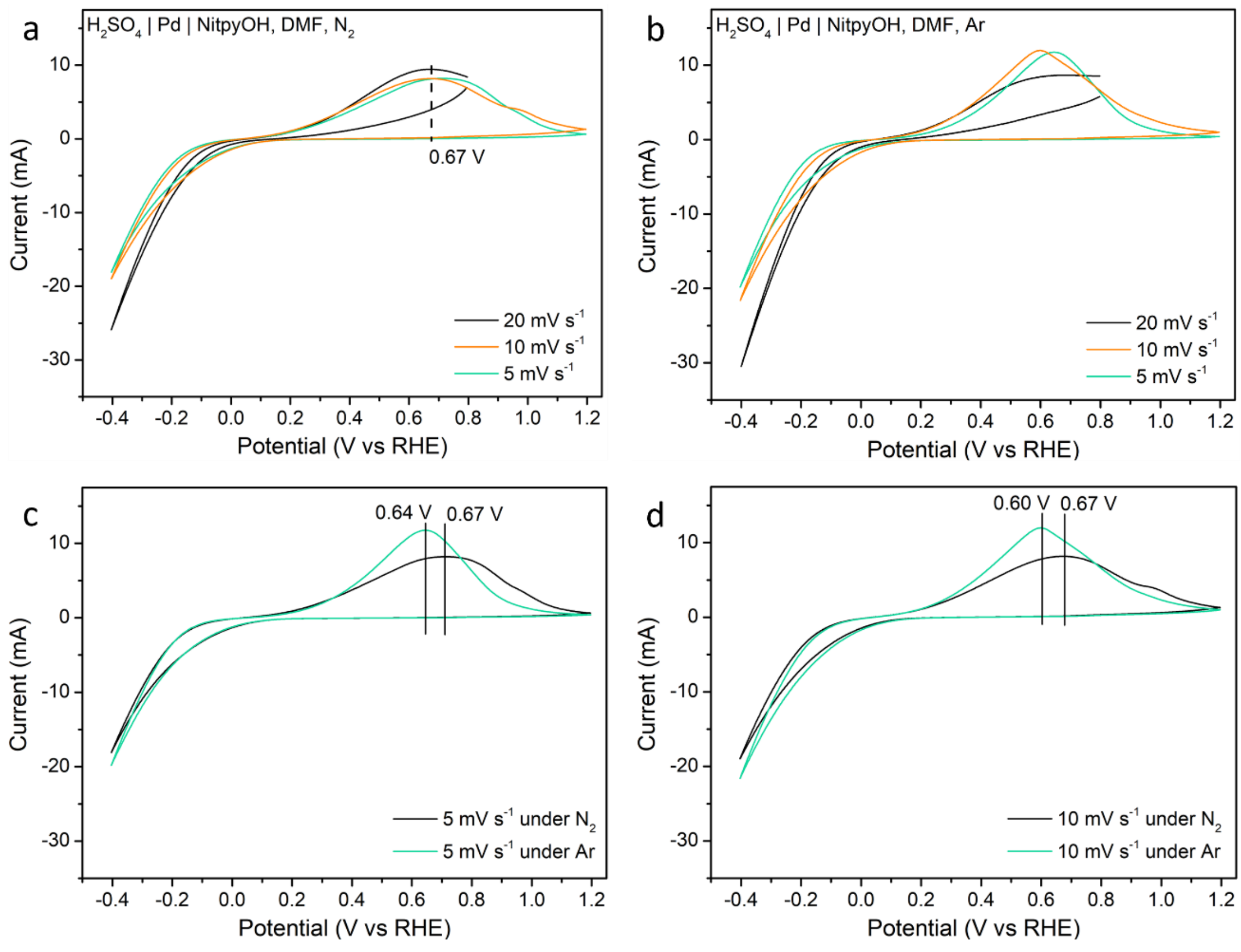

Figure S23. Cyclic voltammograms for the palladium membrane reactor with 1 mM NitpyOH in DMF on the roughened side at different scan rates under N_2 (a) and Ar (b). Comparison under an N_2 and Ar atmosphere at 5 mV s^{-1} (c) and 10 mV s^{-1} (d).

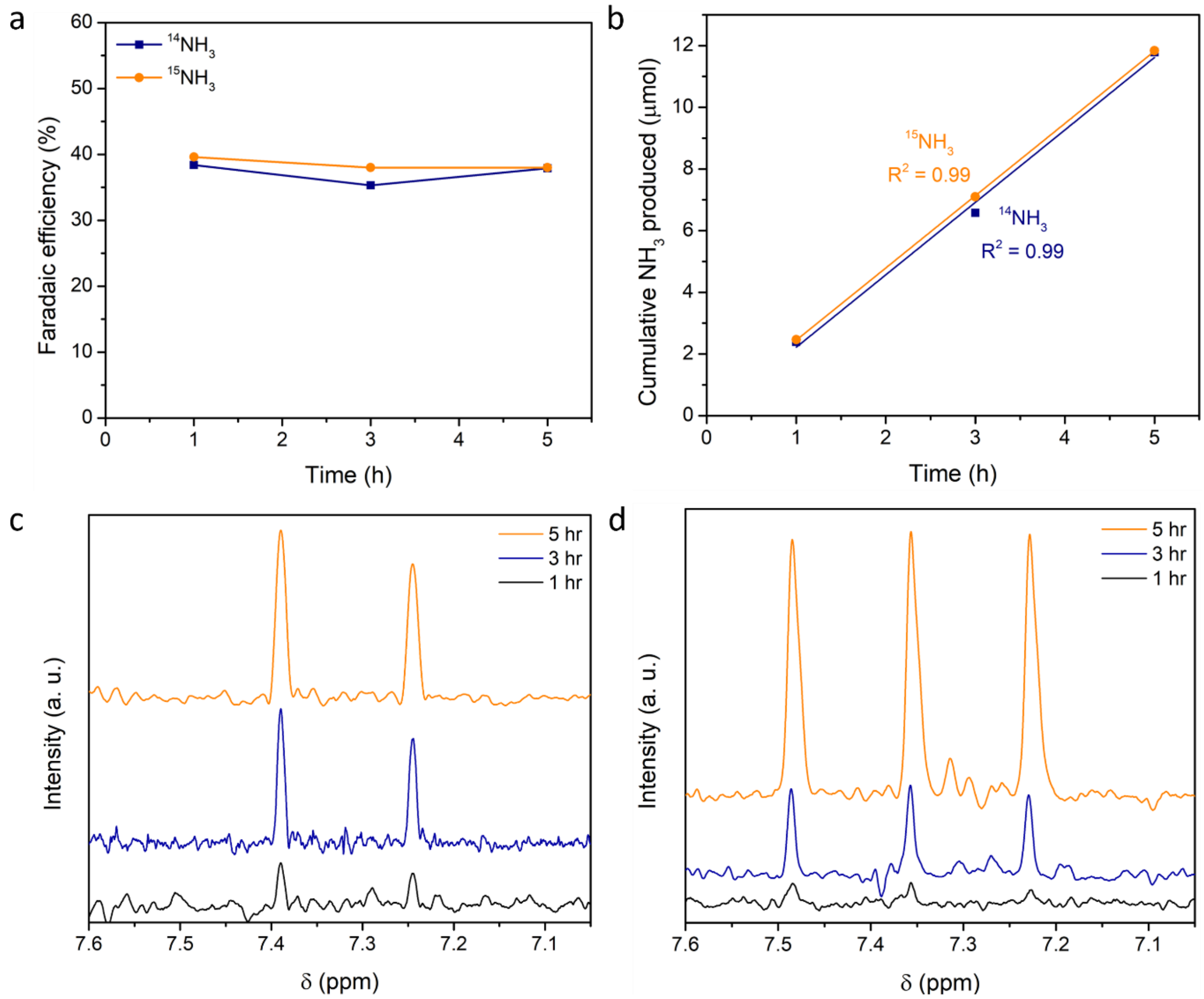

Figure S24. Faradaic efficiency for NH_3 production over time (a), cumulative NH_3 production as a function of electrolysis time (b), time-dependent ^1H NMR showing the progressive increase in the $^{15}NH_4^+$ signal during electrolysis (c) and $^{14}NH_4^+$ signal during electrolysis (d).

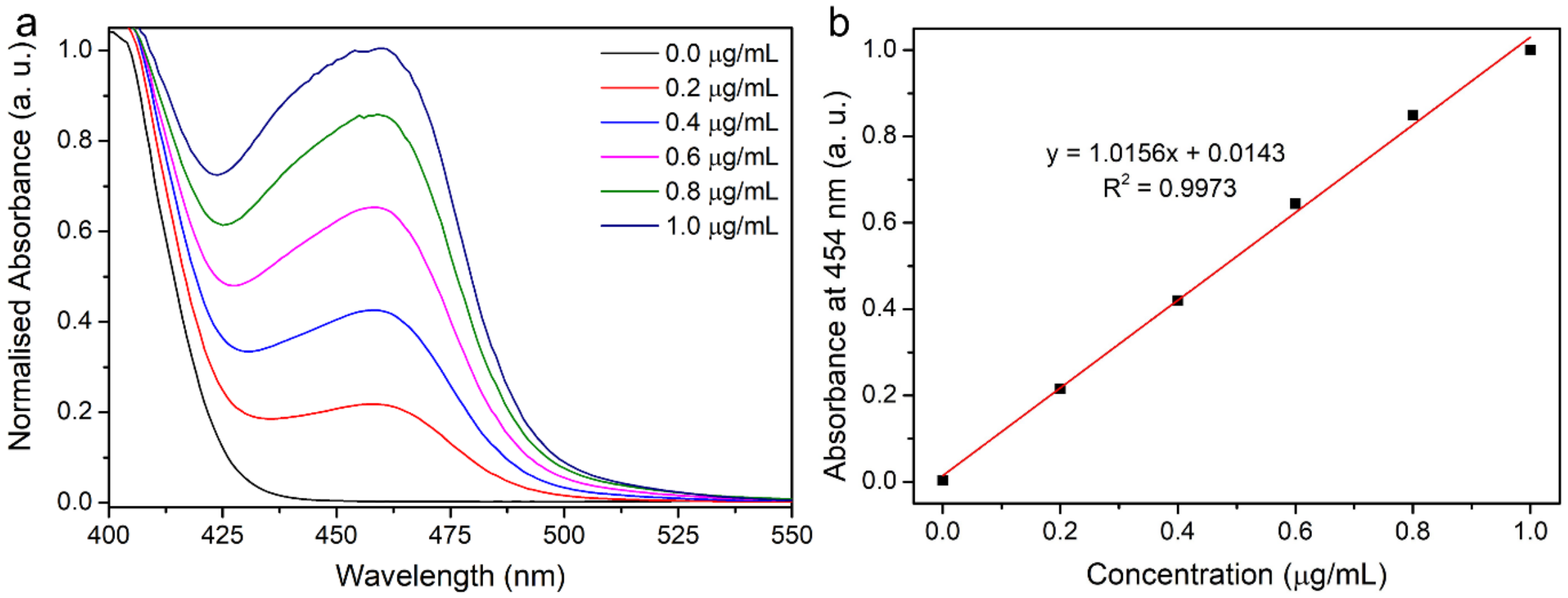

Figure S25. UV-Vis spectra of hydrazine at different concentrations (a) and calibration curve for hydrazine determination using the absorbance at 454 nm (b).

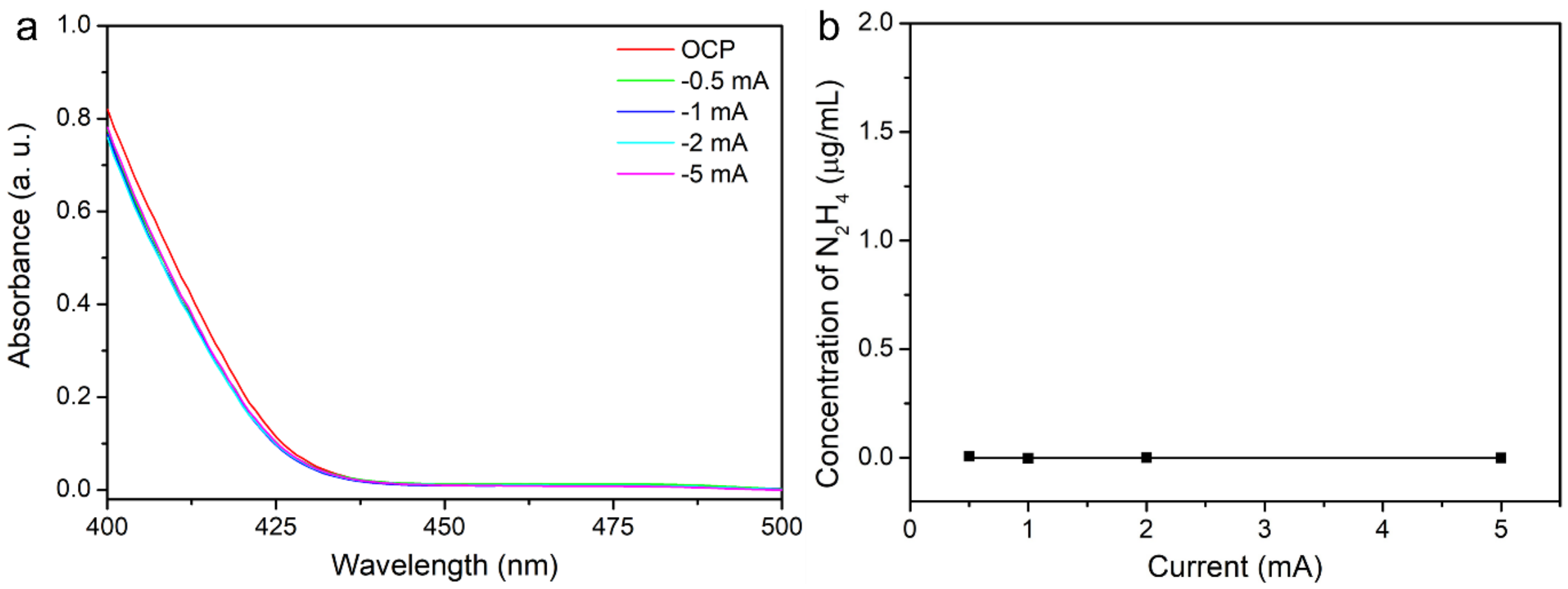

Figure S26. UV-Vis spectra (a) and the concentration of N_2H_4 detected after 5 hours of electrolysis at various applied currents.

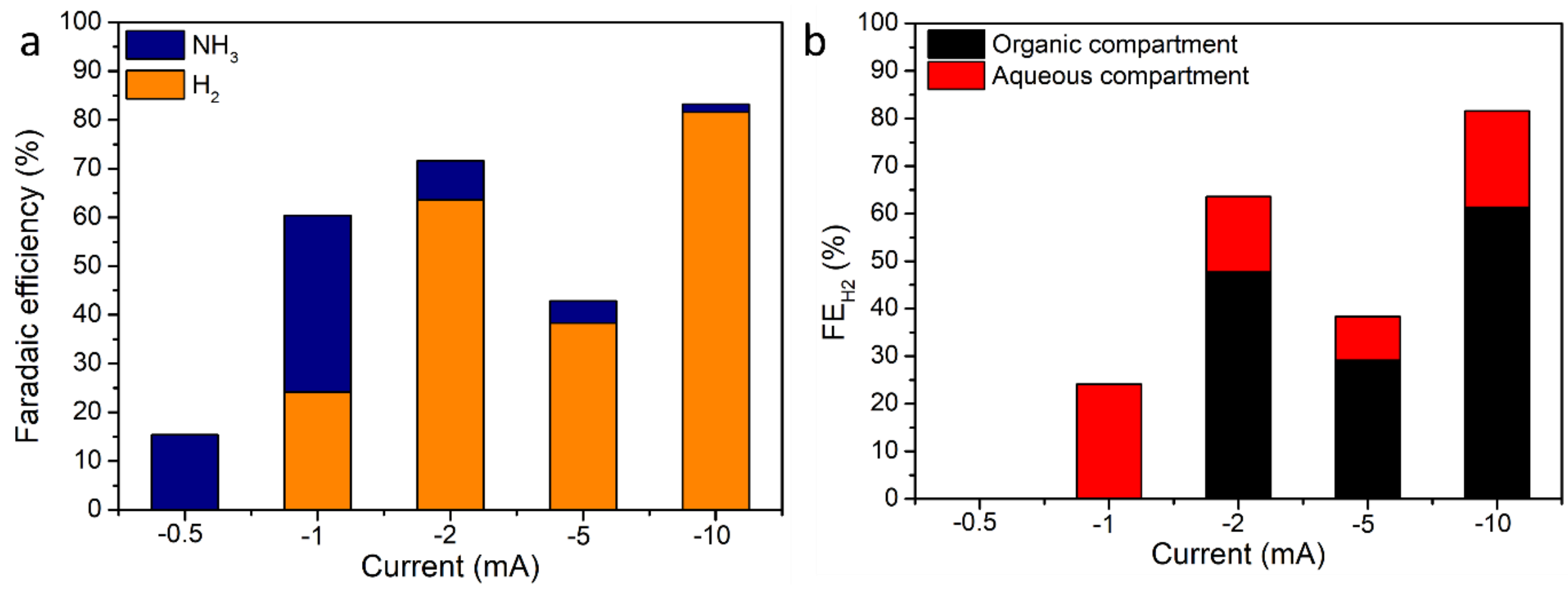

Figure S27. Faradaic efficiencies for all detected products (a) and for the production of H_2 comparing the organic and aqueous compartments of the H-cell (b) at various applied currents.

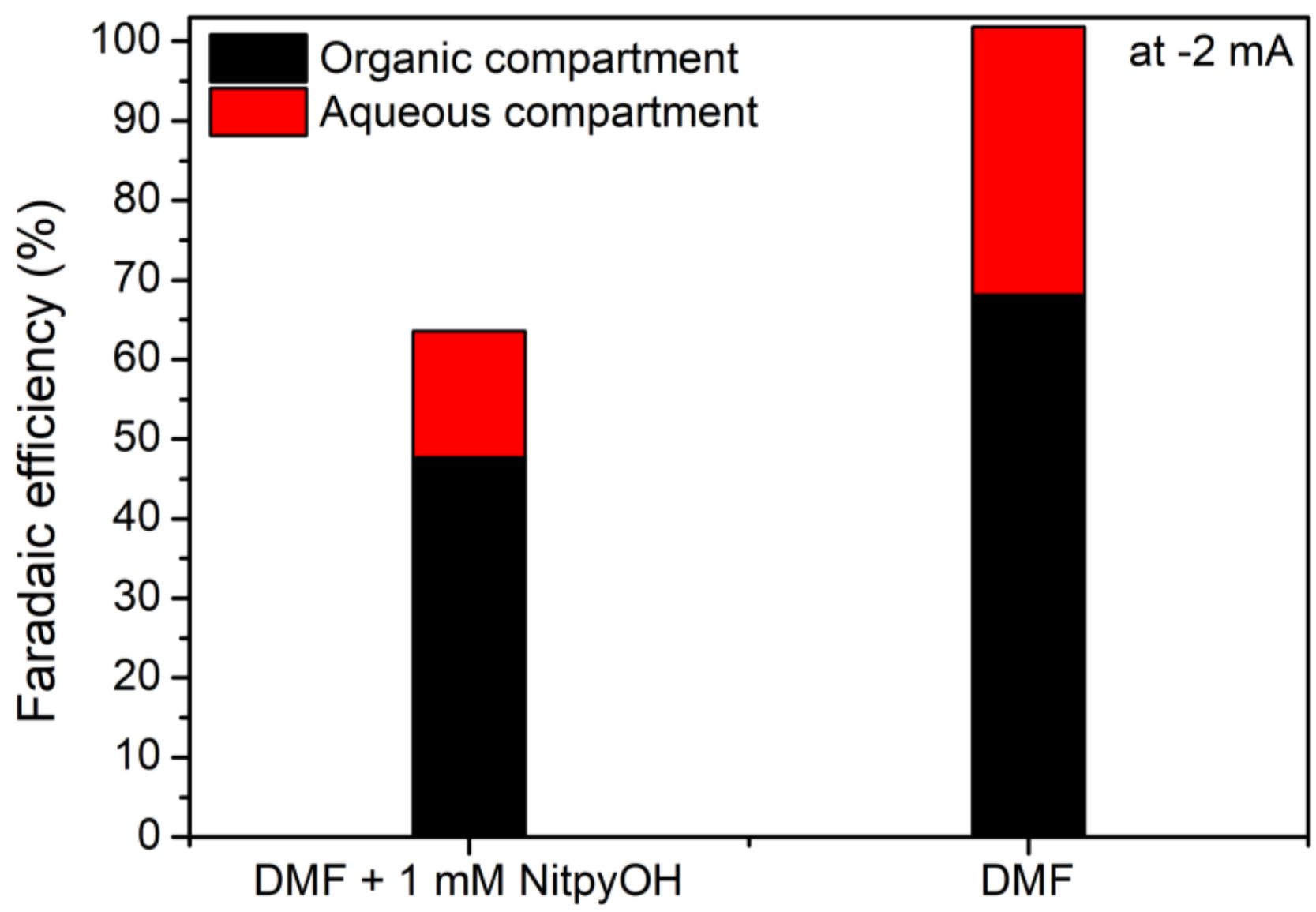

Figure S28. Faradaic efficiencies for the production of H_2 at -2 mA applied current with 1 mM of NitpyOH purged with N_2 compared to using DMF without catalyst.

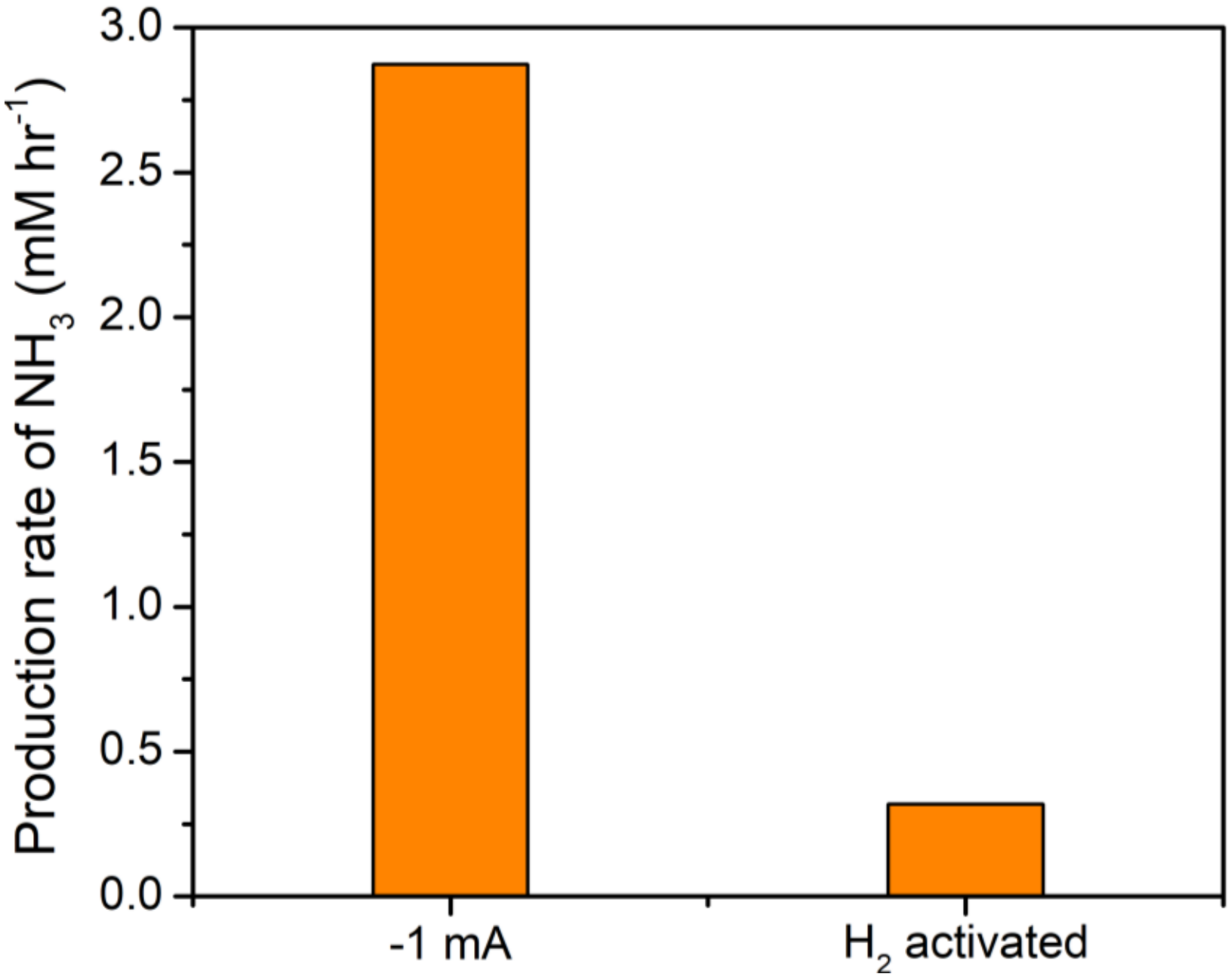

Figure S29. Bar chart showing the production rate of ammonia comparing the use of electrochemical hydrogenation and hydrogen hydrogenation.

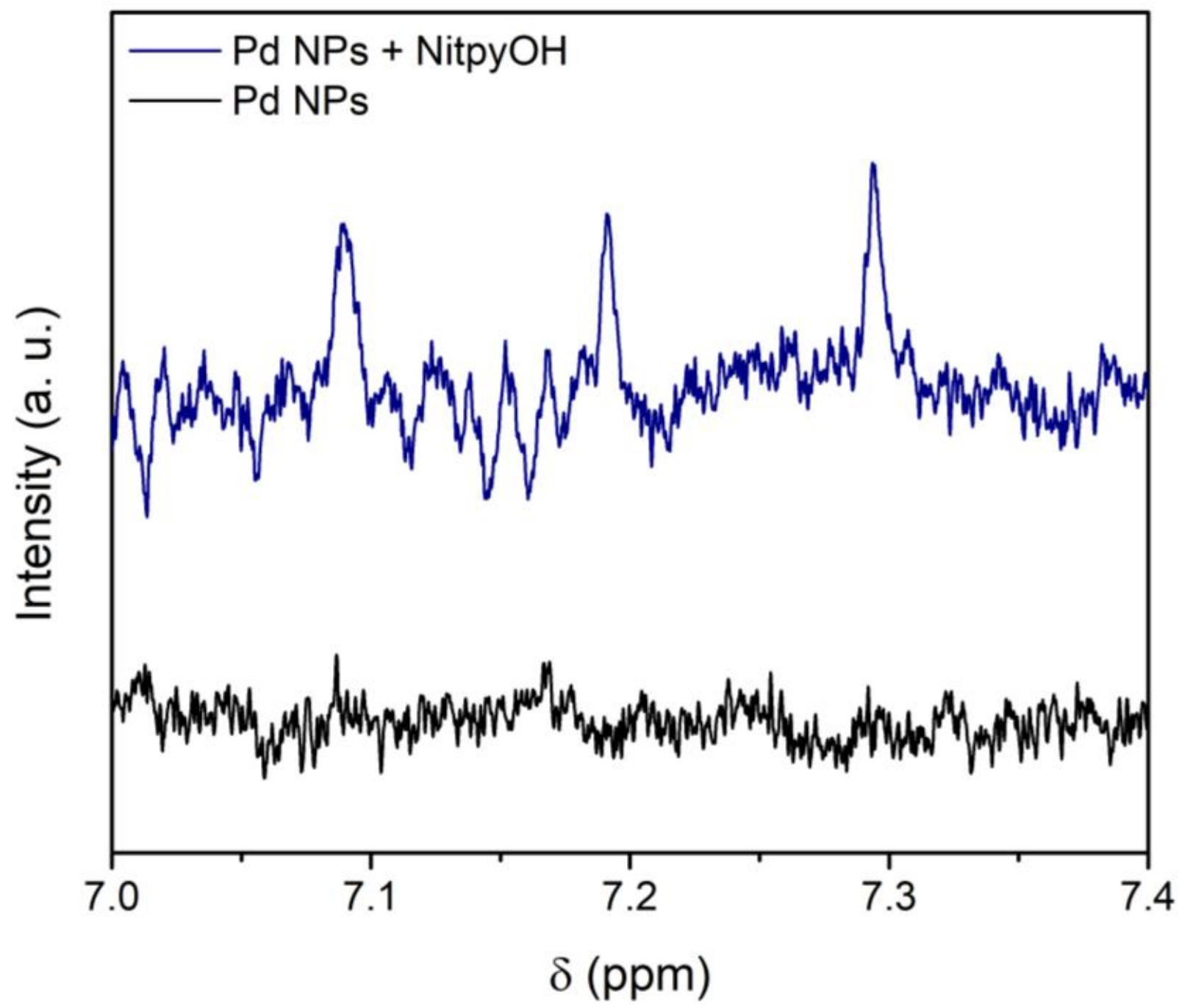

Figure S30. NMR spectra for the production of ammonia with Pd nanoparticles under a constant purge of H_2 and N_2 with and without NitpyOH as catalyst.

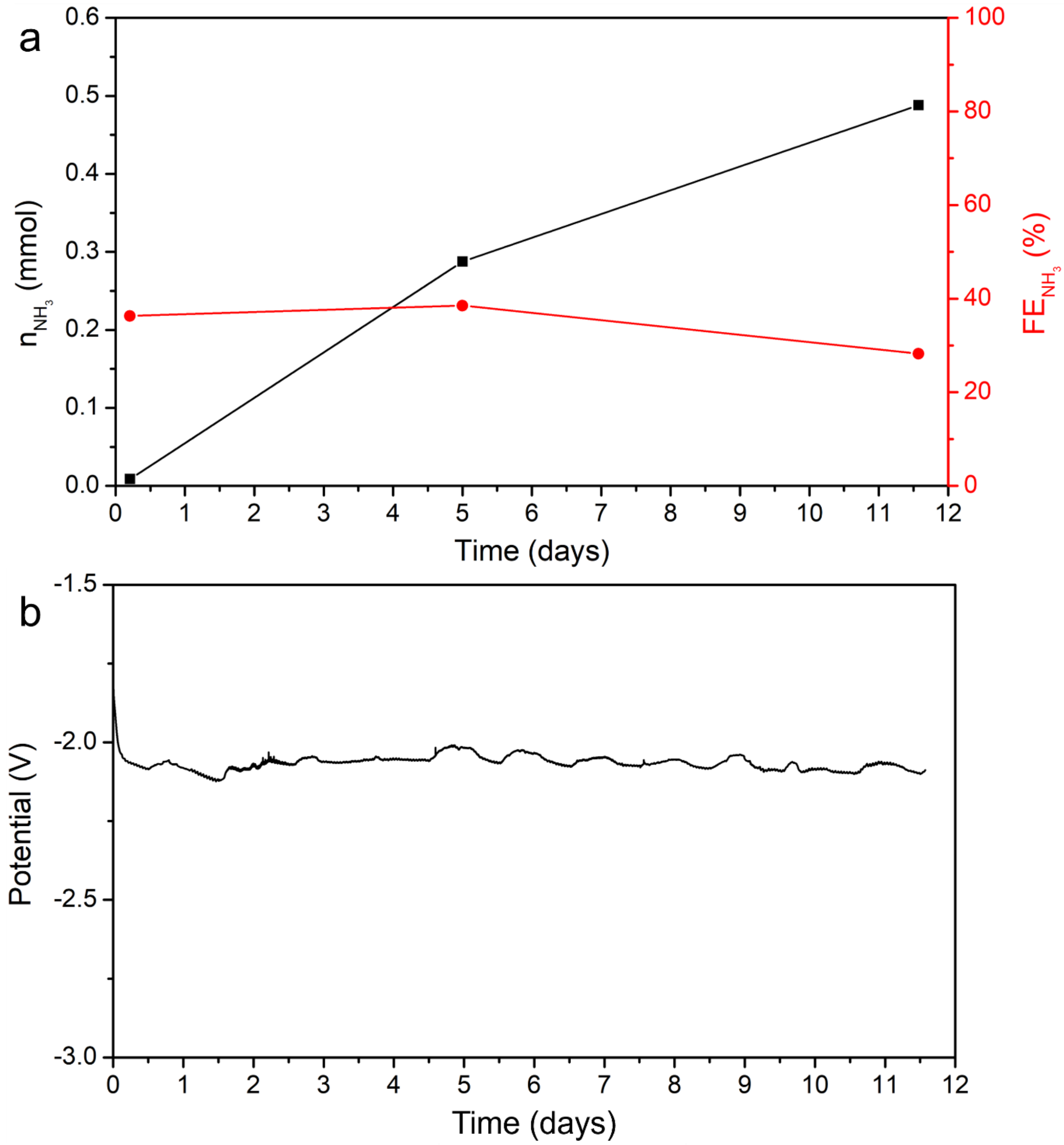

Figure S31. Stability measurement showing the FE and moles of ammonia produced (a) and chronopotentiometry (b) after extended electrolysis at an applied current of -1 mA for 11 days and 12 h generating a TON of 16.

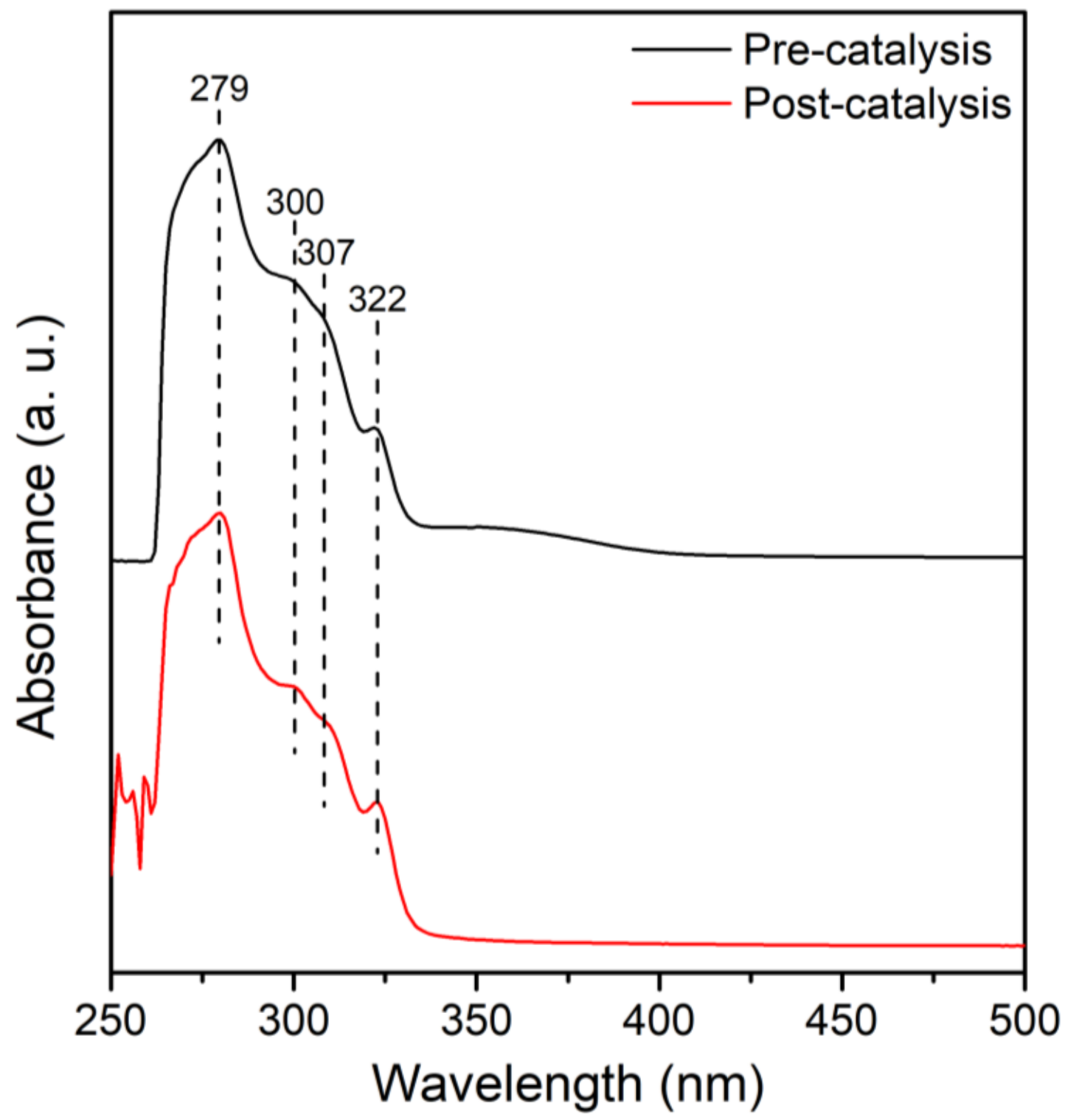

Figure S32. UV-Vis of NitpyOH taken before and after 5 h of electrolysis.

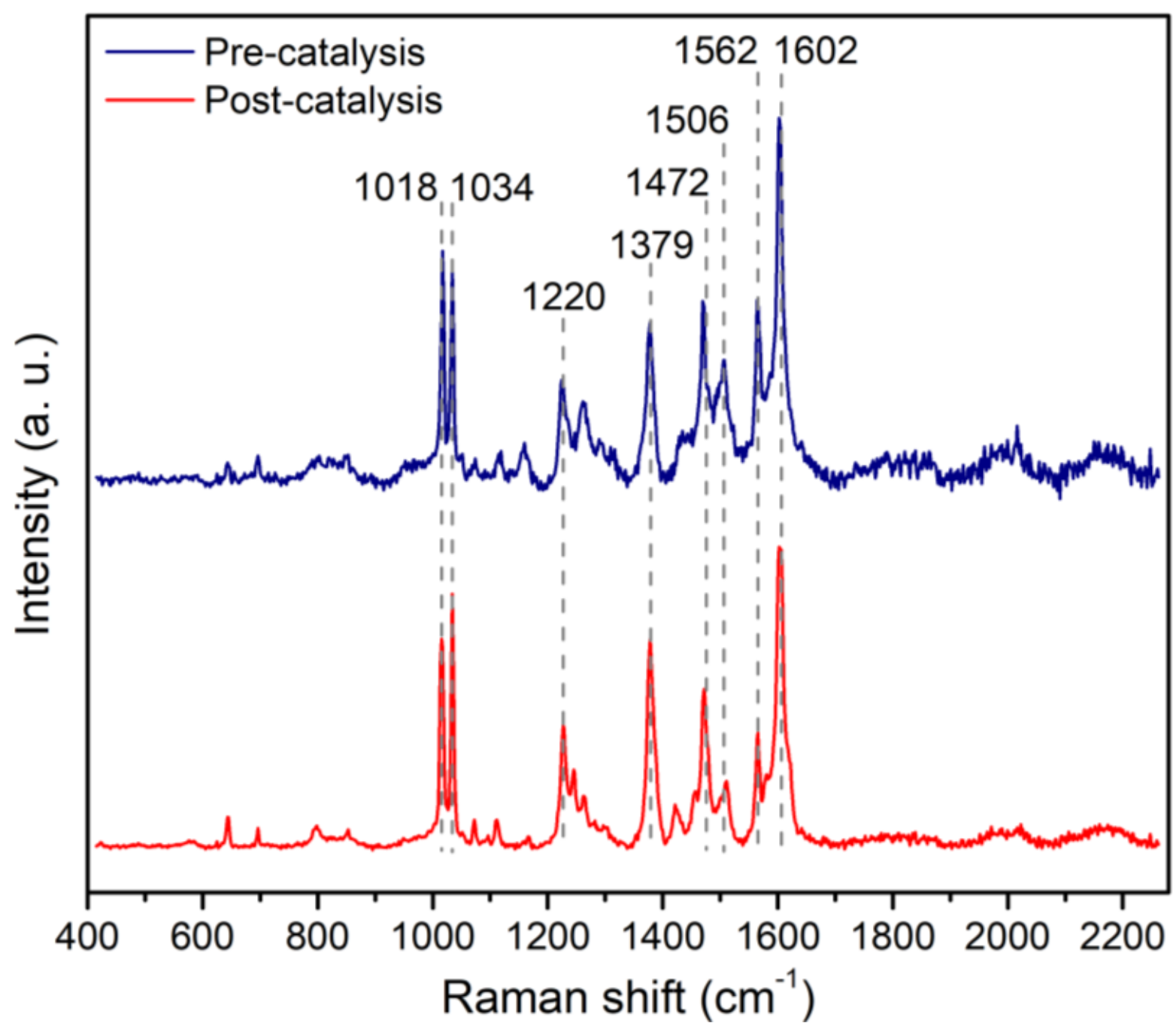

Figure S33. Raman spectra of NitpyOH taken before and after 5 h of electrolysis at -1 mA.

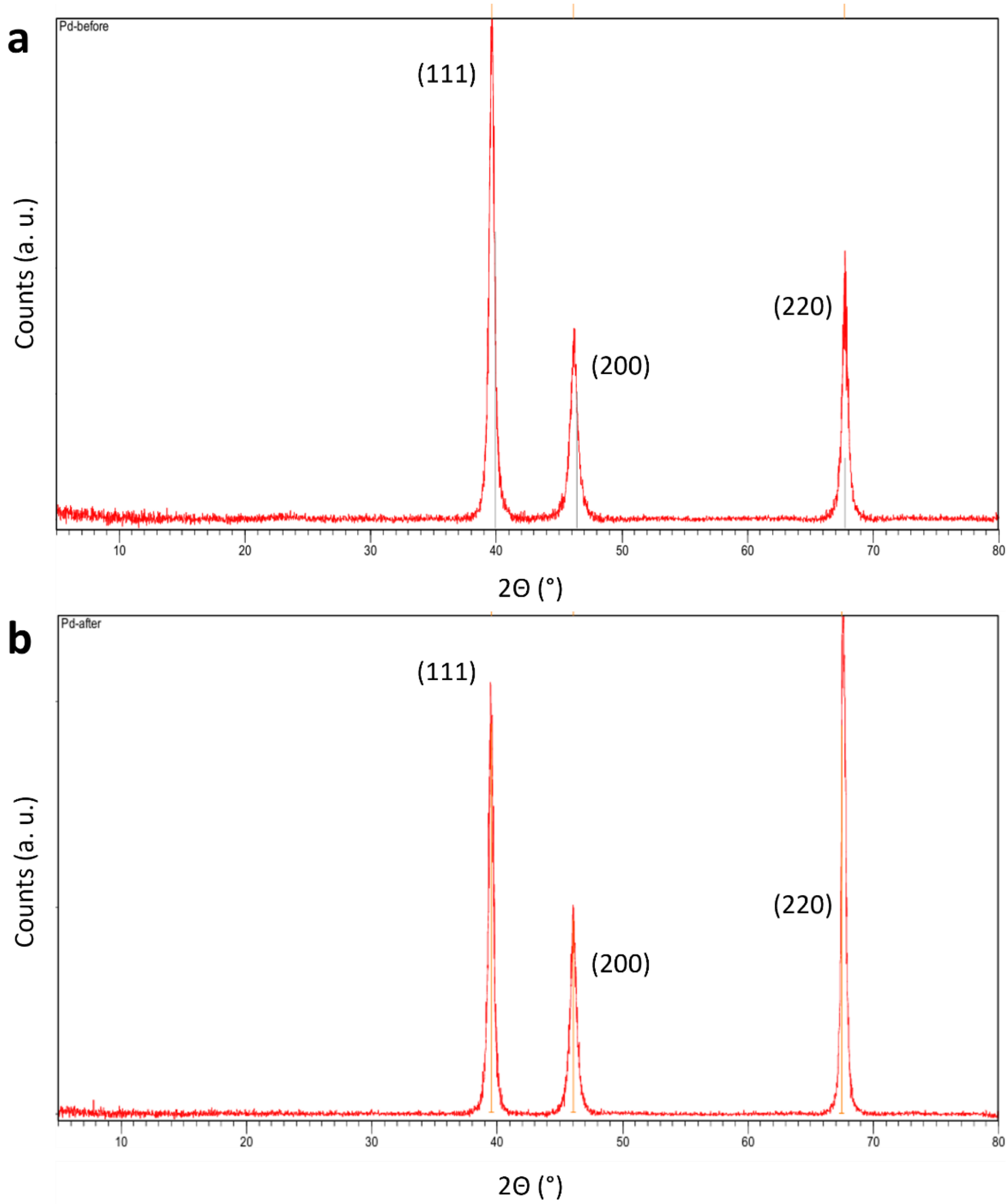

Figure S34. XRD pattern of the Pd membrane, (a) freshly prepared and (b) after catalysis at -1 mA for 5 h, showing the presence of only Pd nanoparticles and no detectable formation of Ni or NiO nanoparticles.

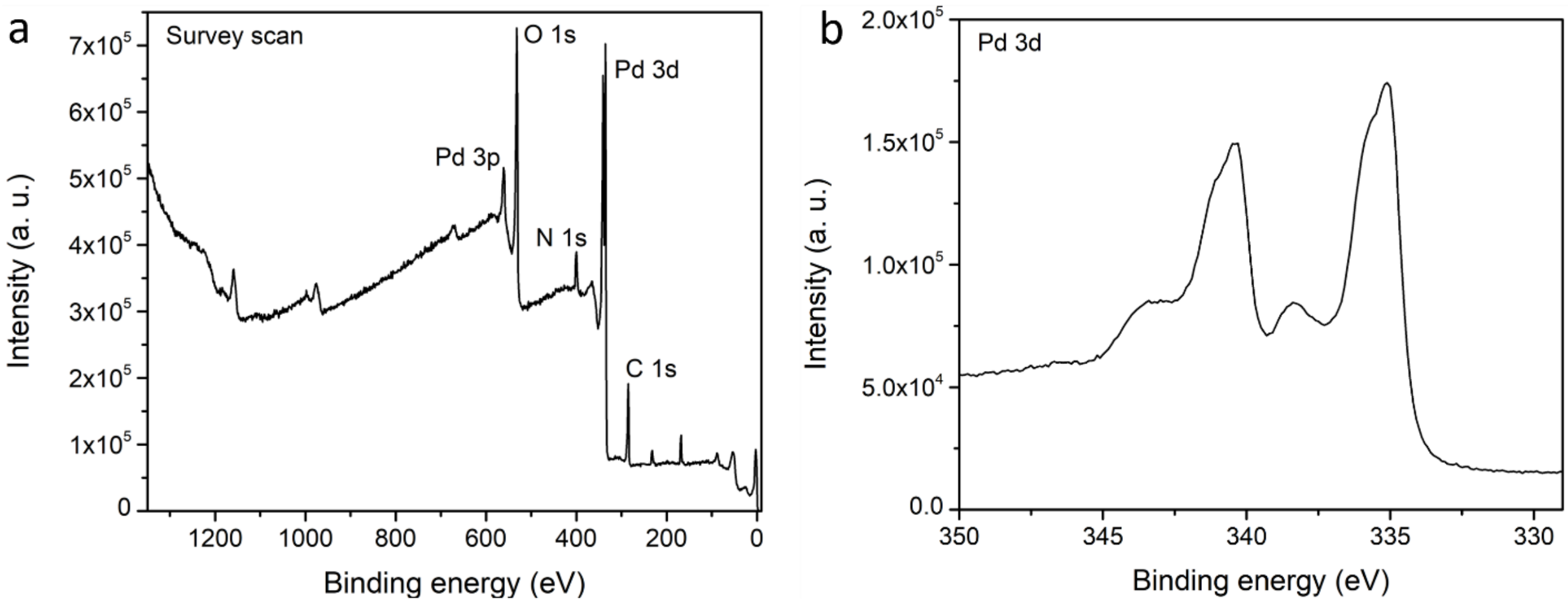

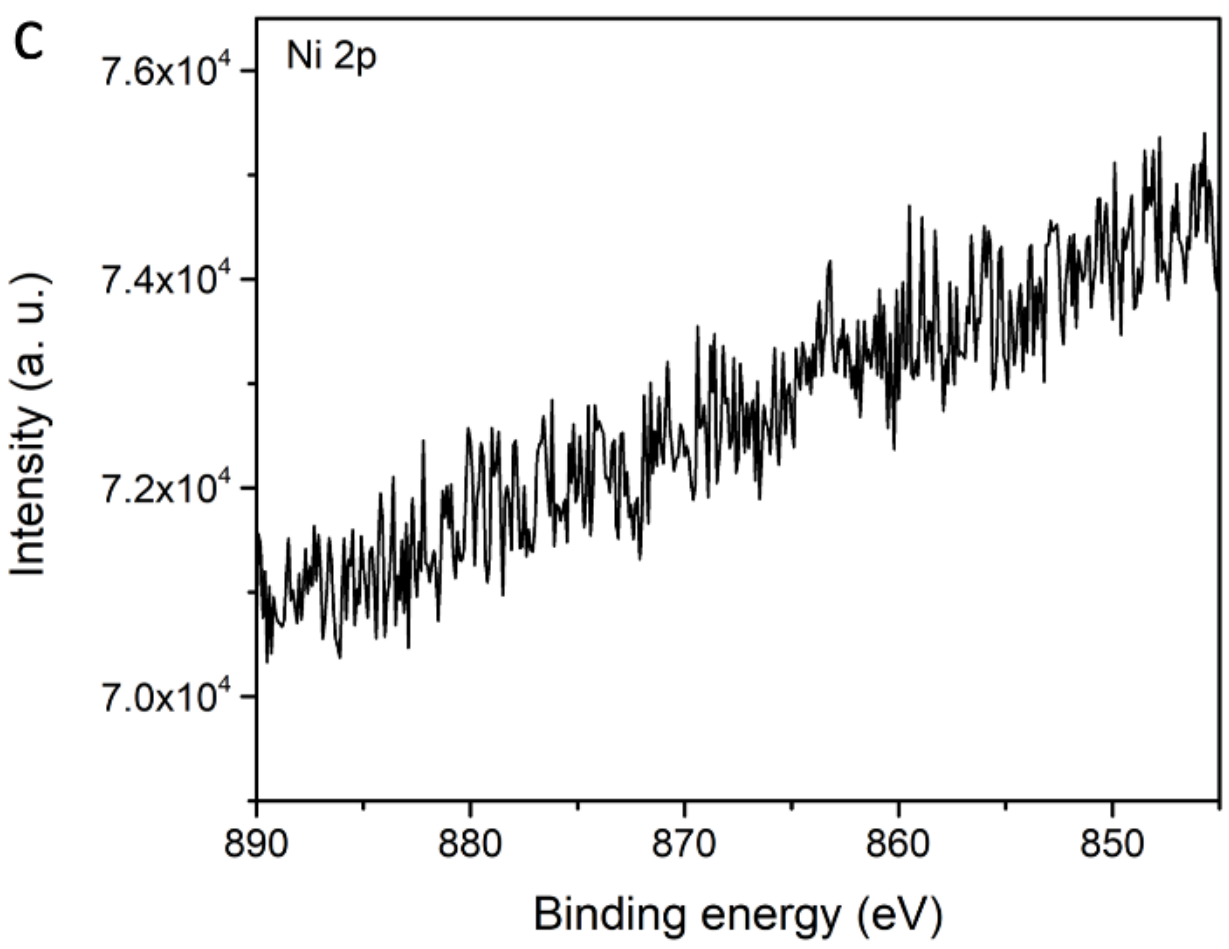

d

Element	Concentration (mg L^{-1})
Ni	0.072
Pd	Saturated detector response

*ICP limit of detection is 0.1 mg L^{-1}

Figure S35. XPS characterization of a palladium membrane following N_2RR with 1 mM NitpyOH at −1 mA for 5 h: (a) survey spectrum, (b) Pd 3d region, and (c) Ni 2p region. (d) ICP-OES analysis of elements detected following aqua regia digestion of the palladium membrane.

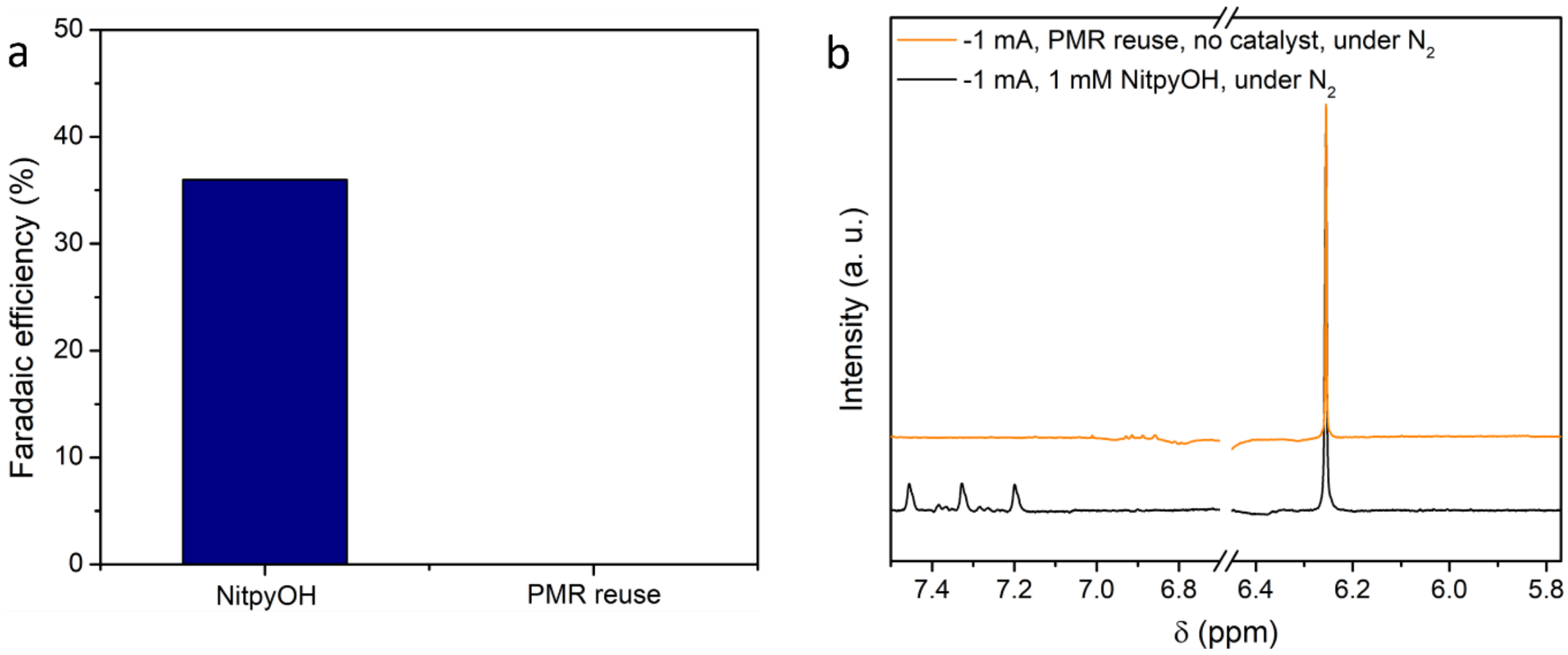

Figure S36. Faradaic efficiency (a) and ^{1}H NMR spectra (b) for ammonia production from dinitrogen using a palladium membrane reactor with 1 mM NitpyOH at an applied current of -1 mA and using the same palladium membrane reactor after 5 h of electrolysis with NitpyOH in fresh DMF without catalyst.

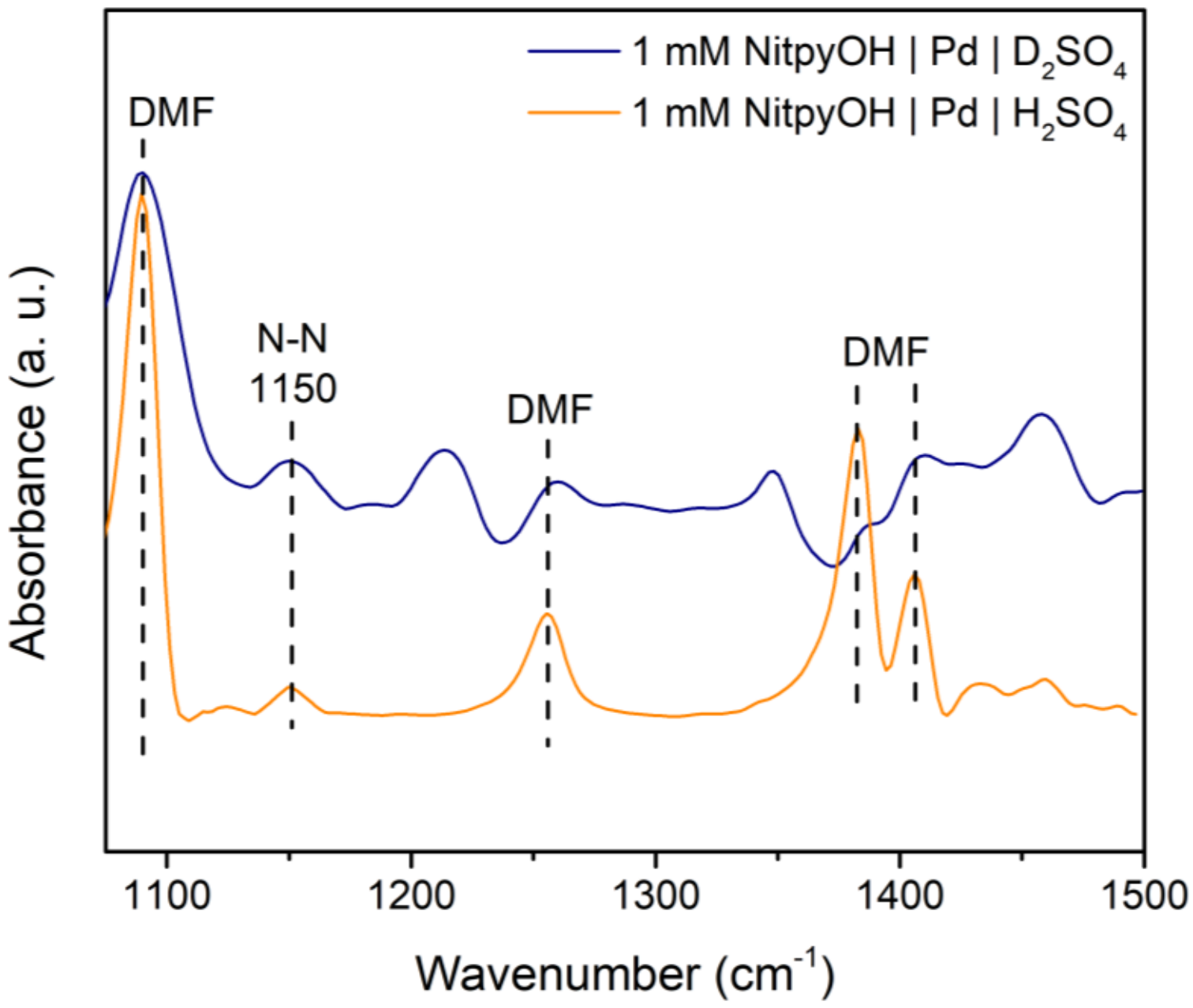

Figure S37. Comparison of *in situ* FTIR spectra in the lower-frequency region during N_2RR using 0.5 M H_2SO_4 and D_2SO_4 with a palladium membrane reactor and NitpyOH catalyst at an applied current of -1 mA.

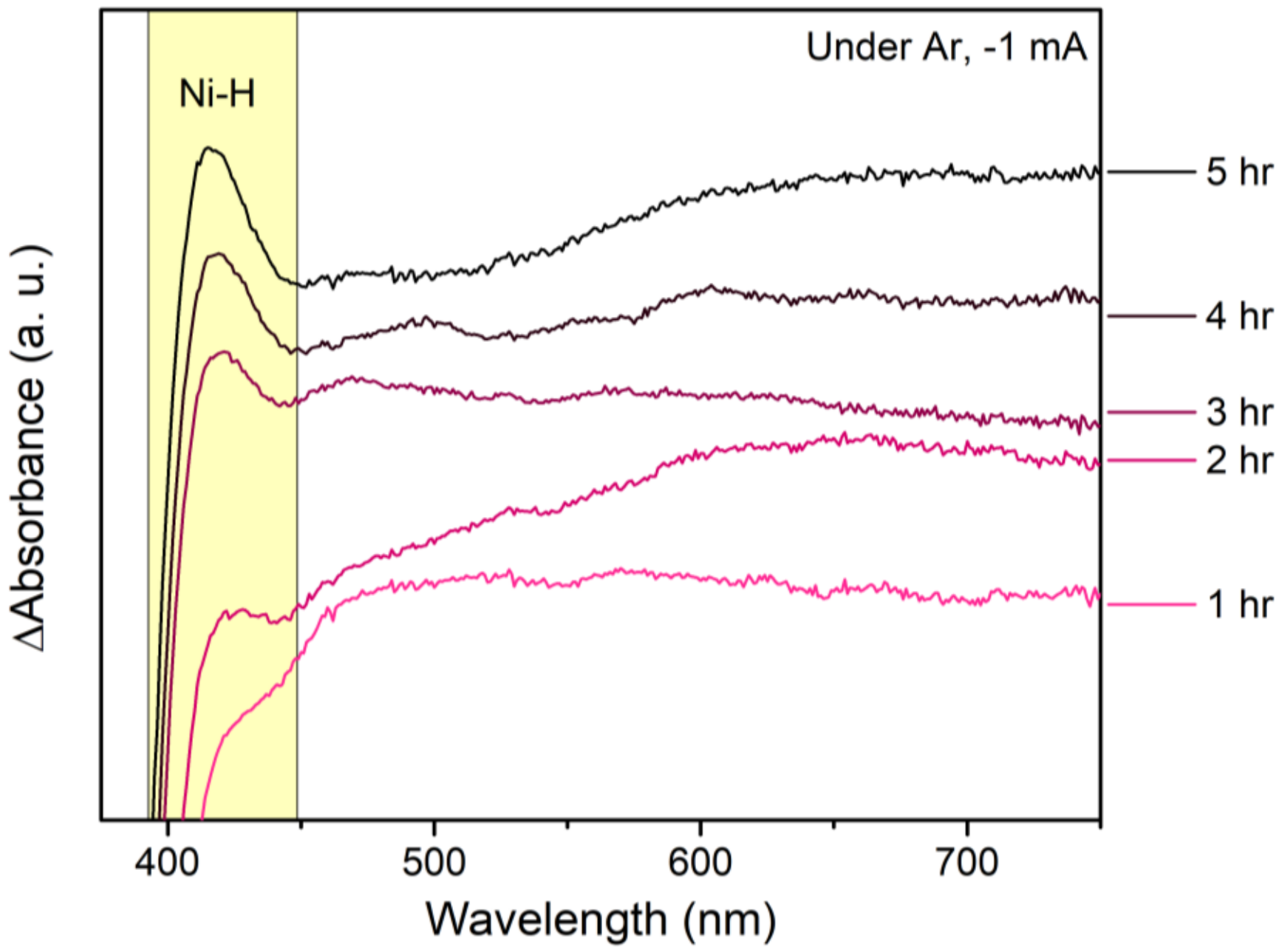

Figure S38. UV-Vis spectral changes of NitpyOH after 5 h of electrolysis under argon atmosphere at a constant current of -1 mA.

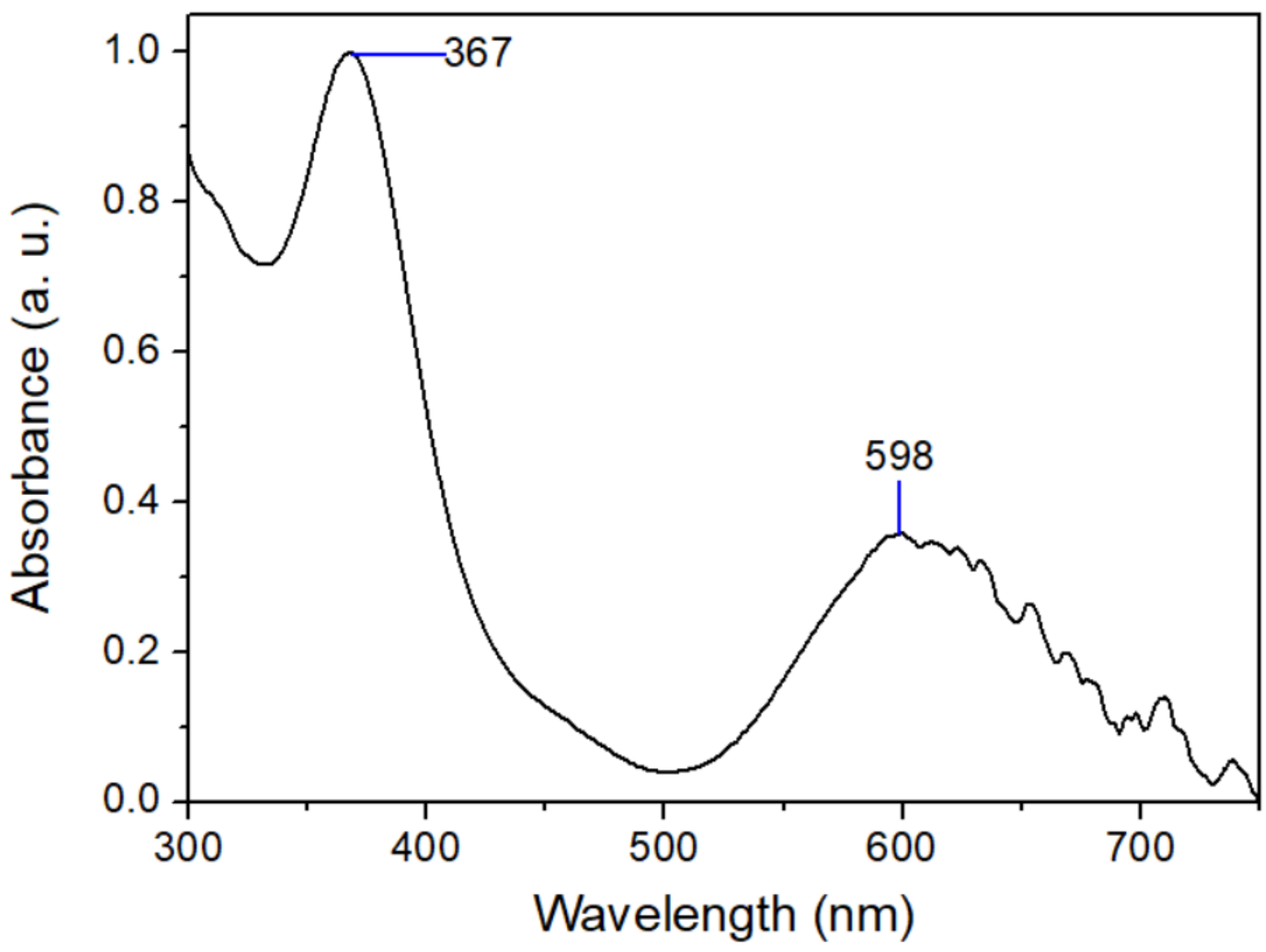

Figure S39. UV-Vis spectra of $Ni(NH_3)_6Cl_2$ in DMF.

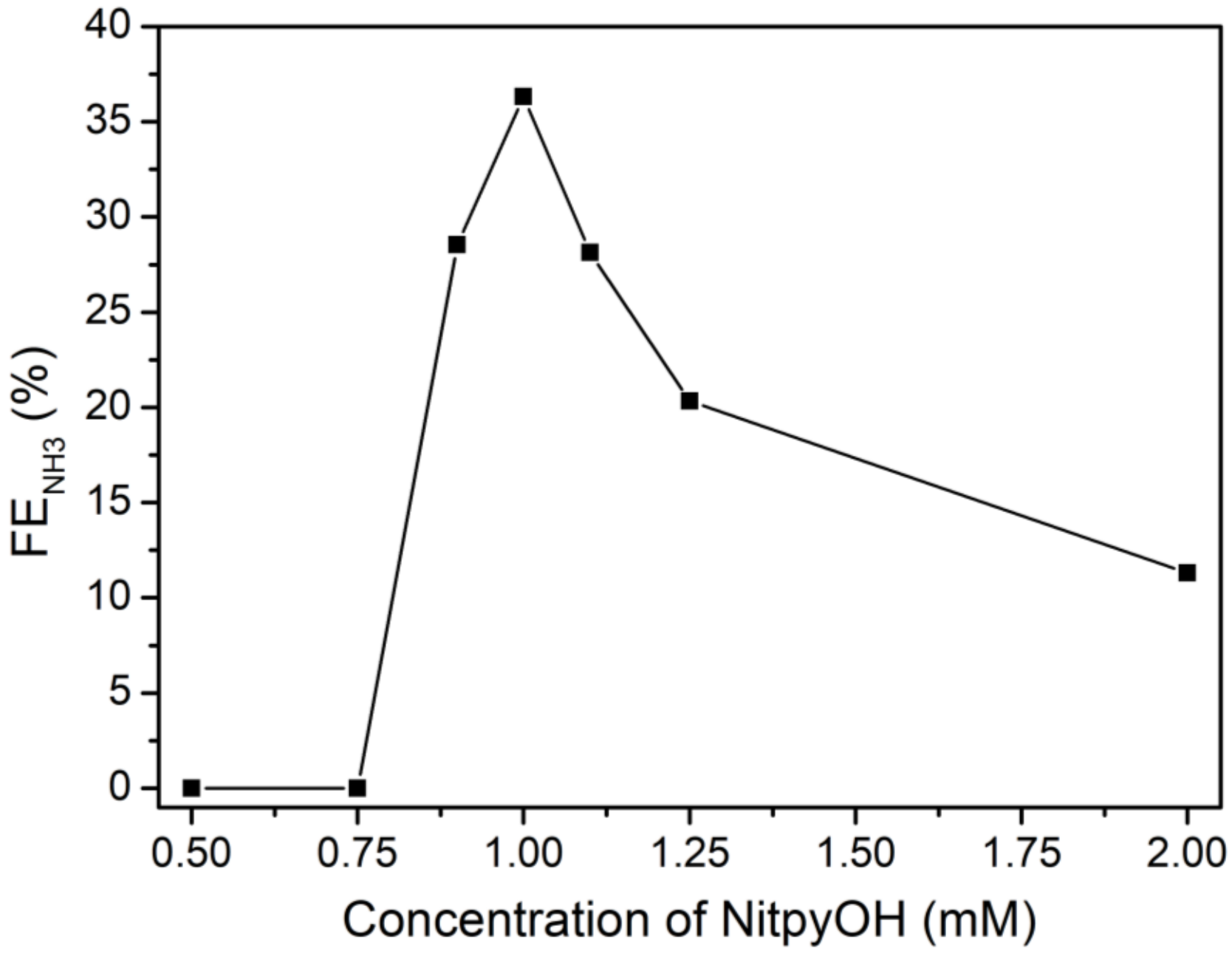

Figure S40. Faradaic efficiency for the production of ammonia at various concentrations of NitpyOH incorporated into a palladium membrane reactor at applied current of -1 mA.

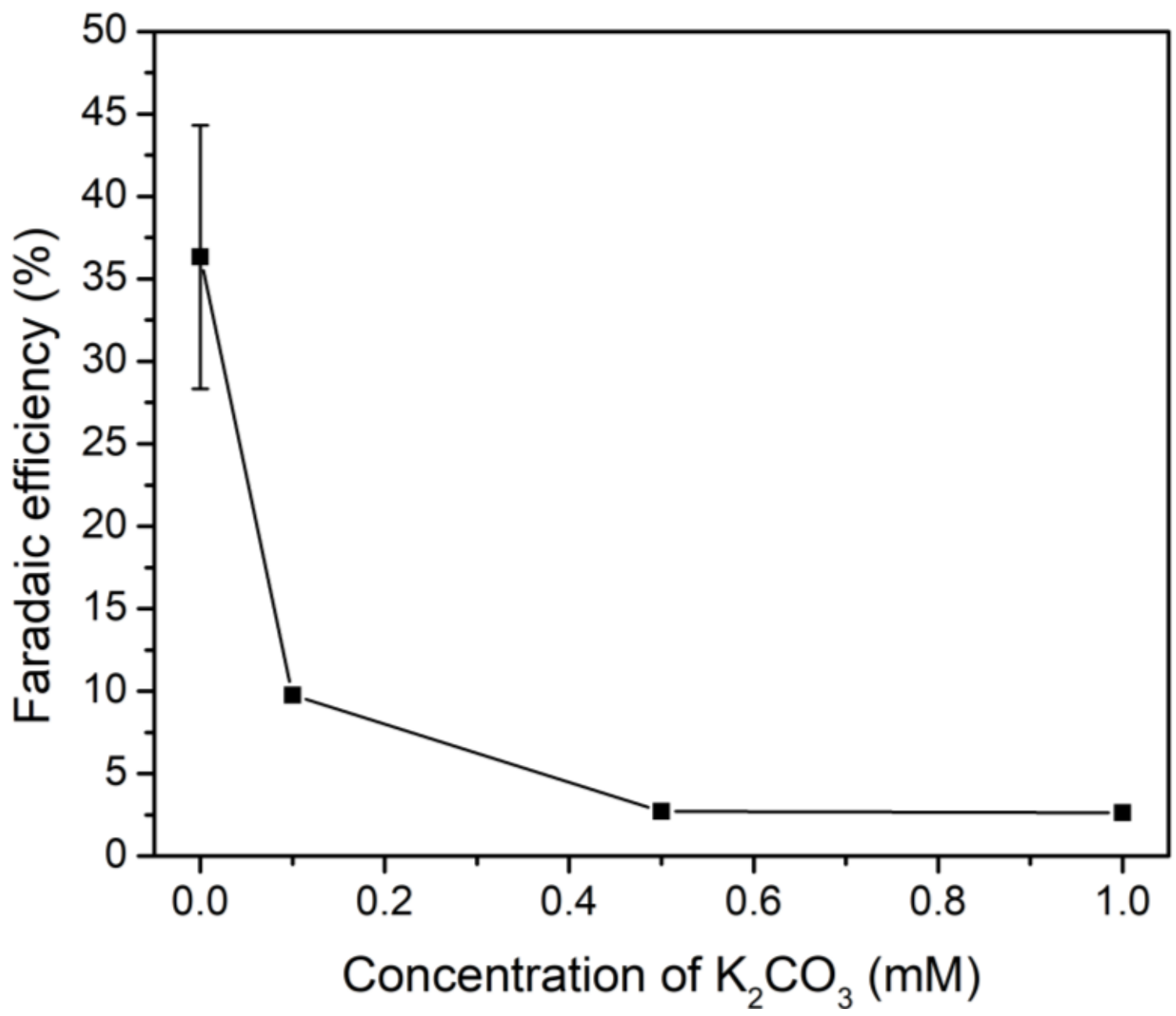

Figure S41. Faradaic efficiency for ammonia production via N_2RR at various concentrations of K_2CO_3 in DMF using 1 mM NitpyOH, held at -1 mA for 5 h.

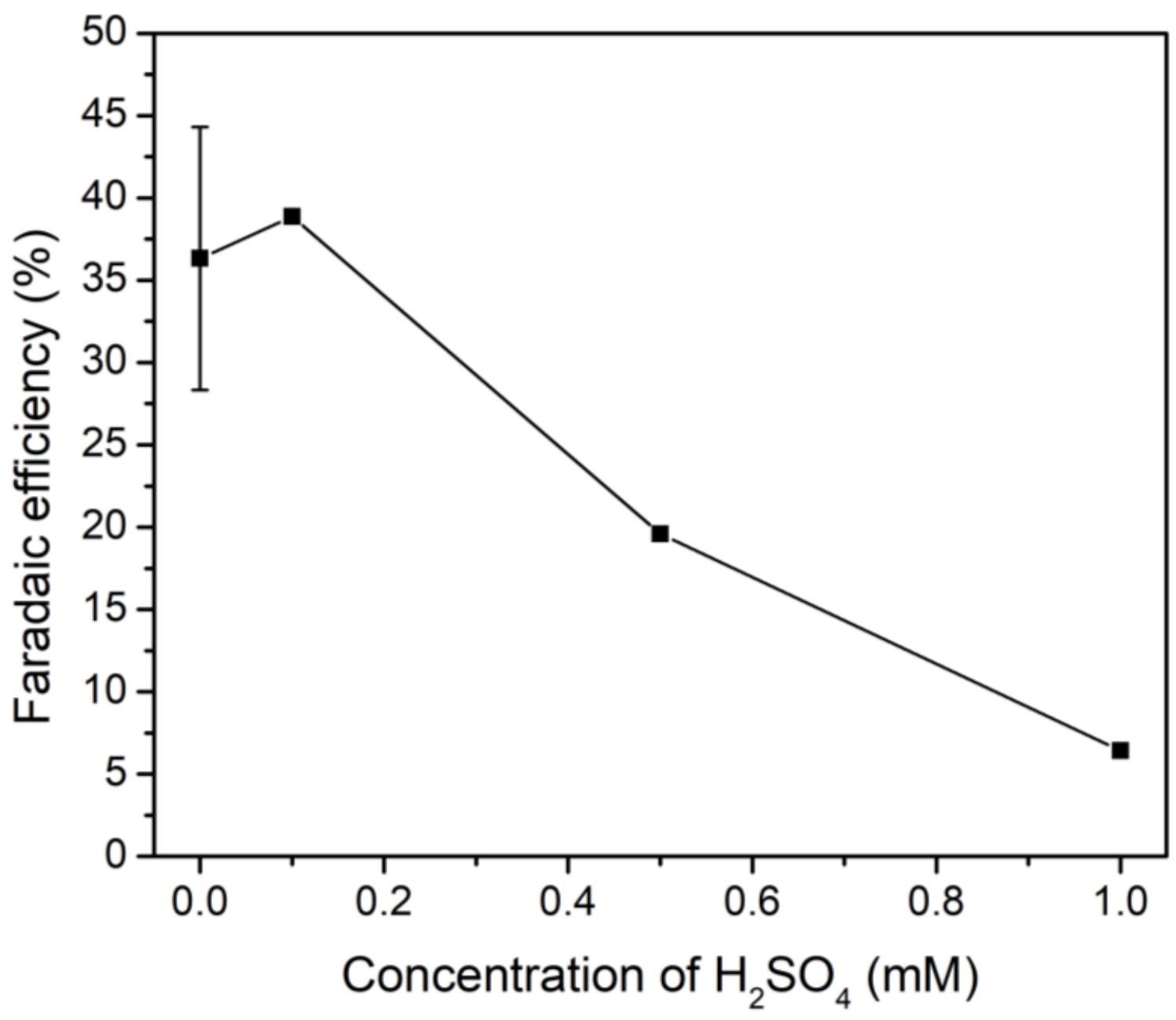

Figure S42. Faradaic efficiency for ammonia production via N_2RR at various concentrations of H_2SO_4 in DMF using 1 mM NitpyOH, held at -1mA for 5 h.

Table S1. Table of Orbital Energies of HOMO and LUMO, energy difference of the HOMO-LUMO gap and Hirshfeld charges of the Ni centre for the respective NitpyR complexes.

	-H	-OH	$-NH_2$	$-CF_3$	-OMe
E_{HOMO} (eV)	-10.17	-10.03	-9.77	-10.34	-9.94
E_{LUMO} (eV)	-6.15	-5.92	-5.65	-6.44	-5.83
$\Delta E_{HOMO-LUMO}$ (eV)	4.01	4.11	4.12	3.89	4.11
Hirshfeld charges	+0.106 (Ni)	+0.103 (Ni)	+0.099 (Ni)	+0.109 (Ni)	+0.102 (Ni)

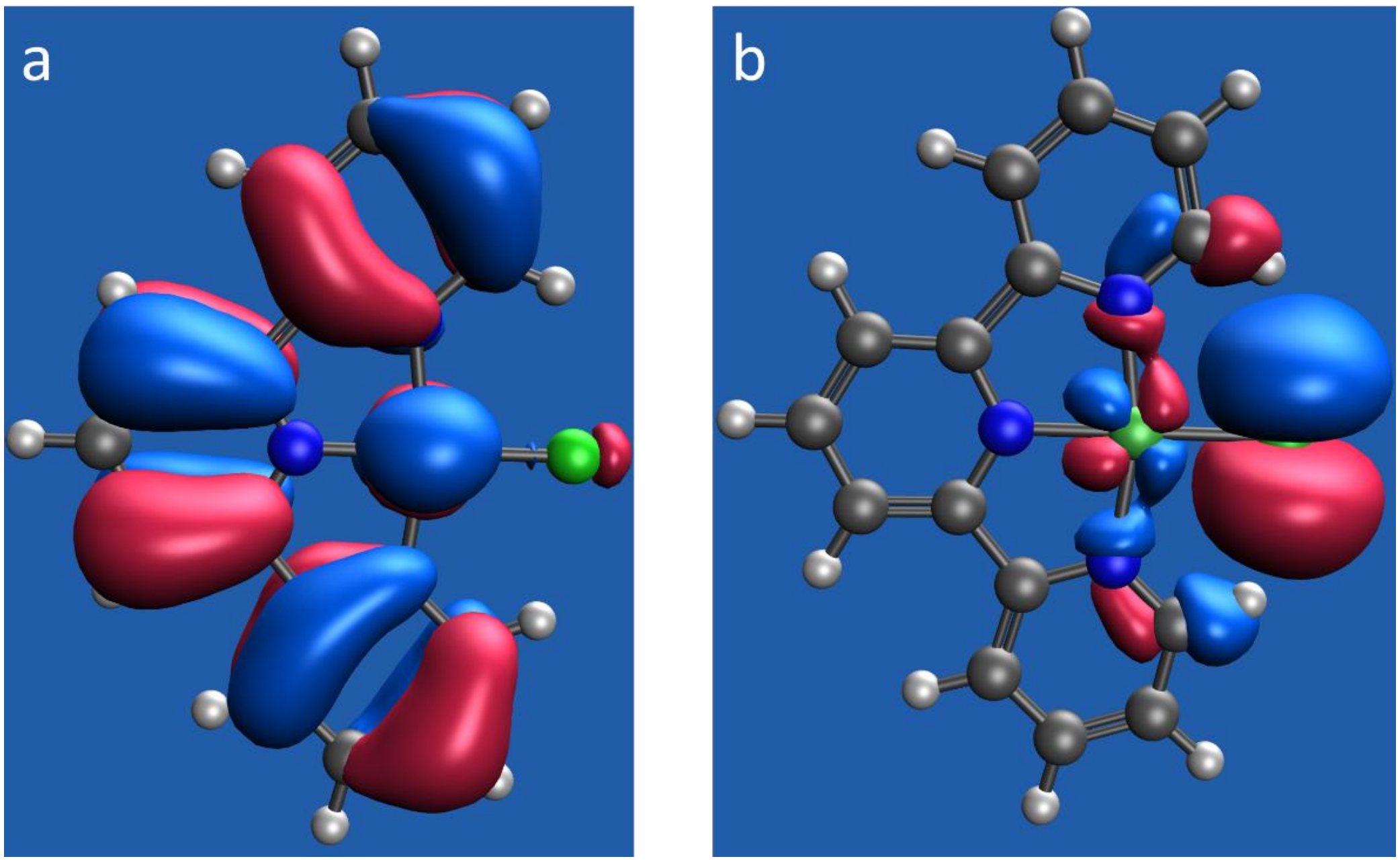

Figure S43. Calculated HOMO (a) and LUMO (b) of the NitpyH complex.

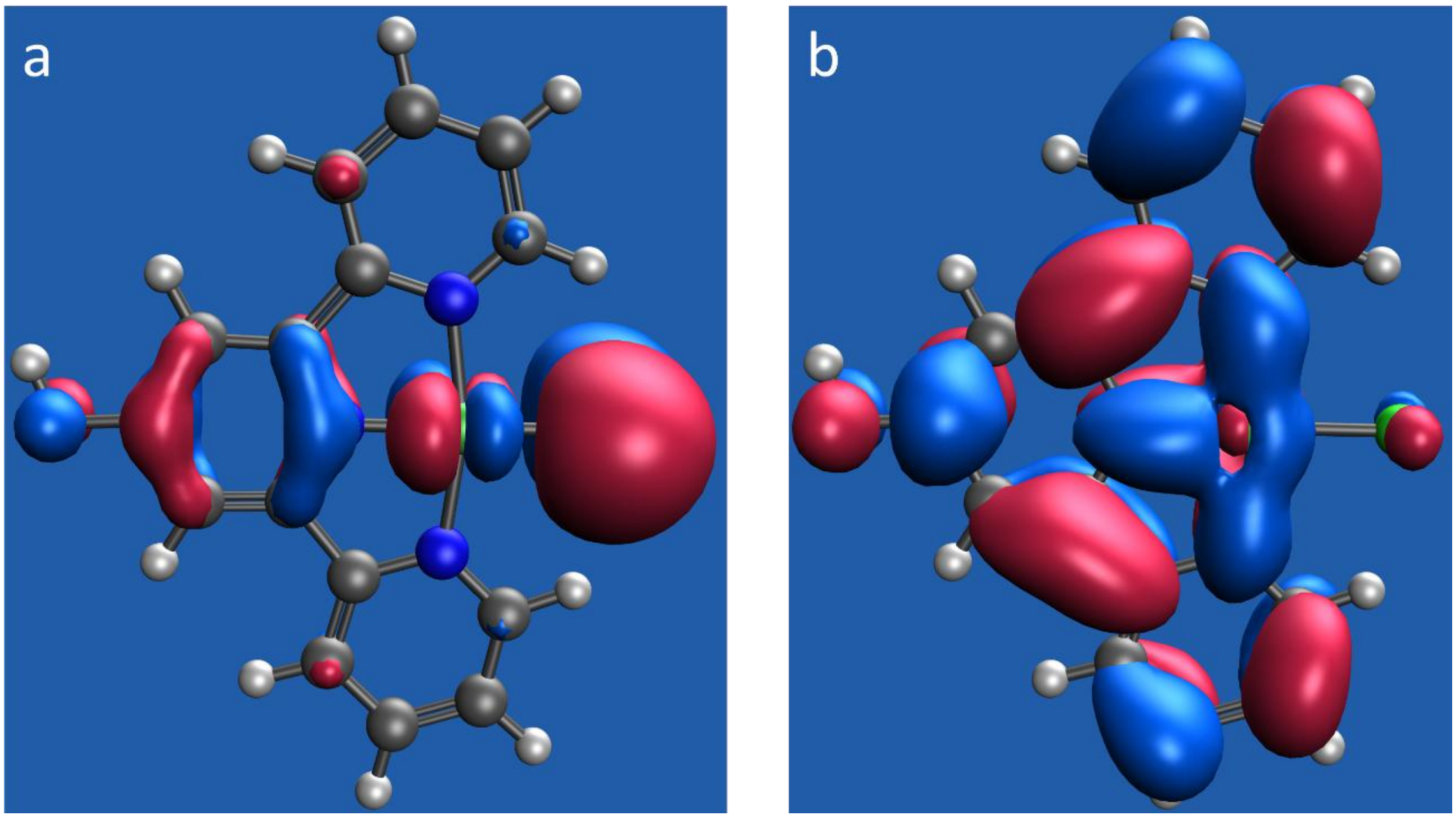

Figure S44. Calculated HOMO (a) and LUMO (b) of the NitpyOH complex.

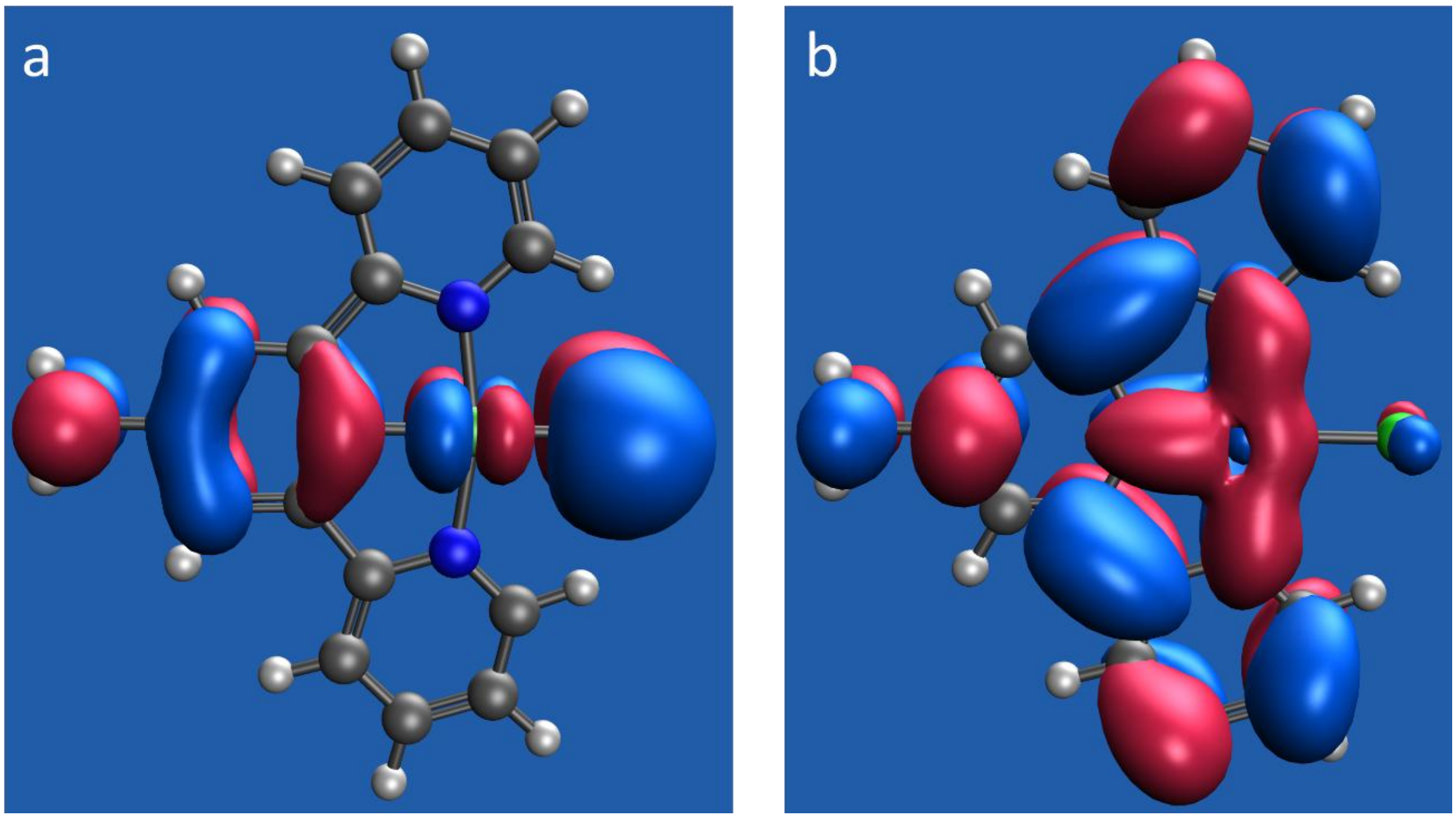

Figure S45. Calculated HOMO (a) and LUMO (b) of the NitpyNH_2 complex.

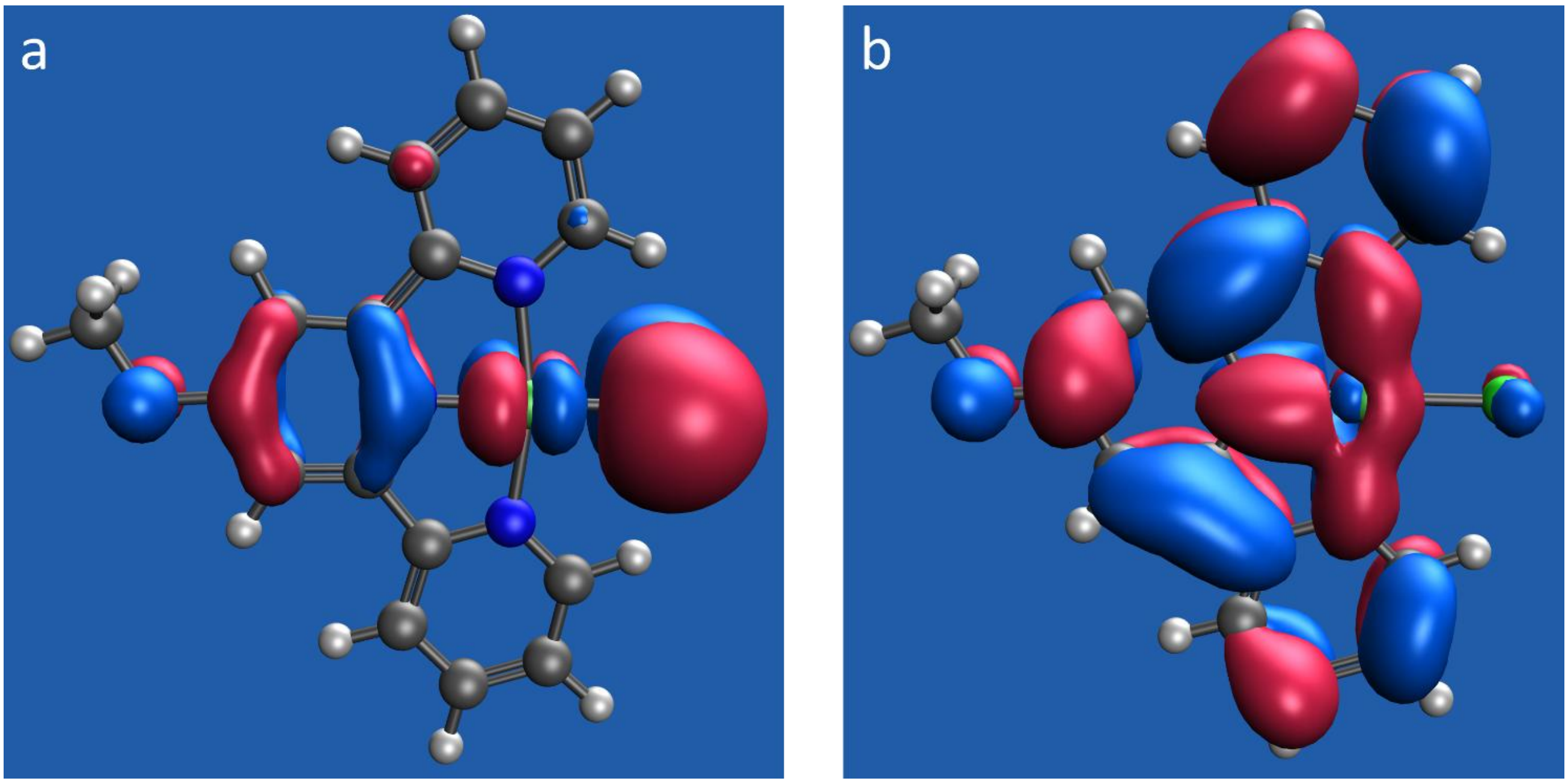

Figure S46. Calculated HOMO (a) and LUMO (b) of the NitpyOMe complex.

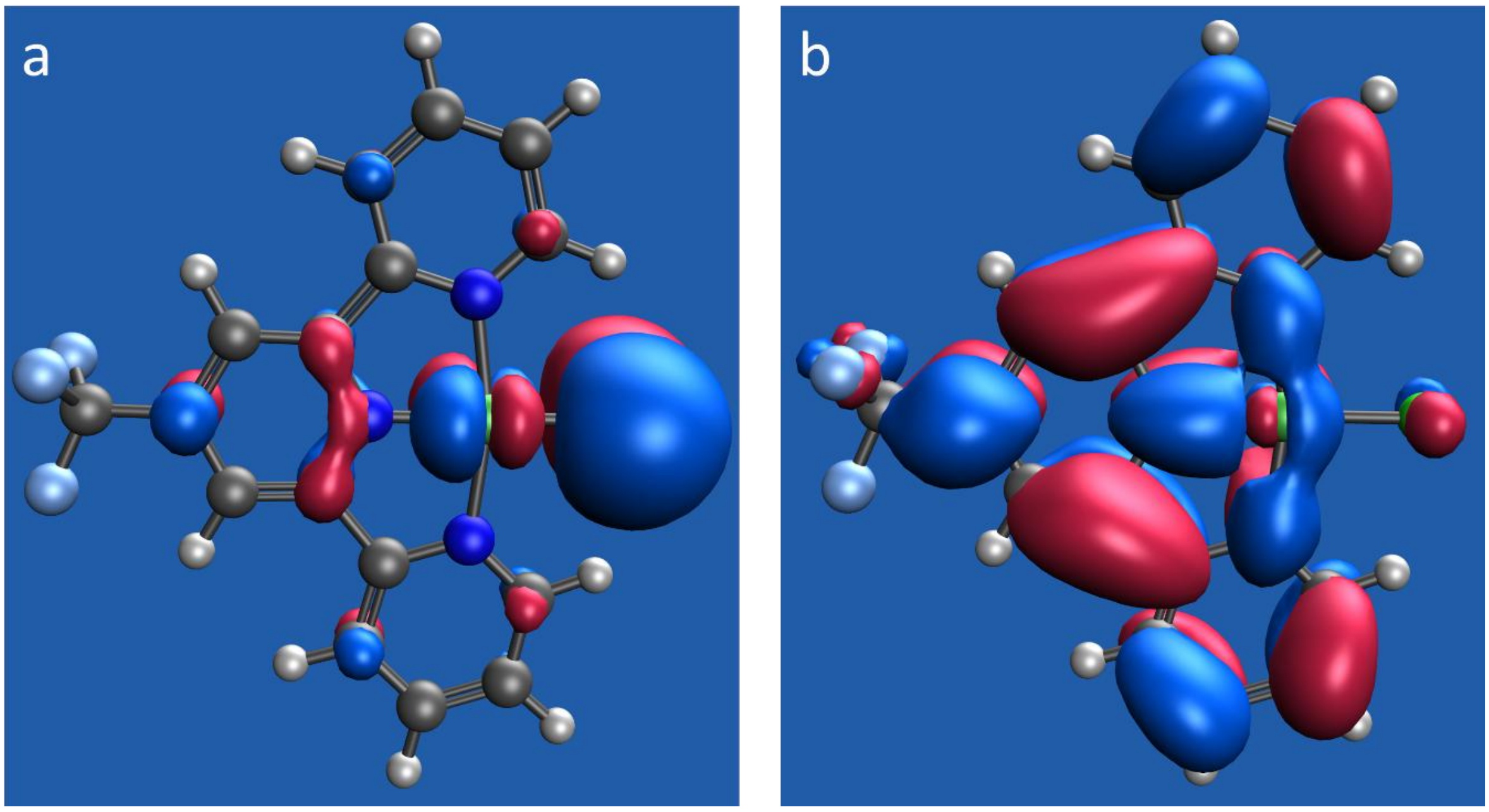

Figure S47. Calculated HOMO (a) and LUMO (b) of the $NitpyCF_3$ complex.

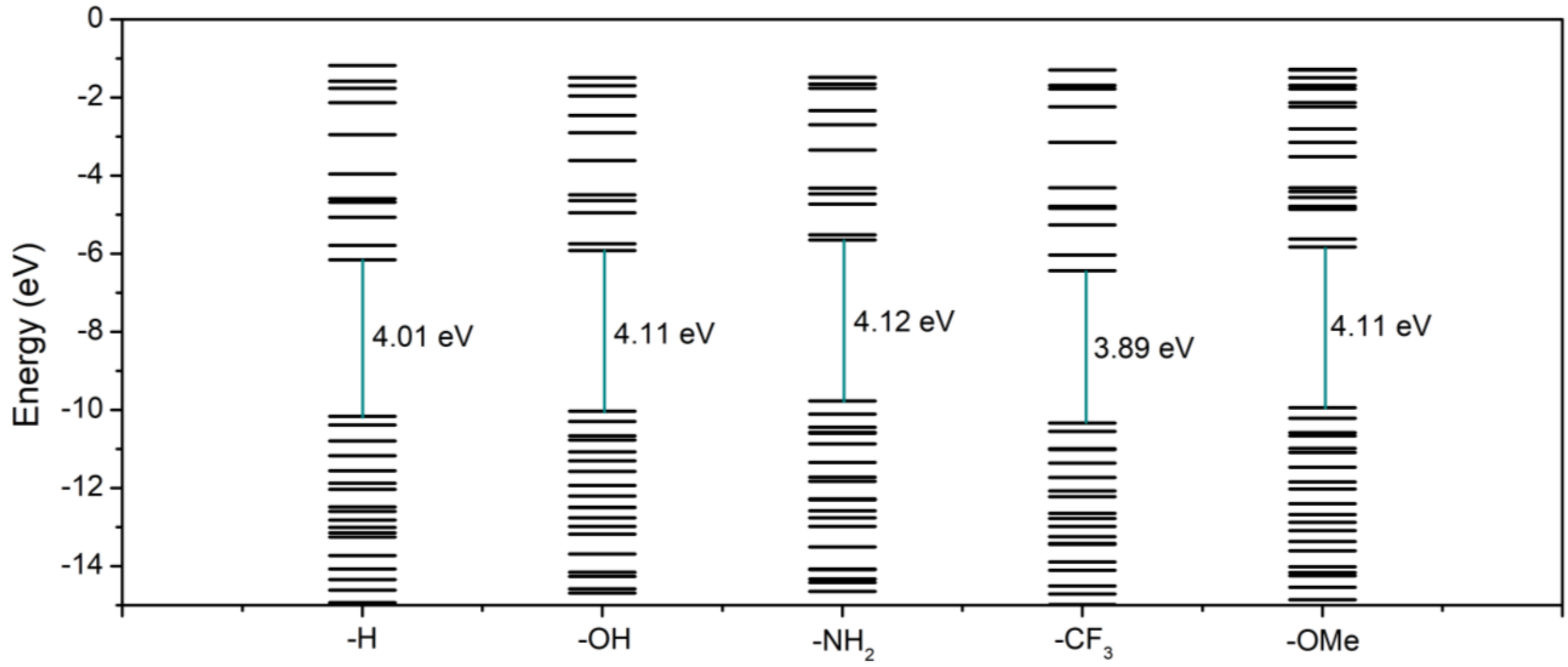

Figure S48. Calculated orbital energy-level diagram for the five NitpyR complexes. The HOMO-LUMO gaps are indicated by blue lines.

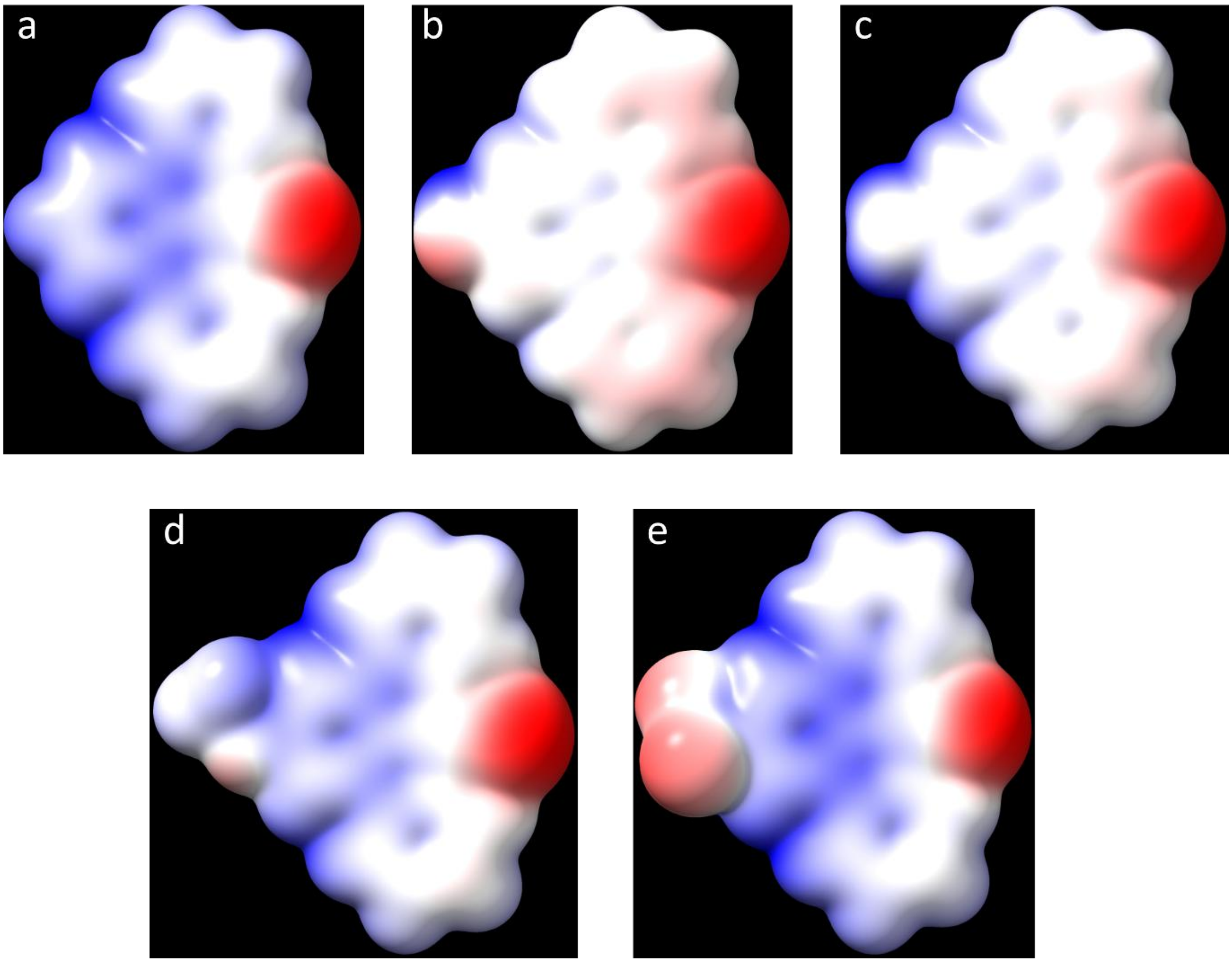

Figure S49. Electrostatic potential mapping on the electron density for NitpyH (a), NitpyOH (b), $NitpyNH_2$ (c), NitpyOMe (d) and $NitpyCF_3$ (e). Red indicates electron-richer and blue electron-poorer areas.

1: Nitpy, [Ni ($C_{15}H_{11}N_3$)Cl(H_2O)$_2$]Cl·H_2O, diaqua-chloro-(2,2':6',2''-terpyridyl)-nickel(II) chloride monohydrate[1]
2: NitpyNH2, ([Ni ($C_{15}H_{12}N_4$)Cl(H_2O)$_2$]Cl·2H_2O, diaqua-chloro-(4'-amino-2,2':6',2''-terpyridil)-nickel(II) chloride dihydrate
3: NitpyOH, [Ni ($C_{15}H_{11}ON_3$)(H_2O)$_3$]Cl_2·H_2O, triaqua-(4'-hydroxo-2,2':6',2''-terpyridyl)-nickel(II) dichloride monohydrate
4: NitpyOCH3, [Ni ($C_{16}H_{13}ON_3$)Cl(H_2O)$_2$]Cl·2H_2O, diaqua-chloro-(4'-methoxy-2,2':6',2''-terpyridyl)-nickel(II) chloride dihydrate
5: (NitpyOH)$_2$, " (1/2[Ni ($C_{15}H_{11}ON_3$)Cl H_2O]$_2$Cl· CH_3OH· H_2O), aqua-chloro-(4'-hydroxo-2,2':6',2''-terpyridyl)-nickel(II) chloride hydrate methanol
6: (NitpyOCF3)$_2$, (1/2[Ni ($C_{16}H_{10}F_3N_3$)Cl(H_2O)]$_2$Cl · SO(CH_3)$_2$), aqua-chloro-(4'-triflouromethoxy-2,2':6',2''-terpyridyl)-nickel(II) dimethylsoulfoxide

Single-crystal X-ray structure determination

The crystal data of our crystal refinements are given in **tables 2, and 3**.
The crystal structure of 1 is already known[1]. Single crystal intensity data was obtained for **1** with a green cube-shaped single crystal of dimensions 0.22 x 0.21 x 0.20 mm^3, for **2** with a light blue plate-shaped single crystal of dimensions 0.050 x 0.130 x 0.185 mm^3, for **3** with a light blue plate-shaped single crystals of dimensions 0.044 x 0.082 x 0.128 mm^3, for **4** with a light green plate-shaped single crystals of dimensions 0.020 x 0.066 x 0.110 mm^3, for **5** with a pale blue prism shaped crystal of the dimensions 0.048 x 0.060 x 0.140 mm^3 and for **6** with a pale green plate shaped crystal of the dimensions 0.020 x 0.050 x 0.130 mm^3. The diffraction data for **1**, **2, 3, 4,** and **5** were collected on Nonius KappaCCD diffractometer[2][3] at 123(2) K, using graphite monochromated Mo-k$_\alpha$ radiation (λ = 0.71073 Å). 7 was collected on a Bruker D8 Venture diffractometer[4] at 267(2) K, using mirror optics monochromated Cu-k$_\alpha$ from a microfocus sealed X-ray tube (λ = 1.54178 Å). The structures were solved by SHELXT using SHELXS-97[5] and the non-hydrogen atoms were refined by full-matrix least-squares methods using SHELXL-97[6]. All hydrogen atoms were located by difference Fourier synthesis and refined isotropically. For **1,** there is a positional disorder of a chloride ion and a water molecule. For these two positions either the water is on one and the chloride ion on the other and vice versa. The disorder was calculated with a 50% occupation of each of the two positions. For **5,** the structure contains one molecule of methanol and one molecule of water per formula unit. These solvent molecules could not be convergently refined, so they were included in the structure using SQUEEZE[7]. For **6,** the solvent molecule dimethylsulfoxide is disordered into split positions and refined to a ratio of 55.66% to 43.78%. For visualisation and rendering DIAMOND[8] was used.

Crystal data: **1 "**Nitpy" ([Ni ($C_{15}H_{11}N_3$)Cl(H_2O)$_2$]Cl·H_2O), M = 416.92 green colour, crystal dimensions 0.200 × 0.210 × 0.220 mm^3, triclinic, space group P$\bar{1}$, Z = 2, a = 6.8924(3) Å, b = 9.6022(4) Å, c = 13.65077(6) Å, α = 81.389(3)°, β = 79.355(3)°, γ = 74.389(3)°, V = 850,38(7) Å^3, D_X = 1.628 gcm^{-3}, μ (MoK_α) = 1.474 mm^{-1}, T = 123(2) K, $2\theta_{(max.)}$ = 55°, number of unique data 3690, completeness to θ: 96,1%, R_{int} 0.0277, R_1 [I > 2σ(I)] = 0.0336, $w(R_2)$ all data 0.0813, goodness of fit 1.060, $\Delta\rho$ (max./min.) = 0.286/-0.489 e Å^{-3}.

Crystal data: **2 "**NitpyNH2" ([Ni ($C_{15}H_{12}N_4$)Cl(H_2O)$_2$]Cl·2H_2O), M = 449,96, light blue colour, crystal dimensions 0.050 × 0.130 × 0.185 mm^3, triclinic, space group P$\bar{1}$, Z = 2, a = 7.0321(9) Å, b = 10.8781(14) Å, c = 13.4818(13) Å, α = 97.903(9)°, β = 104.747(8)°, γ = 108.523(6)°, V = 918,5(2) Å^3,

D_X = 1.627 gcm^{-3}, μ (MoK_α) = 1.3690 mm^{-1}, T = 123(2) K, transmission factors (min/max) 0.9385/0.8128, analytical absorption correction[7], 2θ($_{max.}$) = 55°, number of unique data 4289, completeness to θ: 99,6%, R_{int} 0.1248, R_1 [I > 2σ(I)] = 0.0459, $w(R_2)$ all data 0.1056, goodness of fit 0.970, $\Delta\rho$ (max./min.) = 0.415/-0.419 e Å^{-3}.

Crystal data: **3** **"**NitpyOH" ([Ni ($C_{15}H_{11}ON_3$)(H_2O)$_3$]Cl_2·H_2O), M = 450,94, light blue colour, crystal dimensions 0.044 × 0.083 × 0.128 mm^3, triclinic, space group P$\bar{1}$, Z = 2, a = 8.1958(5) Å, b = 8.3913(5) Å, c = 13.7000(7) Å, α = 91.348(4)°, β = 100.463(3)°, γ = 99.844(3)°, V = 911,44(3) Å^3, D_X = 1.463 gcm^{-3}, μ (MoK_α) = 1.389 mm^{-1}, T = 123(2) K, 2θ($_{max.}$) = 55°, number of unique data 3442, completeness to θ: 86,1%, R_{int} 0.0410, R_1 [I > 2σ(I)] = 0.0398, $w(R_2)$ all data 0.0937, goodness of fit 0.984, $\Delta\rho$ (max./min.) = 0.416/-0.632 e Å^{-3}.

Crystal data: **4** **"**NitpyOCH3" ([Ni ($C_{16}H_{13}ON_3$)Cl(H_2O)$_2$]Cl·2H_2O), M = 464,97, light green colour, crystal dimensions 0.020 × 0.066 × 0.110 mm^3, triclinic, space group P$\bar{1}$, Z = 2, a = 6.8354(3) Å, b = 11.6550(8) Å, c = 13.1546(8) Å, α = 106.021(3)°, β = 103.751(4)°, γ = 96.191(4)°, V = 961,4(1) Å^3, D_X = 1.606 gcm^{-3}, μ (MoK_α) = 1.320 mm^{-1}, T = 123(2) K, 2θ($_{max.}$) = 55°, number of unique data 3486, completeness to θ: 83,6%, R_{int} 0.0789, R_1 [I > 2σ(I)] = 0.0451, $w(R_2)$ all data 0.1020, goodness of fit 0.963, $\Delta\rho$ (max./min.) = 0.461/-0.446 e Å^{-3}.

Crystal data: **5** **"(**NitpyOH)$_2$" (1/2[Ni ($C_{15}H_{11}ON_3$)Cl H_2O]$_2$Cl· CH_3OH· H_2O), M = 437,94, light blue colour, crystal dimensions 0.048 × 0.060 × 0.140 mm^3, monoclinic, space group C2/c, Z = 8, a = 13.976(2) Å, b = 15.772(2) Å, c = 17.281(3) Å, β = 109.951(9)°, V = 3580,6(11) Å^3, D_X = 1.664 gcm^{-3}, μ (MoK_α) = 1.406 mm^{-1}, T = 123(2) K, 2θ($_{max.}$) = 50.52°, number of unique data 3021, completeness to θ: 92,9 %, R_{int} 0.1752, R_1 [I > 2σ(I)] = 0.0782, $w(R_2)$ all data 0.1624, goodness of fit 0.891, $\Delta\rho$ (max./min.) = 0.49/-0.57 e Å^{-3}.

Crystal data: **6** **"(**NitpyOCF3)$_2$" (1/2[Ni ($C_{16}H_{10}F_3N_3$)Cl(H_2O)]$_2$Cl · SO(CH_3)$_2$), M = 527.02, light green colour, crystal dimensions 0.020 × 0.050 × 0.130 mm^3, triclinic, space group P$\bar{1}$, Z = 2, a = 8.064(2) Å, b = 10.424(3) Å, c = 13.866(4) Å, α = 100.28(2)°, β = 90.67(2)°, γ = 106.63(2)°, V = 1096,5(6) Å^3, D_X = 1.590 gcm^{-3}, μ (CuK_α) = 4.826 mm^{-1}, T = 267(2) K, 2θ($_{max.}$) = 135,5°, number of unique data 4695, completeness to θ: 100 %, R_{int} 0.2278, R_1 [I > 2σ(I)] = 0.0736, $w(R_2)$ all data 0.2170, goodness of fit 1.043, $\Delta\rho$ (max./min.) = 0.514/-0.412 e Å^{-3}.

Crystallographic data for the structure determinations presented in this paper have been deposited with the Cambridge Crystallographic Data Centre, CCDC, 12 Union Road, Cambridge CB21EZ, UK (deposition numbers **1**: 1139166[1]; **2**: 2393798; **3**: 2393812; **4**: 2421696; **5**: 2442483; **6**: 2442481). Copies of the data can be obtained free of charge on quoting the depository number CCDC-2007995; E- Mail: deposit@ccdc.cam.ac.uk,
http://www.ccdc.cam.ac.uk)

[1] R. Cortes, M.I. Arriortua, T. Rojo, X. Solans, C. Miravitlles, D. Beltran, Acta Crystallographica, Section C: Crystal Structure Communications, **1985**, 41, 1733,

[2] *Collect data collection software*, Nonius B.V., **1999**

[3] Z. Otwinoski and W. Minor *Processing of X-ry Diffraction Data Collected in OscillationMode*, Methods in Enzymology, Volume 276: Macromolecular Crystallography, part A. p. 307-326, **1997**, C.W. Carter, Jr.&R.M. Sweet, Eds., Academic Press.

[4] Bruker (2012). APEX5, SAINT, SADABS. Bruker AXS Inc., Madison, Wisconsin, USA.
[4] SHELXT: G. M. Sheldrick, *Acta Crystallogr., Sect. A71,* **2015**, 3-8
[5] SHELXL-97: G. M. Sheldrick, *Acta Crystallogr., Sect. A64,* **2015**, 112-122
[6] SQUEEZE: Spek, A. L. (2015). *Acta Cryst.* C**71**, 9-18
[7] DIAMOND: Brandenburg, K. [or Brandenburg, K. & Putz, H., or Brandenburg, K. & Berndt, M.] (1999). *DIAMOND.* Crystal Impact GbR, Bonn, Germany.

Table 2. Crystal data and structure refinement data of **1** Nitpy, **2** NitpyNH2, and **3** NitpyOH.

	**1:** Nitpy	**2:** NitpyNH2	**3:** NitpyOH
Formula sum	$NiC_{15}H_{17}N_3Cl_2O_3$	$NiC_{15}H_{20}N_4Cl_2O_4$	$NiC_{15}H_{19}N_3Cl_2O_5$
Formula sum weight	416.92	449.96	450.94
Space group (no.)	$P\bar{1}$ *(no. 2)*	$P\bar{1}$ *(no. 2)*	$P\bar{1}$ *(no. 2)*
Lattice constants	a = 6.8924(3) Å<br>b = 9.6022(4) Å<br>c = 13.65077(6) Å<br>α = 81.389(3)°<br>β = 79.355(3)°<br>γ = 74.389(3)°<br>V = 850,38(7) Å³	a = 7.0321(9) Å<br>b = 10.8781(14) Å<br>c = 13.4818(13) Å<br>α = 97.903(9)°<br>β = 104.747(8)°<br>γ = 108.523(6)°<br>V = 918.5 (2) Å³	a = 8.1958(5) Å<br>b = 8.3913(5) Å<br>c = 13.7000(7) Å<br>α = 91.348(4)°<br>β = 100.463(3)°<br>γ = 99.844(3)°<br>V = 911,44(3) Å³
Temperature (K)	123 (2)	123 (2)	123 (2)
2 θ range (°)	3.045 – 27.51	2.91 – 27.48	1.91 – 27.48
R_1 / wR_2 (I > 2σ I)[a]	0.0336 / 0.0749	0.0459 / 0.0880	0.0398 / 0.0836
R_1 / wR_2 (all data)	0.0530 / 0.0813	0.1065 / 0.1056	0.0683 / 0.0937
Goodness of fit	1.060	0.970	0.984
Largest diff peak/hole [e Å^{-3}]	0.286/-0.489	0.415/-0.419	0.416/-0.632

[a] $R_1 = \sum||F_o| - |F_c||/\sum|F_o|$, $wR_2 = [\sum w(F_o^2 - F_c^2)^2/\sum w(F_o^2)^2]^{1/2}$

Table 3. Crystal data and structure refinement data of **4** NitpyOCH3, **5** (NitpyOH)2, and **6** (NitpyCF3)2.

	**4:** NitpyOCH3	**5:** (NitpyOH)2	**6**: (NitpyOCF3)2
Formula sum	$NiC_{16}H_{21}N_3Cl_2O_5$	$NiC_{16}H1_{18}N_3Cl_2O_{3,5}$	$NiC_{18}H_{18}N_3F_3Cl_2SO_2$
Formula sum weight	464.97	437.94	527.02
Space group (no.)	$P\bar{1}$ *(no. 2)*	*C 2/c (no. 15)*	$P\bar{1}$ *(no. 2)*
Lattice constants	a = 6.354(3) Å<br>b = 11.6550(8) Å<br>c = 13.1546(8) Å<br>α = 106.021(3)°<br>β = 103.751(4)°<br>γ = 96.191(4)°<br>V = 961,4(1) Å^3	a = 13.676(2) Å<br>b = 15.772(3) Å<br>c = 17.281(3) Å<br>α = 90°<br>β = 109.951(9)°<br>γ = 90°<br>V = 3580,6(11) Å^3	a = 8.064(2) Å<br>b = 10.424(3) Å<br>c = 13.866(4) Å<br>α = 100.28(2)°<br>β = 90.67(2)°<br>γ = 106.63(2)°<br>V = 1096,5(6) Å^3
Temperature (K)	123 (2)	123 (2)	267 (2)
2 θ range (°)	2.46 – 27.48	3.101 – 25.258	6.492 – 135,47
R_1 / wR_2 (I > 2σ I)[a]	0.0451 / 0.0893	0.0782 / 0.1624	0.0736 / 0.1766
R_1 / wR_2 (all data)	0.0833 / 0.1020	0.2250 / 0.2063	0.1450 / 0.2170
Goodness of fit	0.963	0.891	1.043
Largest diff peak/hole [e Å^{-3}]	0.461/-0.446	0.49/-0.57	0.514/-0.412

[a] $R_1 = \sum||F_o| - |F_c||/\sum|F_o|$, $wR_2 = [\sum w(F_o^2 - F_c^2)^2/\sum w(F_o^2)^2]^{1/2}$

1: Nitpy ([Ni ($C_{15}H_{11}N_3$)Cl(H_2O)$_2$]Cl·H_2O)

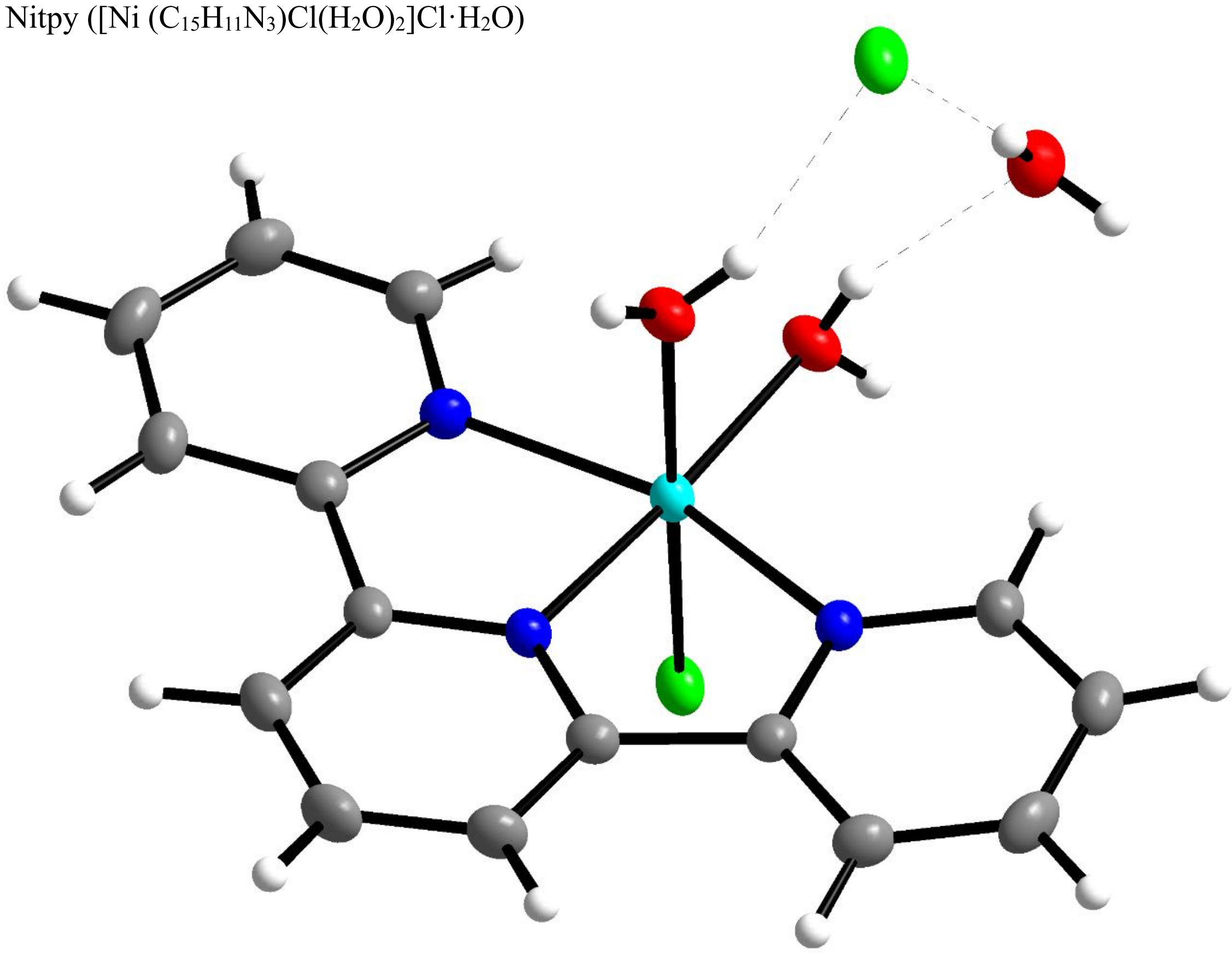

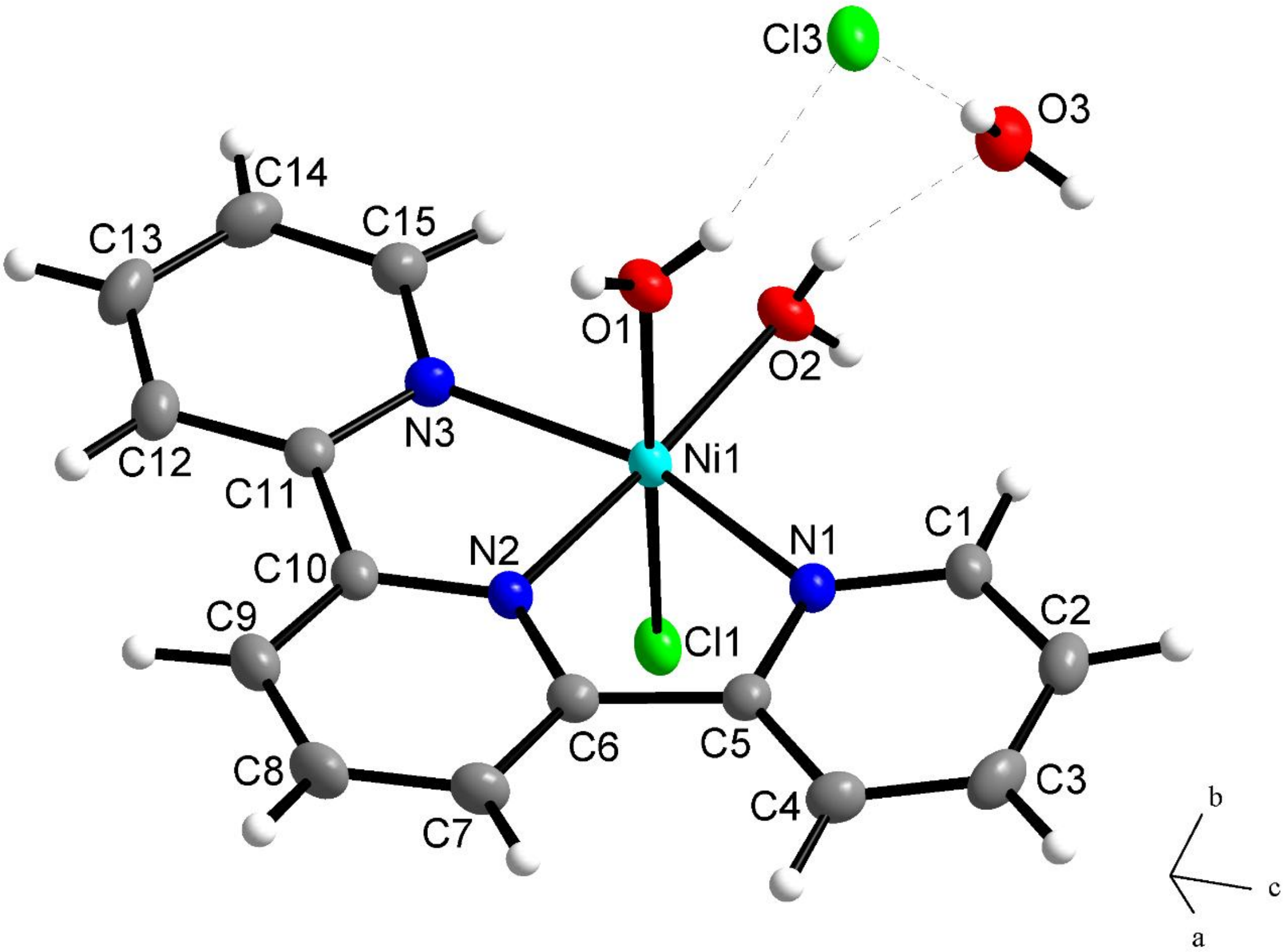

2: NitpyNH2 ($[Ni(C_{15}H_{12}N_4)Cl(H_2O)_2]Cl\cdot 2H_2O$)

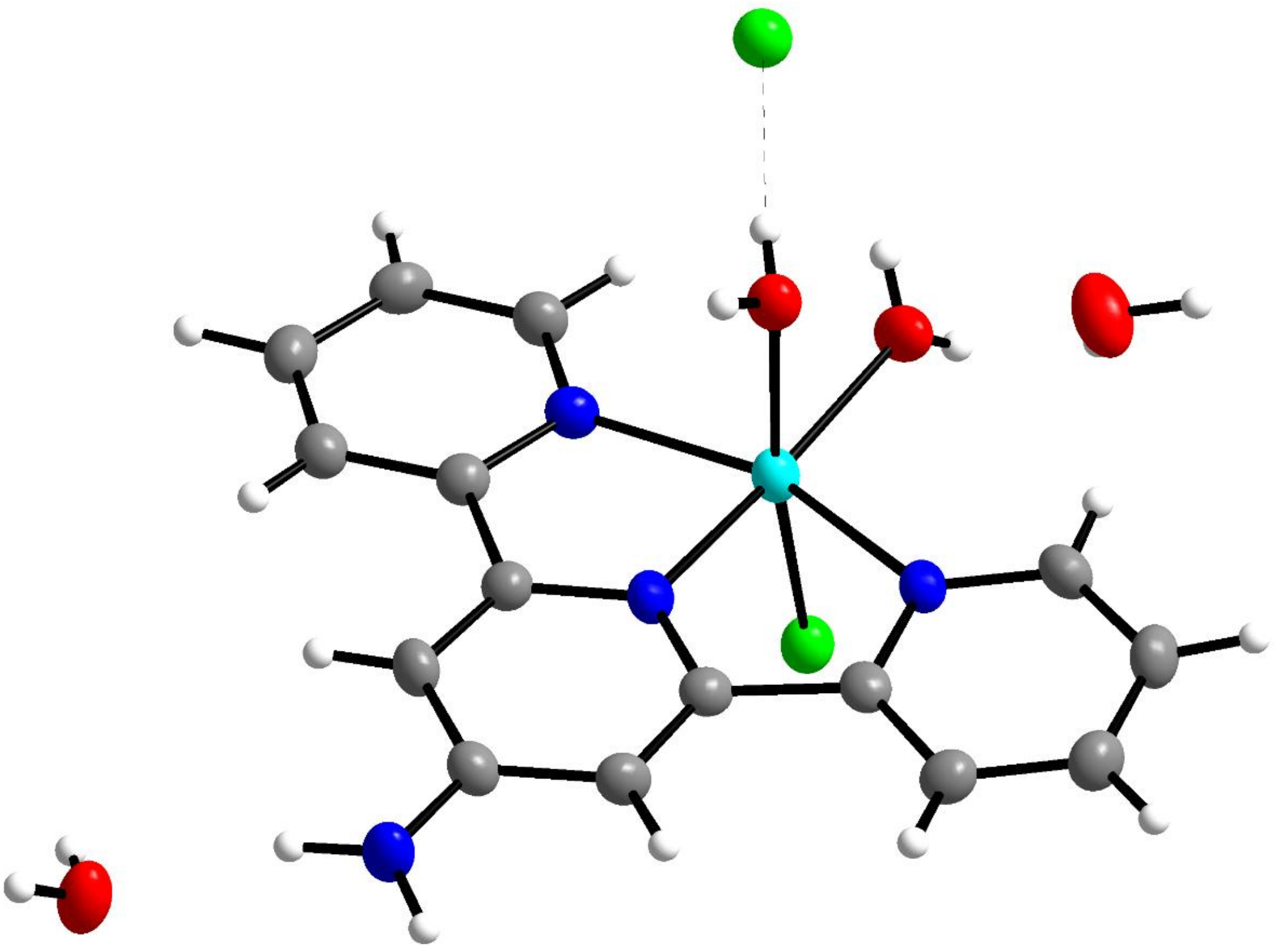

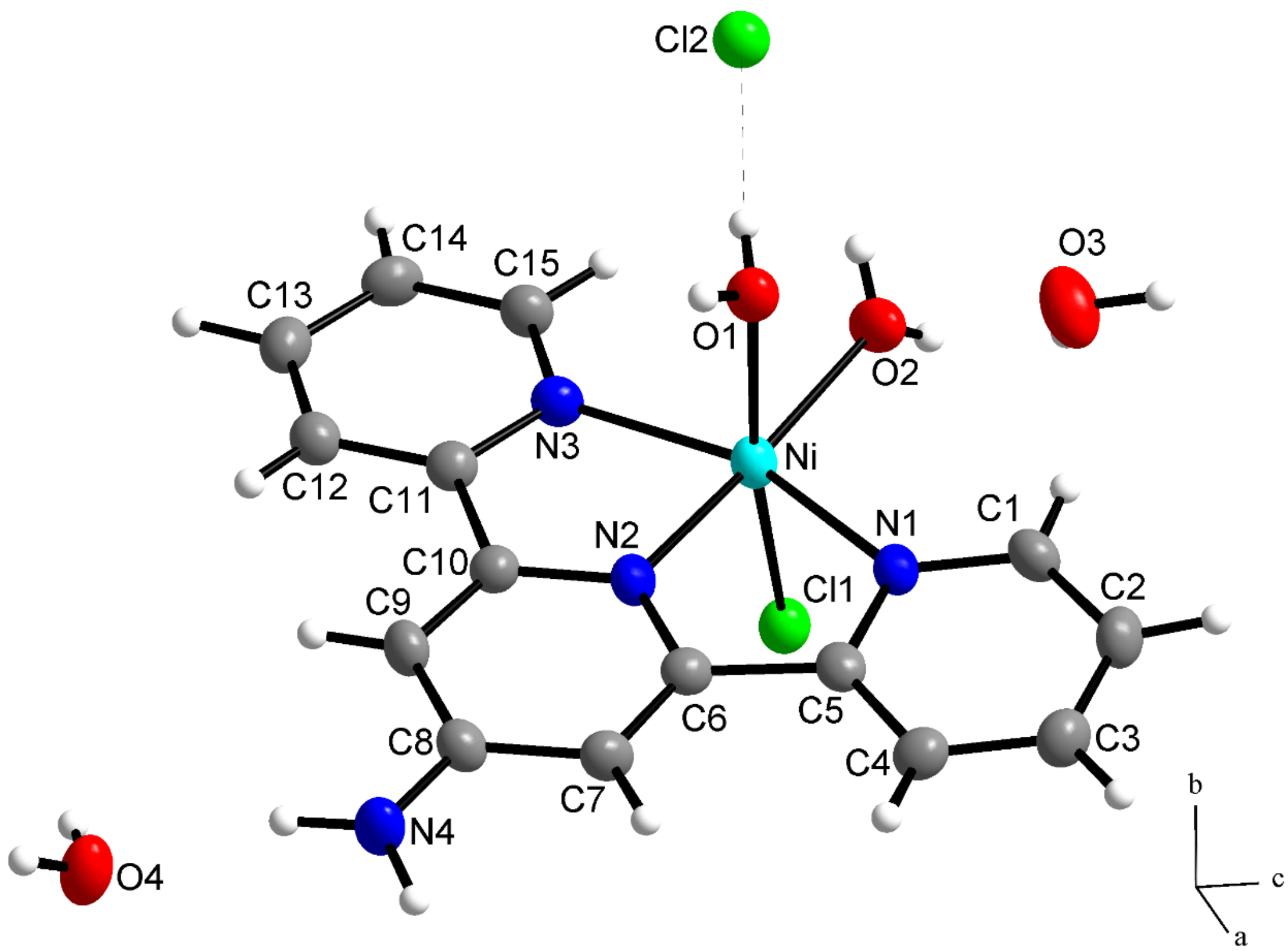

3: NitpyOH ($[Ni\ (C_{15}H_{11}ON_3)(H_2O)_3]Cl_2{\cdot}H_2O$)

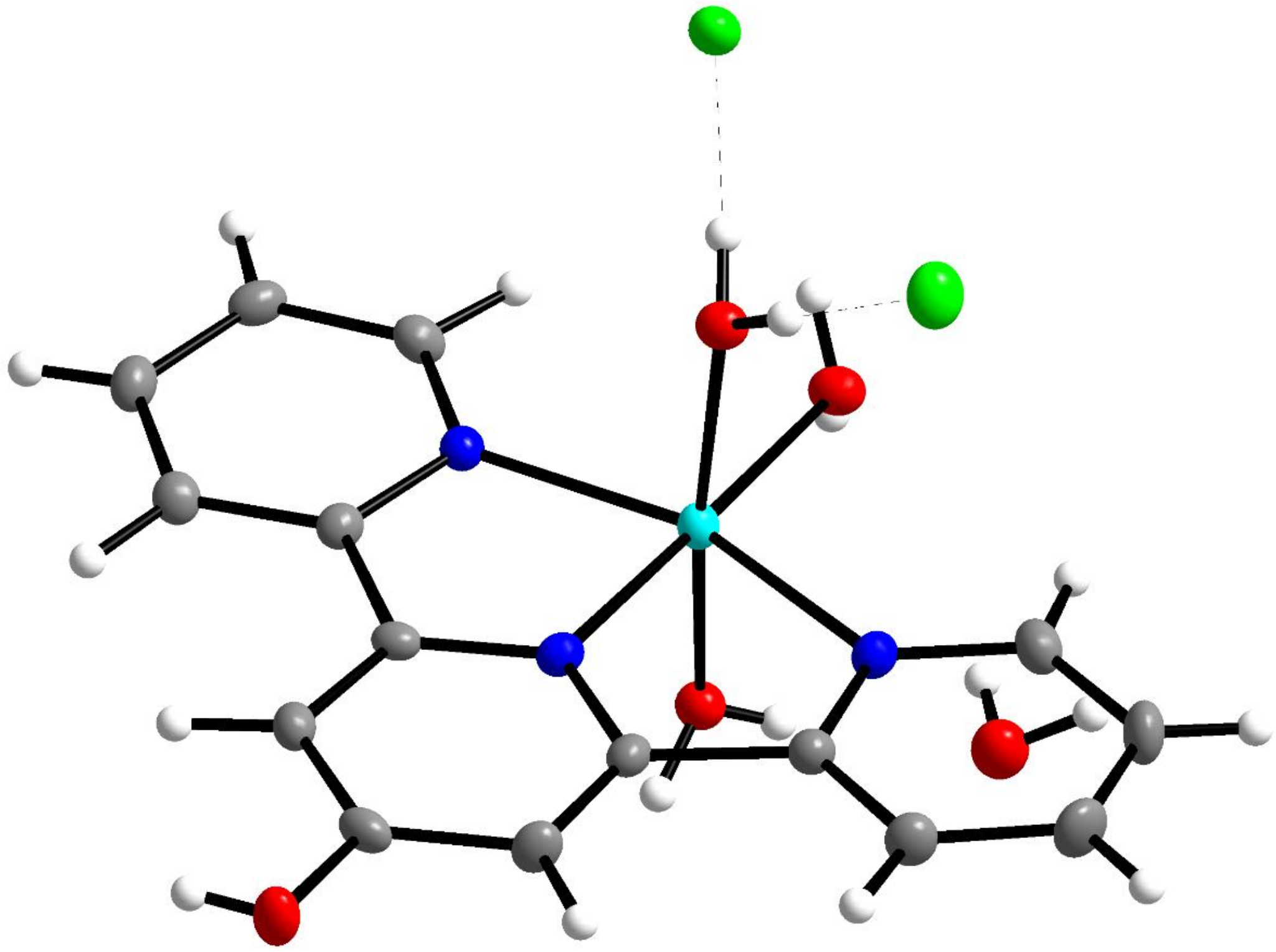

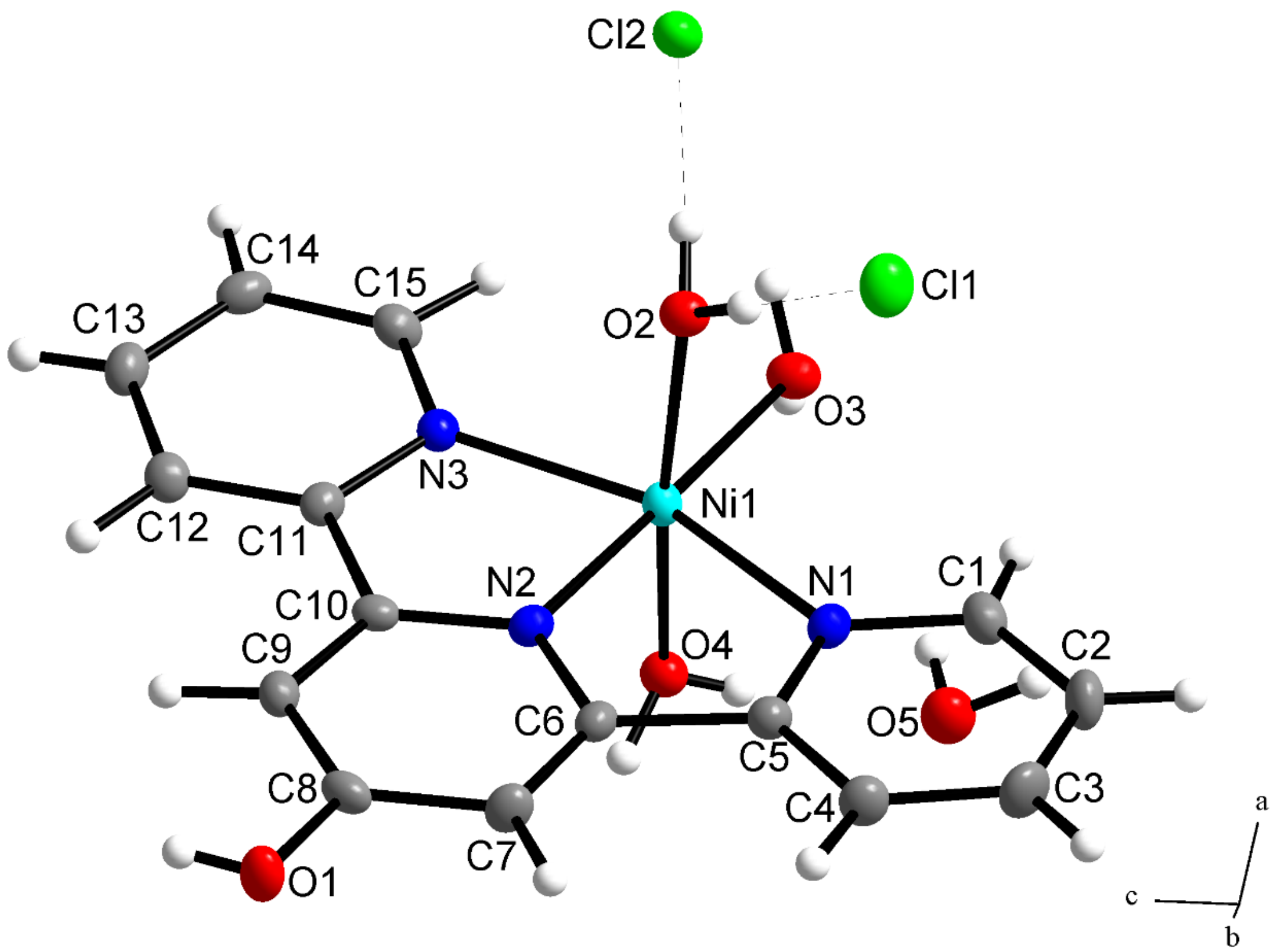

4: NitpyOCH3 ([Ni ($C_{16}H_{13}ON_3$)Cl(H_2O)$_2$]Cl·2H_2O)

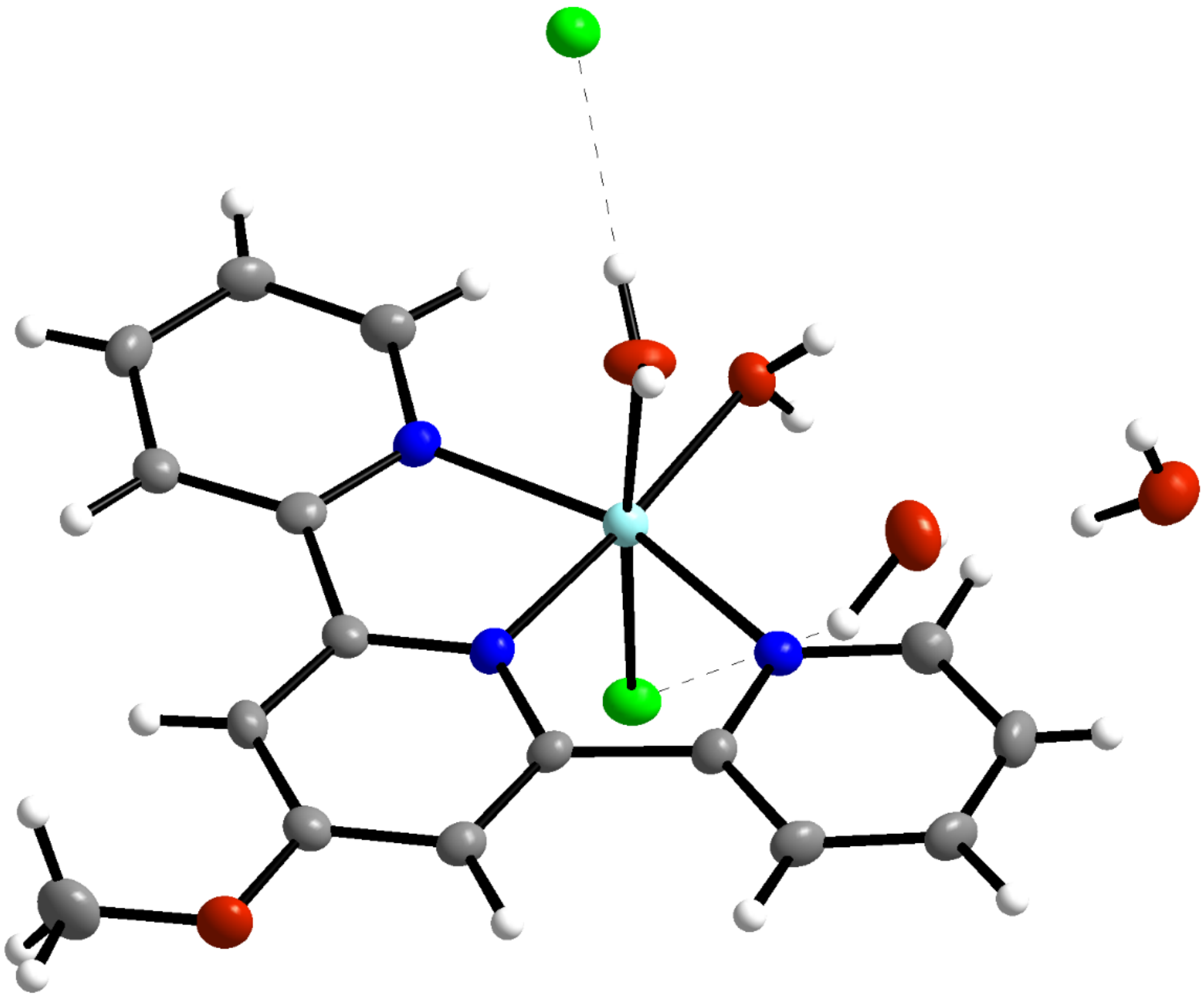

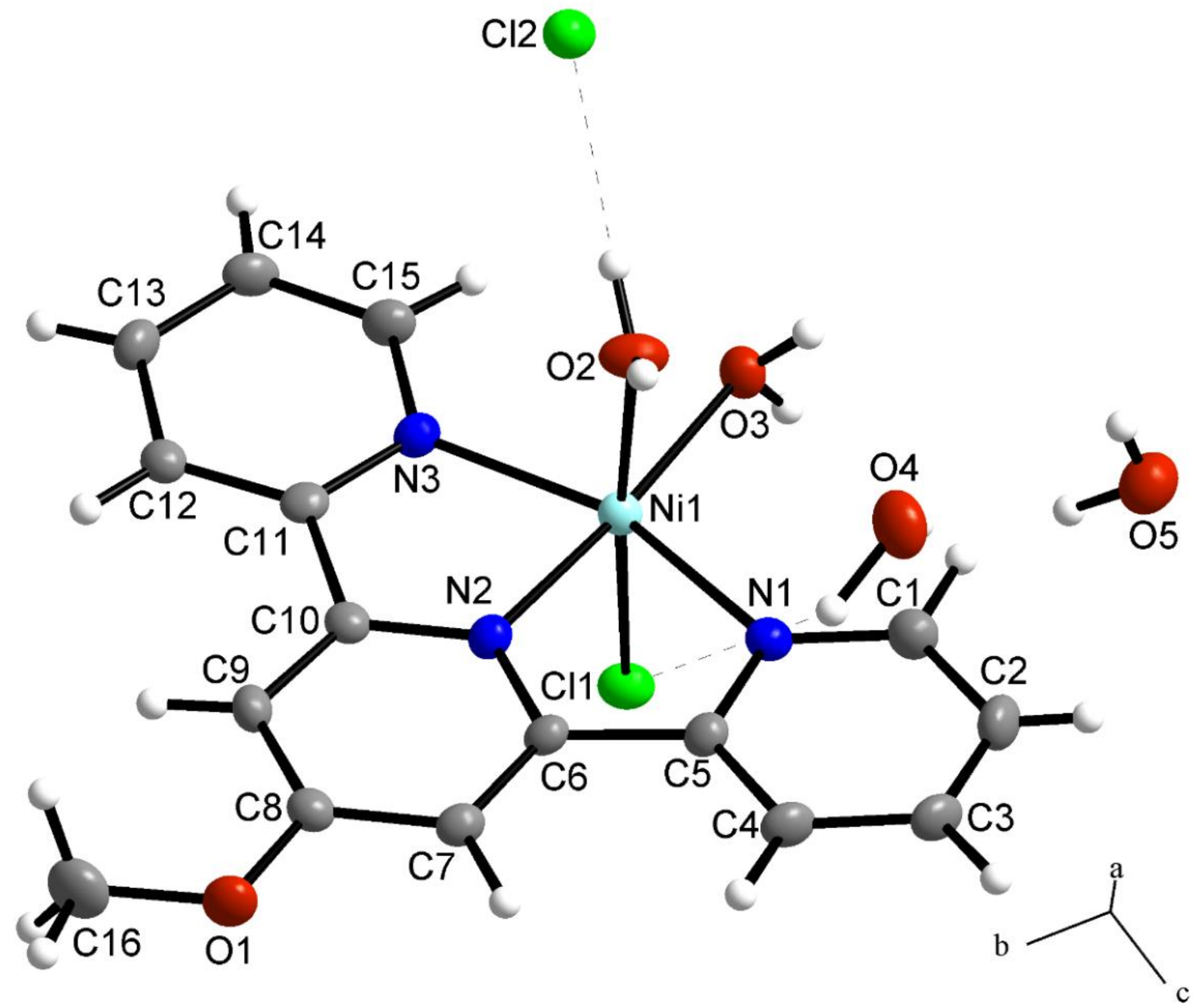

5: (NitpyOH)$_2$ (1/2[Ni ($C_{15}H_{11}ON_3$)Cl H_2O]$_2$Cl· CH_3OH· H_2O)

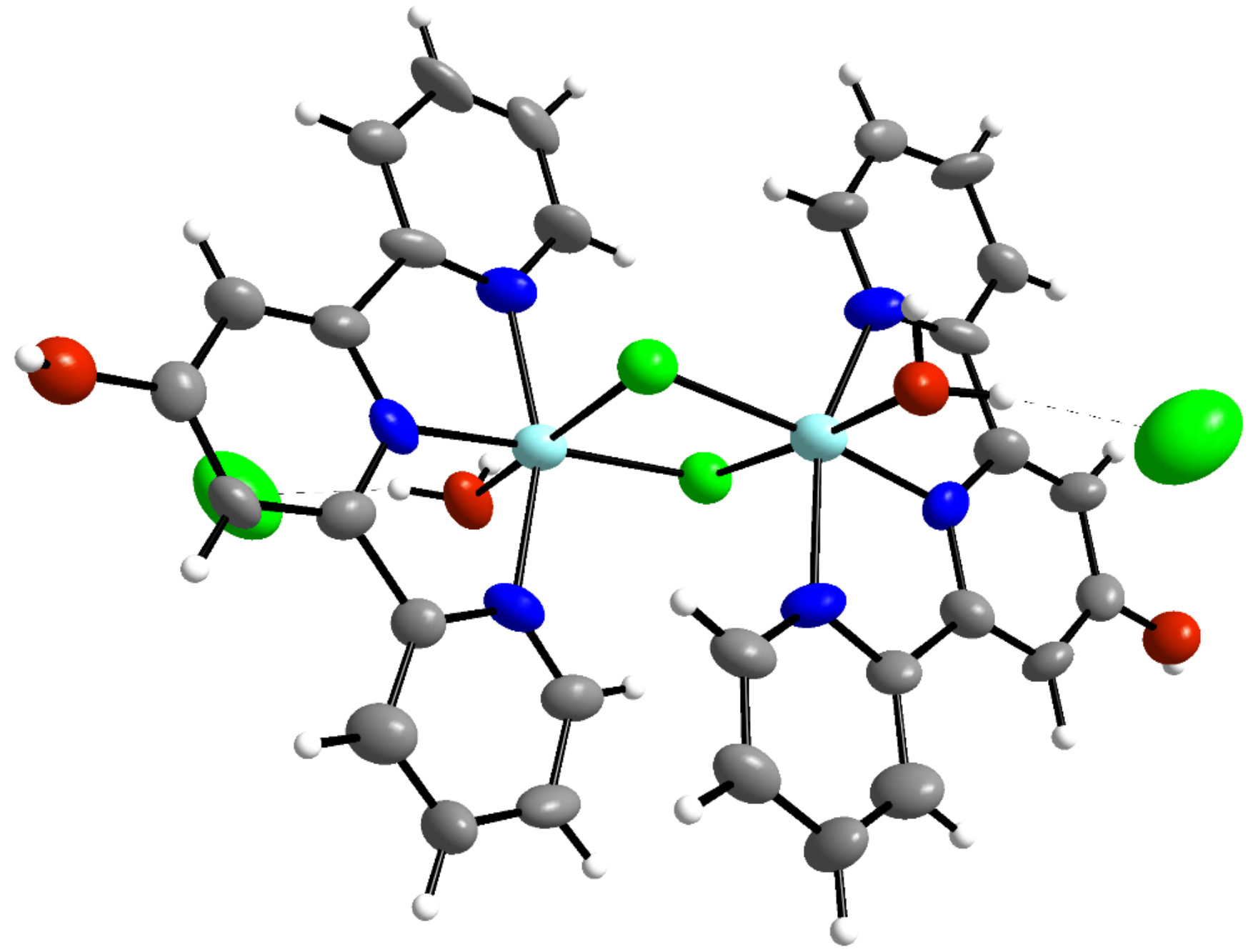

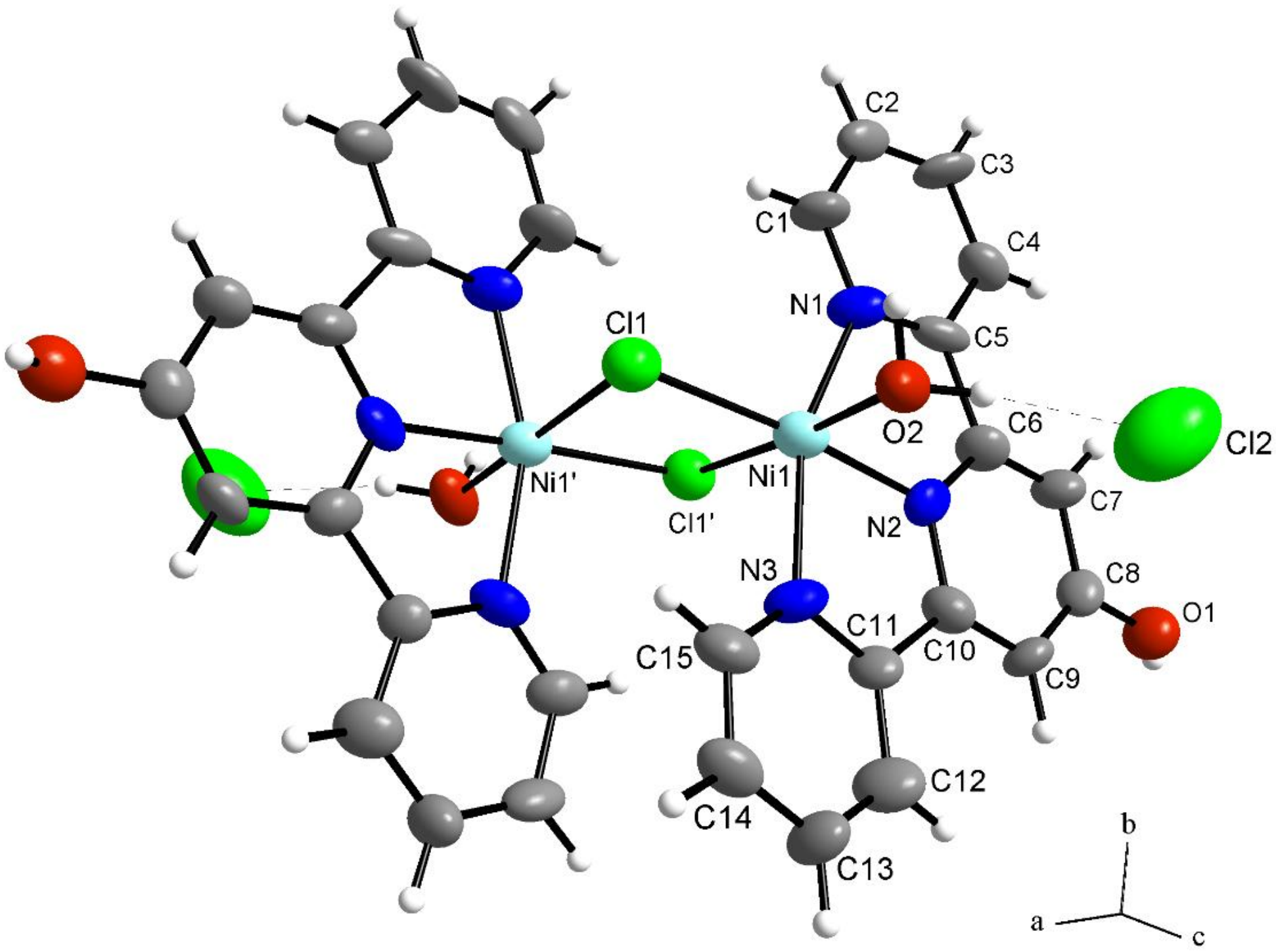

6: (NitpyOCF3)$_2$ (1/2[Ni ($C_{16}H_{10}F_3N_3$)Cl(H_2O)]$_2$Cl · SO(CH_3)$_2$)

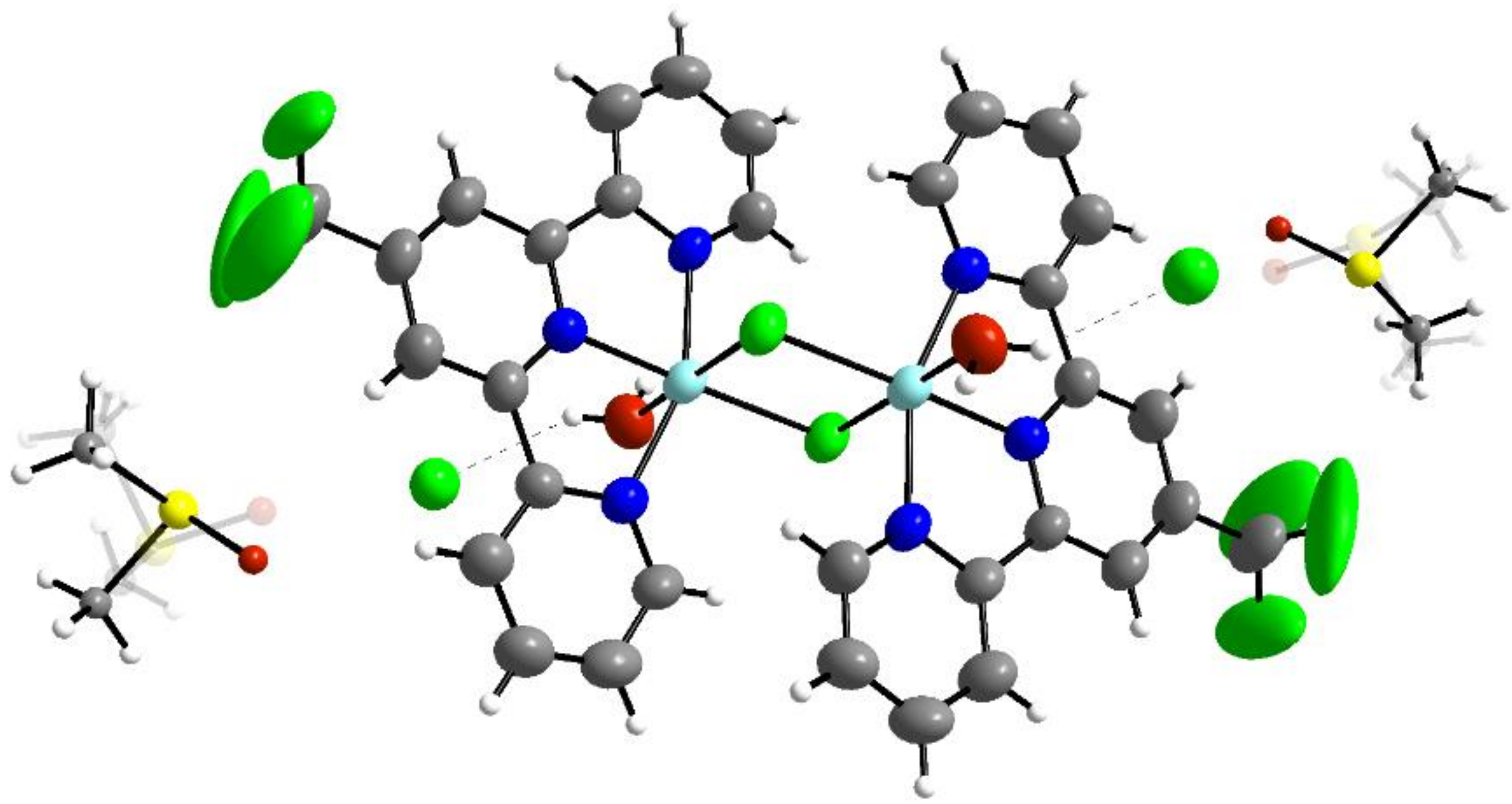

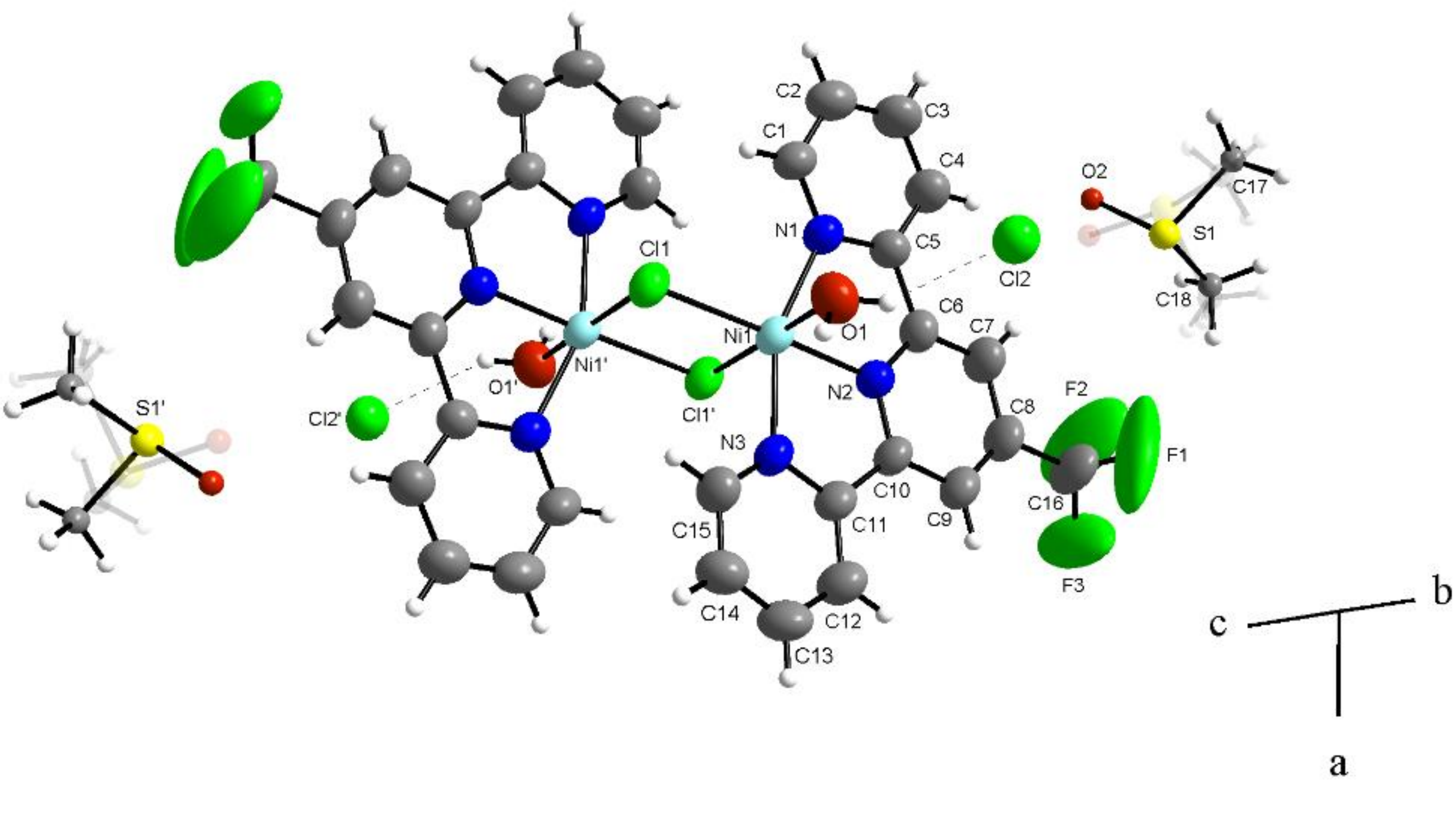

Table S4. FE, partial current density and production rate for ammonia using a PMR coupled with NitpyOH with different reactants at an applied current of -1 mA.

Reactants	$FE_{Ammonia}$ (%)	$j_{Ammonia}$ (mA cm^{-2})	Product rate (mM h^{-1})
N_2H_4	2.39	0.0239	0.44

Table S5. Select molecular electrocatalysts for N_2RR.

Catalyst	Main product	Electrolyte	Maximum partial current density (mA/cm^2)	Maximum Faradaic fficiency (%)	Reference
FePc/O-MWCNT	NH_3	0.1 M HCl	0.0365	9.73	*Chem. Commun.*, 2019, **55**, 14111--14114
MnPc	NH_3	0.1 M HCl	-	31.3	*Engineering Reports*, 2024**, 6,** e12705.
MnPc/C	NH_3	0.1 M HCl	0.724	35.3	*Engineering Reports*, 2024**, 6,** e12705.
Ni_3S_8 complex	N_2H_4	0.1 M $TBAPF_6$ in THF	-	~8-12	*J. Am. Chem. Soc*. 2020, **142**, 17312−17317
Mo-PNP	NH_3	0.1 M Li NTf_2 in THF	-	43	*ACS Catal*. 2023, **13**, 72−78

Simulation results

Molecular DFT:

Table S6. The redox potentials for N_2RR intermediates over NitpyOH molecular catalysts.

Reaction intermediate	Redox potential (V)
*N-NH	-5.12
*NH-NH	-5.24
*NH-NH_2	-3.11
*NH_2-NH_2	-4.54
*NH_2	-2.5
*NH_3	-4.5

Periodic DFT

The reaction energies calculated from the periodic DFT calculations are shown in Figure S50, which illustrates that the first protonation step to form *N-NH species as the potential determining step. The reaction energy for this step was calculated to be 0.55 eV. The reaction energies for the following reaction steps involving *NH-NH, *NH-NH_2, *NH_2-NH_2 and *NH_2 were downhill demonstrating the energetic favourability of these intermediates. Further, we have considered the first three reaction steps for evaluating the free energies of the reaction from the phonon frequency calculations. The resulting free energies of these steps are represented in the free energy diagram shown in Fig S50. The reaction free energy (0.766 eV) obtained from the periodic DFT represents the close agreement with the ΔG_r of 0.68 eV calculated from molecular DFT. The slight change in ΔG_r between both the DFT methods was due to use of different exchange and correlation functionals and basis set in addition with the reaction environment. The periodic calculations were performed under vacuum while the molecular DFT calculations carried out with SMD solvation model.

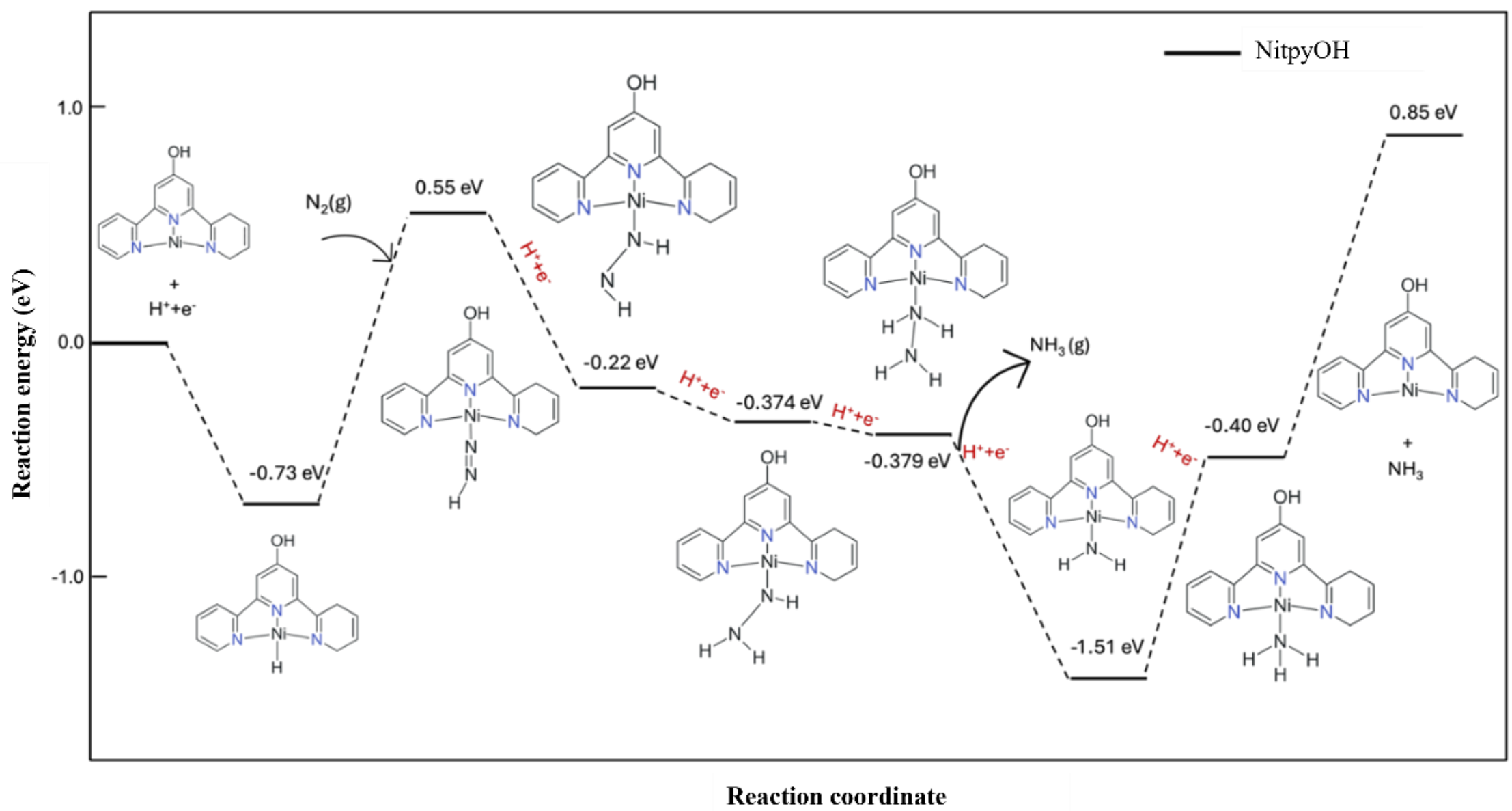

Figure S50. The reaction energy diagram for electrocatalytic N_2RR over NitpyOH catalyst from periodic DFT.

Additionally, we have performed the periodic DFT calculations for pure Nitpy molecular catalysts and with $-NH_2$ functional group. The corresponding reaction energies are tabulated in Table S7.

Table S7. Reaction energies for the N_2RR intermediates over pure Nitpy and $NitpyNH_2$ catalysts.

Reaction intermediate	Expression for reaction energy	Pure Nitpy (eV)	$NitpyNH_2$ (eV)
*N-NH	$\Delta E = [E(*N\text{-}NH) – E(*N_2) – E(1/2H_2)$	0.562	0.554
*NH-NH	$\Delta E = [E(*NH\text{-}NH) – E(*N\text{-}NH) – E(1/2H_2)$	-0.247	-0.213
$*NH\text{-}NH_2$	$\Delta E = [E(*NH\text{-}NH_2) – E(*NH\text{-}NH) – E(1/2H_2)$	-0.366	-0.383
$*NH_2\text{-}NH_2$	$\Delta E = [E(*NH_2\text{-}NH_2) – E(*NH\text{-}NH_2) – E(1/2H_2)$	-0.405	-0.349
$*NH_2$	$\Delta E = [E(*NH_2) + E(*NH_3) – E(*NH_2\text{-}NH_2) – E(1/2H_2)$	-1.5	-1.547
$*NH_3$	$\Delta E = [E(*NH_3) – E(*NH_2) – E(1/2H_2)$	-0.43	-0.378
NH_3 (g)	$\Delta E = [E(*) + E(*NH_3) – E(* + NH_3)$	0.71	0.83

In summary, both molecular and periodic DFT calculations were employed to provide a complementary understanding of the nitrogen reduction mechanism over the NitpyOH catalyst. The molecular DFT approach allowed us to accurately describe the local coordination environment of the Ni center, evaluate adsorption energies, and identify key intermediates such as Ni-H and *N-NH species. In contrast, the periodic DFT calculations capture extended interactions between the molecular catalyst and the surrounding surface or reaction environment, offering insight into the overall thermodynamic landscape and potential energy barriers under more realistic conditions. The good agreement between both approaches, in particular for the potential-determining *N–NH formation step, supports the reliability of our computational findings and strengthens the proposed mechanistic picture in which Ni-H formation is central to N_2 activation and subsequent hydrogenation steps.

References

1 Y. H. Budnikova, D. A. Vicic and A. Klein, *Inorganics (Basel)*, 2018, **6**, 18.

2 R. Hogg and R. G. Wilkins, *J Chem Soc*, 1962, 341–350.

3 Y. Zhao, D. G. Truhlar, *Theor. Chem. Acc.*, 2008, **120**, 215-241.

4 M. J. Frisch et al., Gaussian 16, C.01, Gaussian, Inc., Wallingford CT, 2016.

5 F. Weigend, R. Ahlrichs, *Phys. Chem. Chem. Phys.*, 2005, **7,** 3297-305.

6 M. Dolg, U. Wedig, H. Stoll, H. Preuss, *J. Chem. Phys.*, **86** (1987) 866-72.

7 A. V. Marenich, C. J. Cramer, and D. J. Truhlar, *J. Phys. Chem. B.*, **113** (2009) 6378-96.

8 Y. P. Li, J. Gomes, S. M. Sharada, A. T. Bell, M. Head-Gordon, *J. Phys. Chem. C*, 2015, **119**, 1840-1850.

9 S. Grimme, *Chem. Eur. J.*, 2012, **18**, 9955-9964.

10 J. K. NØrskov, J. Rossmeisl, A. Logadottir, L. Lindqvist, J. R. Kitchin, T. Bligaard, H. Jonsson, *J. Phys. Chem. B, 2004*, **108**, 46, 17886.

11 T. Lu, F. Chen, *J. Comput. Chem.*, 2012, 33, 580-592.

12 T. Lu, *J. Chem. Phys.*, 2024, 161, 082503.

13 O. López-Estrada, N. Mammen, L. Laverdure, M. M. Melander, H. Häkkinen, K. Honkala, ACS Catal., 2023, 13, 8997-9006.

14 J. P. Perdew, K. Burke, and M. Ernzerhof, *Phys. Rev. Lett.* 1996, **77**, 3865.

15 G. Kresse and J. Hafner, Phys. Rev. B 1993, **47**, 558; *ibid.* 1994, **49**, 14 251.

16 G. Kresse and J. Furthmüller, *Comput. Mat. Sci.* 1996, **6**, 15.

17 G. Kresse and J. Furthmüller, *Phys. Rev. B 1996*, **54**, 11 169.

18 G. Kresse and J. Hafner, *J. Phys.: Condens. Matt*. 1994, **6**, 8245.

19 G. Kresse and D. Joubert, *Phys. Rev.* 1999, **59**, 1758.

20 H. J. Monkhorst, J. D. Pack, *Phys. Rev. B*, 1976, 13, 5188-5192.

21 S. Grimme, J. Antony, S. Elrich, S. Krieg, *J. Chem. Phys.*, 2010, **132**, 154104.

22 S. Grimme, S. Elrich, L. Goerigk, *J. Comput. Chem.*, 2011, **32**, 1456.

23 F. Neese, WIRES Comput. Molec. Sci., 2025, **15**, e70019

23 F. Neese, J. Comp. Chem., 2003, **24**, 1740-1747

24 F. Neese, F. Wennmohs, A. Hansen, U. Becker, Chem. Phys., 2009, **356**, 98-109

25 E. Caldeweyher, C. Bannwarth, S. Grimme, J. Chem. Phys., 2017, **147**, 034112

26 E. Caldeweyher, S. Ehlert, A. Hansen, H. Neugebauer, S. Spicher, C. Bannwarth, S. Grimme, J. Chem. Phys., 2019, **150**, 154122

27 B. Helmich-Paris, B. de Souza, F. Neese, R. Izsák, J. Chem. Phys., 2021, **155**, 104109

28 F. Neese, J. Comp. Chem., 2022, **44**, 381
